# Poly(I:C)-exposed zebrafish shows autism-like behaviors which are ameliorated by *fabp2* gene knockout

**DOI:** 10.3389/fnmol.2022.1068019

**Published:** 2023-01-05

**Authors:** Jing Wu, Xueting Lin, Dian Wu, Binhong Yan, Mengyi Bao, Peilei Zheng, Jiangping Wang, Cuiwei Yang, Zhongxia Li, Xiaoming Jin, Kewen Jiang

**Affiliations:** ^1^Department of Child Psychology, The Children’s Hospital, Zhejiang University School of Medicine, National Clinical Research Center For Child Health, Hangzhou, China; ^2^Department of Biobank Center, The Children’s Hospital, Zhejiang University School of Medicine, National Clinical Research Center For Child Health, Hangzhou, China; ^3^Department of Neurology, The Children’s Hospital, Zhejiang University School of Medicine, National Clinical Research Center For Child Health, Hangzhou, China; ^4^Department of Pediatrics, The Seventh Affiliated Hospital of Guangxi Medical University (Wuzhou GongRen Hospital), Wuzhou, Guangxi, China; ^5^Indiana Spinal Cord and Brain Injury Research Group, Stark Neurosciences Research Institute, Department of Anatomy, Cell Biology and Physiology, Indiana University School of Medicine, Indianapolis, IN, United States; ^6^Stark Neuroscience Research Institute, Department of Neurological Surgery, Indiana University School of Medicine, Indianapolis, IN, United States

**Keywords:** autism, F0 knockout, zebrafish, social interaction, shoaling, *fabp2*

## Abstract

**Introduction:**

Autism spectrum disorder (ASD) is a group of neurodevelopmental disorders mainly representing impaired social communication. The etiology of ASD includes genetic and environmental risk factors. Rodent models containing ASD risk gene mutations or environmental risk factors, such as exposure to maternal inflammation, show abnormal behavior. Although zebrafish conserves many important brain structures of humans and has sophisticated and fine behaviors in social interaction, it is unknown whether the social behaviors of their offspring would be impaired due to exposure to maternal inflammation.

**Methods:**

We exposed zebrafish to maternal immune activation (MIA) by injection with polyinosinic:polycytidylic acid [poly(I:C)], and screened their behaviors through social behavioral tests such as social preference and shoaling behavior tests. We compared phenotypes resulted from different ways of poly(I:C) exposure. RNA sequencing was performed to explore the differential expression genes (DEGs). Gene ontology (GO), Kyoto Encyclopedia of Genes and Genomes (KEGG) and protein–protein interaction (PPI) network analysis was performed with the detected DEGs to find the concentrated pathways. Finally, we knocked out the *fatty acid-binding protein 2 (fabp2)*, a key node of the concentrated PPI network, to find its rescues on the altered social behavior.

**Results:**

We reported here that MIA offspring born to mothers injected with poly(I:C) exhibited impaired social approach and social cohesion that mimicked human ASD phenotypes. Both maternal exposure and direct embryo exposure to poly(I:C) resulted in activations of the innate immune system through toll-like receptors 3 and 4. RNA-sequencing results from MIA brain tissues illustrated that the numbers of overexpressed genes were significantly more than that of underexpressed genes. GO and KEGG analyses found that MIA-induced DEGs were mainly concentrated in complement and coagulation cascade pathways. PPI network analyses suggested that *villin-1* (*vil1*) pathway might play a key role in MIA-induced ASD. Knockout of *fabp2* in F0 zebrafish rescued the social behavior deficits in MIA offspring.

**Conclusions:**

Overall, our work established an ASD model with assessable behavior phenotype in zebrafish and provided key insights into environmental risk factor in ASD etiology and the influence of *fabp2* gene on ASD-like behavior.

## 1. Introduction

Autism spectrum disorder (ASD) is a group of neurodevelopmental disorders with genetic and clinical heterogeneity characterized by altered social communication, unusually restricted interests, and repetitive behavior (Lai et al., 2014). The etiology of ASD includes environmental and genetic risk factors (Lyall et al., 2017). Next-generation sequencing approaches have found a large number of high-risk genes in ASD (O'Roak et al., 2012; Yuen et al., 2017). Mice containing ASD risk gene mutations showed abnormal behavior, including social disorder (Ey et al., 2011; Crawley, 2012). Similar behavioral abnormalities were observed in mouse models of environmental risk factors for ASD, such as exposure to maternal inflammation (Patterson, 2011). These models deepen our understanding of the mechanisms underlying ASD at both molecular and neural levels. However, preparing mouse models of ASD is time-consuming and laborious, and the yield is limited, especially when a large number of disordered animals are needed such as for targeted drug screening.

The zebrafish (Danio rerio) is an easy-to-handle vertebrate model for neuroscience research (Kalueff et al., 2014). Recent evidence suggests that it conserves many important human brain structures, such as the amygdala, hippocampus and hypothalamus (Parker et al., 2013) and that it has a sophisticated and fine behavior in social interaction, anxiety, aggression, learning and cognition (Kalueff et al., 2014). Since zebrafish and human genomes are highly conserved with more than 80% of human disease genes presenting in zebrafish (Howe et al., 2013), zebrafish has been widely used as a useful tool to clarify the functions of human candidate genes for ASD. However, after knockout of human high-risk genes of ASD, zebrafish offspring does not always show an autism-like phenotype, which limits its use for testing novel treatment and for drug screening. Although it is possible to knockout multiple genes with a single embryo microinjection in this model (Parvez et al., 2021), the low success and high mortality rate is a significant challenge. On the other hand, immune alterations are shown to play key roles in the mechanism of ASD. Immune abnormalities are associated with increased risk of ASD in children (Atladóttir et al., 2010), occurrence of psychiatric and non-psychiatric comorbidities of ASD (Wakefield, 2002; Wakefield et al., 2005; Ashwood and Wakefield, 2006), and many other features of ASD including mood and sleep disturbances. In rodents, exposing to maternal inflammation, i.e., maternal immune activation (MIA; Patterson, 2011), impairs sociability in offspring and serves as an environmental risk factors-ASD model. Although zebrafish has multiple advantages for ASD research as mentioned above, it is unknown whether sociability would also be impaired in MIA offspring.

A zebrafish model of ASD that involves environmental risk factors and immune alterations would be valuable not only for studying the pathology, comorbidity, and underlying mechanisms of ASD, but also for treatment development and drug screening. Here, we established that the social behavior is impaired in zebrafish offspring exposed to MIA by comparing them with those of direct embryo exposure of environmental risk factor. Since the mechanisms underlying MIA-induced ASD has not been fully understood, we analyzed differential gene expressions (DEGs) and protein interactions in MIA-induced ASD zebrafish. Finally, we identified a gene [*fatty acid-binding protein 2* (*fabp2*)] that was the only one of the top 10 co-expressed differential genes of both MIA and *in vitro* exposure of environmental risk factor and interacted with the key node [*villin-1* (*vil1*), a gene encoding actin-binding proteins] of protein–protein interaction (PPI) network. It was upregulated in MIA offspring but downregulated in offspring with direct embryo exposure to environmental risk factor. Importantly, knockout of this gene rescued the social behavior deficits in both MIA and *in vitro* exposure of environmental risk factor offspring.

## 2. Materials and methods

### 2.1. Zebrafish care and husbandry

All experiments and animal handling were performed according to the Guide for the Care and Use of Laboratory Zebrafish by the China Zebrafish Resource Center and were approved by the Animal Care and Use Committee at Zhejiang University School of Medicine (16779). Wild-type (WT) zebrafish (*Danio rerio*) AB strain was housed in a modular zebrafish system (Haisheng, China), and all fish were kept in a 10-h dark / 14-h light cycle, and 28 ± 0.5°C filtered and UV sterilized water. For breeding, after keeping adult female and male fish separate overnight in a 1-L crossing tank, we released them by removing the divider at 8–9 am the next morning and collected the fertilized embryos within 1 h after their releasing. After being maintained in embryonic media E3 for 24 h, healthy embryos were selected and raised regularly (See Additional file 1: File S1).

### 2.2. MIA and polyinosinic:polycytidylic acid (poly(I:C)) *in vitro* exposure (PIVE)

Poly(I:C) was used to establish MIA in zebrafish as previously reported in pregnant dams (Kim S. et al., 2017). For PIVE, we evaluated the toxicity of poly(I:C) by using embryos of 1–6 days post-fertilization (dpf; Aspatwar et al., 2019). In brief, healthy fertilized embryos (1 dpf) were randomly placed into a 200 μl volume of E3 or E3 with gradient concentrations of poly(I:C) (528,906, Millipore; 10, 50, 100, 250, 500, 750, or 1,000 μM) for the next consecutive 5 days. The embryos/larvae were carefully checked daily and the numbers of death, morphological defects of individuals such as absence of swim bladder, skeletal abnormality, pericardial edema and unhatched embryo, as well as the individuals of abnormal movement pattern were checked with a stereo microscope and recorded. The half-maximal lethal concentrations (LC50) were calculated based on above evaluation (Additional file 2: Figure S1).

A modified MIA by poly(I:C) injection without infections of segmented filamentous bacteria was used (Chow et al., 2016; Kim S. et al., 2017). In brief, a healthy adult female zebrafish received a single intraperitoneal injection (i.p.) of poly(I:C) (20 μg/g or 50 μg/g; about 5 μl) or received an injection of the same volume of vehicle (phosphate buffered solution (PBS)) as control. Basing on our results of poly(I:C)-indued immune response (Tsukada et al., 2021), we chose to mate the fish in 24 h after Poly(I:C) injection. For injection (i.p.), after a fish was hypothermic anesthetized, a needle (tip 0.1 μm) was gently inserted into the midline of the abdomen behind the pectoral fins to a depth of 3 mm. After injection, it was put back into reproductive water (28°C). Normally, the fish would recover free swimming within 1 min; otherwise, the fish was not used for mating (Samaee et al., 2017). We collected fertilized embryos within 1 h after mating and the healthy embryos were collected the next morning and raised normally. For PIVE, after establishing the LC50 (200.7 μM) of poly(I:C), we used concentrations lower than LC50 in our experiments, which resulted in little visible morphological defects and low mortality. The fertilized embryos (1 dpf) from the wild type (WT) strain were raised in E3 with poly(I:C) (10, 50, or 100 μM separately; final volume of 200 μl for each embryo by using 96-well plate) for the next consecutive 5 days or in E3 with the same volume of vehicle. Embryos/larvae were examined daily under a stereo microscope, and the numbers of death, morphological defects and abnormal movement-pattern were recorded. The fish were raised under normal husbandry from 6 dpf.

### 2.3. Measurement of immune response parameters

**Figure 1:**
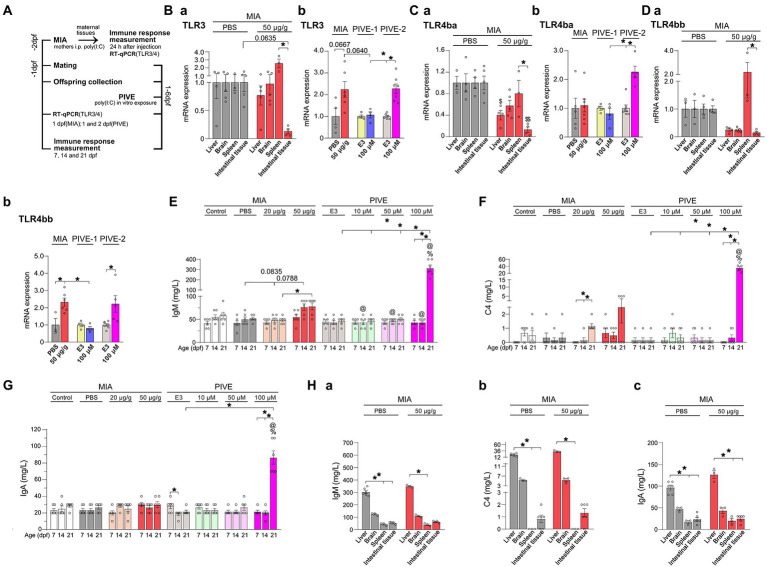
MIA offspring born to mothers injected with poly(I:C) showed an immune response. **(A)** For the maternal immune response induced by poly(I:C) (MIA-50 μg/g), 24 h after injection, TLR3, TLR4ba, and TLR4bb genes were selected and detected by quantitative RT-PCR, and the immunoglobulin M (IgM), complement C4 (C4) and immunoglobulin A (IgA) levels were analyzed by the immunoturbdimetric assay method. For MIA offspring TLR3, TLR4ba, and TLR4bb gene levels and IgM, C4 and IgA levels were checked at 1 dpf; as well as for PIVE, TLR3, TLR4ba and TLR4bb gene levels and IgM, C4, and IgA levels were tested at 1 dpf (PIVE-1) or 2 dpf (PIVE-2) after a 24 h poly(I:C) treatment. **(Ba)** TLR3 mRNA expression was up-regulated in spleen of maternal fish of MIA-50 μg/g group (*U* = 0, *p* = 0.0635) compared to that of MIA-PBS group; TLR3 mRNA expression was significantly down-regulated in intestinal tissue compared to that in spleen of maternal fish of MIA-50 μg/g group (*Z* = 3.37, *p* < 0.05). **(Bb)** TLR3 mRNA expression was up-regulated of MIA offspring (*U* = 3.30, *p* = 0.0667) compared to that of MIA-PBS group; For PIVE, there was no significant change in TLR3 mRNA expression of the offspring collected at 1 dpf after a 24 h poly(I:C) treatment; while TLR3 mRNA expression of the offspring collected at 2 dpf after a 24 h poly(I:C) treatment was significantly up-regulated. **(Ca)** TLR4ba mRNA expression was significantly down-regulated in intestinal tissue and liver of maternal fish of MIA-50 μg/g group compared to that of MIA-PBS group (Two-way ANOVA, *F* = 32.07 for MIA and *F* = 2.40 for Tissue; multiple comparisons test: *t* = 5.04 and 3.06, *p* < 0.01 and 0.05, in intestinal tissue and liver, respectively). TLR4ba mRNA expression was significantly down-regulated in intestinal tissue compared to that in spleen of maternal fish of MIA-50 μg/g group (*t* = 3.41, *p* < 0.05). **(Cb)** TLR4ba mRNA expression did not changed of MIA offspring compared to that of MIA-PBS group; For PIVE, there was no significant change in TLR4ba mRNA expression of the offspring collected at 1 dpf after a 24 h poly(I:C) treatment; while TLR4ba mRNA expression of the offspring collected at 2 dpf after a 24 h poly(I:C) treatment was significantly up-regulated (*U* = 0.00, *p* < 0.05). **(Da)** TLR4bb mRNA expression was significantly down-regulated in intestinal tissue compared to that in spleen of maternal fish of MIA-50 μg/g group (*Z* = 2.89, *p* < 0.05). **(Db)** TLR4bb mRNA expression was up-regulated of MIA offspring (*U* = 1.00, *p* < 0.05) compared to that of MIA-PBS group; For PIVE, there was no significant change in TLR4bb mRNA expression of the offspring collected at 1 dpf after a 24 h poly(I:C) treatment; while TLR4bb mRNA expression of the offspring collected at 2 dpf after a 24 h poly(I:C) treatment was significantly up-regulated (*U* = 1.00, *p* < 0.05). **(E)** The levels of IgM were increased of MIA-50 μg/g offspring at 14 dpf (*Z* = 2.46, *p* = 0.08, compared to that of MIA-PBS group). The levels of IgM were significantly increased of PIVE-100 μm larvae at 21 dpf (*Z* = 3.14, *p* < 0.05, compared to that of PIVE-E3 group). **(F)** The levels of C4 were not changed significantly of MIA-50 μg/g offspring. The levels of C4 were significantly increased of PIVE-100 μm larvae at 21 dpf (*Z* = 3.43, *p* < 0.05, compared to that of PIVE-E3 group). **(G)** The levels of IgA were not changed significantly of MIA-50 μg/g offspring. The levels of IgA were significantly increased of PIVE-100 μm larvae at 21 dpf (*Z* = 3.58, *p* < 0.05, compared to that of PIVE-E3 group). **(Habc)** IgM, C4 and IgA levels were not changed significantly of maternal fish of MIA-50 μg/g group compared to that of MIA-PBS group. Data are presented as mean ± SEM. ^*^p < 0.05; ^$^ *p* < 0.05 compared with the MIA-PBS group of the same age; ^%^ *p* < 0.05 compared with the MIA-20 μg/g group of the same age; ^@^ *p* < 0.05 compared with the MIA-50 μg/g group of the same age. Detail about the *n* and descriptive data for each group and statistical analysis results could be found in Additional file 11: File S4.

For maternal immune response induced by poly(I:C) at 24 h after injection, the fish were anesthetized by low-temperature, and the liver, brain, spleen and intestine tissues were collected, quickly frozen and stored at −80°C. There were 1–2 biological repeat samples in each group (*n* = 1–2), which was a mixture of tissue samples of 7 zebrafish individuals (20 mg) (Figure 1A). For immune response of MIA offspring or PIVE larvae, the fish were anesthetized at 3, 7, 14, and 21 dpf, respectively. There were two biological repeat samples in each group (*n* = 2), which was a mixture of 10–15 zebrafish individuals (20 mg) (Figure 1A). The frozen samples were thawed in ice, homogenized in ice-cold RIPA (200 μl per 20 mg tissue), and then centrifuged to obtain the supernatant (15,000 rpm, 4°C, 30 min) for further measurement. The immunoglobulin M (IgM), complement C4 (C4) and immunoglobulin A (IgA) levels were analyzed by using the kits (Nanjing Jiancheng Bioengineering Institute, Nanjing, China) according to the manufacturer’s protocols. An immunoturbidimetric assay method was used to measure the agglutinate produced by the reaction at 340 nm wavelength (Hitachi 7180 biochemical analyzer).

Poly(I:C) can activate host immune defense through toll-like receptor (TLR). To determine immune response to the MIA/PIVE treatment, TLR3, TLR4ba and TLR4bb genes were detected by a reverse transcription quantitative PCR (RT-qPCR) (see detail in 2.7 section). At 24 h after the poly(I:C) injection, 3–5 maternal fish, as well as at 1 dpf, 3–5 offspring were collected from the MIA-PBS and MIA-50 μg/g groups. For PIVE, 3–5 offspring were collected from the PIVE-E3 and PIVE-100 μM groups at 1 dpf (PIVE-1) or 2 dpf (PIVE-2) after a 24 h poly(I:C) treatment (Figure 1A).

### 2.4. Behavioral analysis

**Figure 2:**
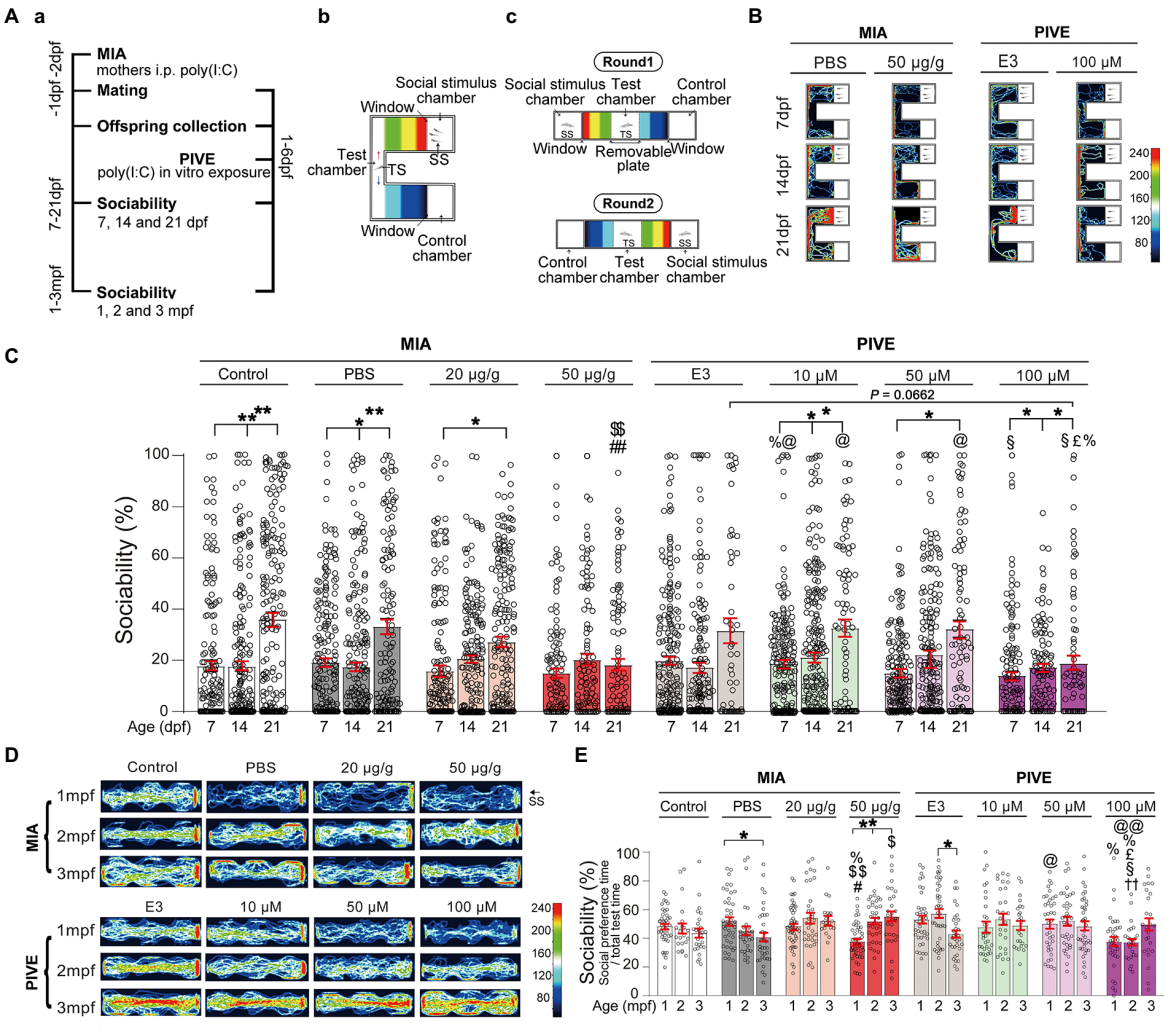
MIA offspring exhibited impaired social approach. **(Aa)** Zebrafish were tested for sociability (percentage of time spent investigating social object/total test time spent) at 7, 14, and 21 dpf **(Ab)**, and 1, 2, and 3 mpf **(Ac)** after maternal poly(I:C) injection (MIA) or PIVE to fertilized embryos. **(B)** Video tracking of movements of MIA and PIVE larvae at 7, 14, and 21 dpf, showing the social interaction with social cue. **(C)** Sociability of MIA and PIVE larvae at 7, 14, and 21 dpf. MIA impaired the sociability at 21 dpf in 50 μg/g group (*Z* = 4.16 and 4.06, *p* < 0.01, compared to that of MIA-Control and MIA-PBS group, respectively); PIVE impaired the sociability at 21 dpf of 100 μM group (*Z* = 2.54, *p* = 0.07, compared to that of PIVE-E3 group). **(D)** Video tracking of movements of MIA and PIVE larvae at 1, 2, and 3 mpf, showing the social interaction with social cue. **(E)** Sociability of MIA and PIVE larvae at 1, 2, and 3 mpf. MIA decreased the sociability levels at 1 mpf of 50 μg/g group (*Z* = 3.59 and 3.99, *p* < 0.05 and 0.01, compared to that of MIA-Control and MIA-PBS group respectively); PIVE impaired the sociability at 2 mpf of 100 μM group (*Z* = 3.74, *p* < 0.01, compared to that of PIVE-E3 group). Data are presented as mean ± SEM. ^*^ *p* < 0.05, ^**^ *p* < 0.001; ^#^ *p* < 0.05, ^##^ *p* < 0.001 compared with the MIA-control group of the same age with social stimulus; ^$^ *p* < 0.05, ^$$^ *p* < 0.001 compared with the MIA-PBS group of the same age with social stimulus; ^%^ *p* < 0.05 compared with the MIA-20 μg/g group of the same age; ^@^ *p* < 0.05， ^@@^ *p* < 0.001 compared with the MIA-50 μg/g group of the same age. ^††^ *p* < 0.001 compared with the PIVE-E3 group of the same age with social stimulus. ^§^ *p* < 0.05 compared with the PIVE-10 μM group of the same age; ^£^ *p* < 0.05 compared with the PIVE-50 μM group of the same age. TS, Test subject; SS, Social stimulus. Detail about the *n* and descriptive data for each group and statistical analysis results could be found in Additional file 11: File S4.

During a light cycle, the fish were transferred to a testing room equipped with a high-definition digital camera at least 1 h before the initiation of experiments. Tracking of fish behavior was done by using the ImageJ with a wrMTrck plugin (Selvaraj et al., 2019). We analyzed the developmental changes of fish behavior for both MIA and PIVE. For larvae, the social preference test, shoaling behavior and inattentive behavior test were used at 7, 14, and 21 dpf; for juvenile and adult zebrafish, the social preference test, shoaling behavior, open field test (OFT), novel tank test (NTT) and mirror test were used at 1, 2, and 3 month(s) post-fertilization (mpf) (Figures 2Aabc, 3Aab, 4A, 5Aabcd). Detailed information about the test tools is provided in Additional file 3: File S2. For all behavioral analysis, the test fish must be healthy. Those with obvious deformities or immobility within the test time were excluded from further analysis.

#### 2.4.1. Social preference test

The social preference test for larvae was carried out as described in Dreosti et al. (2015). We used a transparent U-shaped plastic tank containing three compartments: a central area (bottom of U-shaped tank), the left side and the right side of the U-shaped tank, which were separated by two glass barriers located at both side of U-shaped tank (3 cm away from the bottom). Briefly, a single fish was introduced into the central area for 1 min and permitted to interact with a group of three fish placed in one of two side compartments for another 6 min. The sociability wherein the social preference was an index based on subtracted non-social exploration, i.e., the time of the fish spent closest to the group of strangers divided by total test time (Figures 2Ab,C) or total interaction time (the time spent closest to social stimulus + the time near the empty area diagonally opposite; Additional file 2: Figure S2) and expressed as a percentage. For juvenile and adult zebrafish, we used a transparent plastic tank containing five compartments (separated by transparent plastic plates): the stimulus-fish area, near stimulus-fish area, central area, away from stimulus-fish area and reference area (Ogi et al., 2021; Figure 2Ac). Briefly, after a single fish was introduced into the central area for 30 s, the plates between the near/away stimulus-fish area was removed and let the fish stay in the area for another 30s (for acclimation). Then it was permitted to interact with a single strange fish placed in stimulus-fish area for another 10 min. To avoid moving direction preference, we randomly put a stimulus fish into the stimulus area on one side (Round 1). After 10-min behavioral recording, the same fish’s behavior was checked with the stimulus fish put on the opposite side (Round 2). The sociability was defined as the time of the fish spent in the near stimulus-fish area averaged from round 1 and 2, divided by total test time (Figures 2Ac,E) or total interaction time (the time spent closest to social stimulus + the time near the empty area diagonally opposite; Additional file 2: Figure S2) and expressed as a percentage.

#### 2.4.2. Shoaling behavior

**Figure 3:**
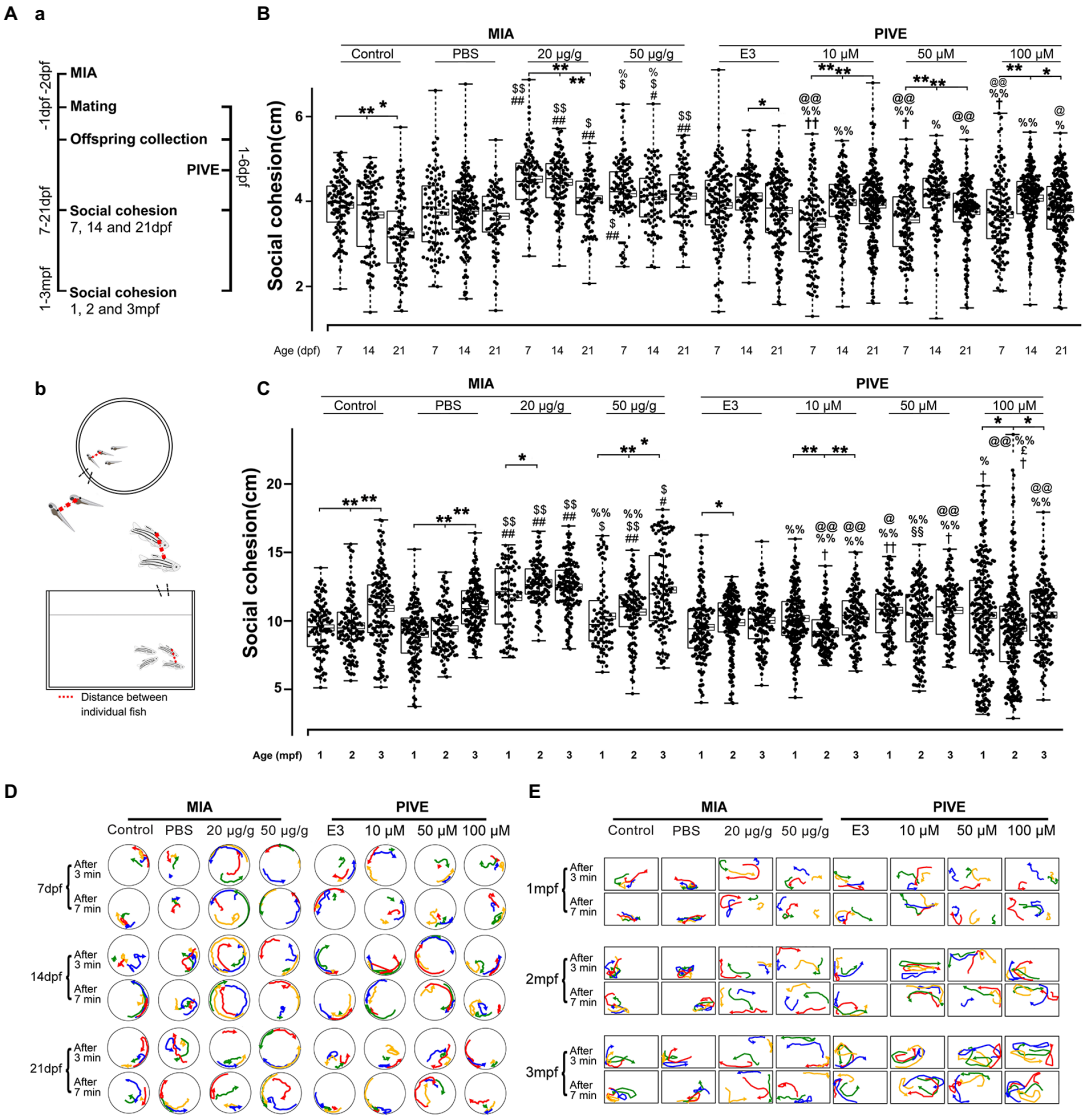
MIA offspring exhibited decreased social cohesion. **(Aa)** Zebrafish were tested for social cohesion (distance between individual fish) at 7, 14, and 21 dpf, and 1, 2, and 3 mpf **(Ab)** after maternal poly(I:C) injection (MIA) or PIVE to fertilized embryos. **(B)** Both MIA-20 and 50 μg/g induced a decreased social cohesion at 7, 14, and 21 dpf (MIA-20 μg/g: *Z* = 5.66, 6.31, and 5.57, *p* all <0.01 for 7, 14, and 21 dpf vs. MIA-Control; *Z* = 6.66, 7.45, and 3.59, *p* < 0.01, 0.01, and 0.05, respectively, for 7, 14, and 21 dpf vs. MIA-PBS. MIA-50 μg/g: *Z* = 2.54, 2.68 and 5.95, *p* = 0.07, <0.05 and 0.01, respectively, for 7, 14, and 21 dpf vs. MIA-Control; *Z* = 3.69, 3.30, and 4.01, *p* < 0.05, 0.05, and 0.01, respectively, for 7, 14, and 21 dpf vs. MIA-PBS) but not for PIVE fish. **(C)** Both MIA-20 and 50 μg/g induced a decreased social cohesion at 1, 2, and 3 mpf (MIA-20 μg/g: *Z* = 5.76, 10.93, and 7.72, *p* all <0.01 for 1, 2, and 3 mpf vs. MIA-Control; *Z* = *Z* = 7.46, 11.63, and 5.77, *p* all <0.01 for 1, 2 and 3 mpf vs. MIA-PBS. MIA-50 μg/g: *Z* = 1.60, 4.06 and 3.48, *p* > 0.05, <0.01 and 0.05, respectively, for 1, 2, and 3 mpf vs. MIA-Control; *Z* = 3.15, 4.85, and 3.51, *p* < 0.05, 0.01, and 0.05, respectively, for 7, 14, and 21 dpf vs. MIA-PBS), but not for PIVE fish. **(D,E)** Tracking of individual fish in a group of four fish shows impaired social cohesion in MIA fish. The movement of each group of fish was analyzed after video tracking. The positions of individual fish in 3 s periods at two different time windows (3 and 7 min, respectively) were traced, and their paths were presented in different colors (#1 fish in red, #2 fish in yellow, and so on). Aggregation of MIA-control/PBS and PIVE-E3 fish groups in a corner of tank is apparent, in comparison with MIA fish. Data are presented as mean ± SEM (right side of the histogram), median ± 75% confidence interval (left side of the histogram), and maximum and minimum. ^*^ *p* < 0.05, ^**^ *p* < 0.001; ^#^ *p* < 0.05, ^##^ *p* < 0.001 compared with the MIA-control group of the same age with social stimulus; ^$^ *p* < 0.05, ^$$^ *p* < 0.001 compared with the MIA-PBS group of the same age with social stimulus; ^%^ *p* < 0.05， ^%%^ *p* < 0.001 compared with the MIA-20 μg/g group of the same age; ^@^ *p* < 0.05， ^@@^ *p* < 0.001 compared with the MIA-50 μg/g group of the same age.^†^ *p* < 0.05, ^††^ *p* < 0.001 compared with the PIVE-E3 group of the same age with social stimulus. ^§§^ *p* < 0.001 compared with the PIVE-10 μM group of the same age; ^£^ *p* < 0.05 compared with the PIVE-50 μM group of the same age. Detail about the *n* and descriptive data for each group and statistical analysis results could be found in Additional file 11: File S4.

The shoaling behavior was estimated with/without acclimation with slight modifications (Kim O. H. et al., 2017; Dwivedi et al., 2019). For larvae, four fish were released into the center of a transparent disk, and their behaviors were immediately recorded for 10 min (without acclimation) and for another 10 min (with acclimation). For juvenile and adult zebrafish, four fish were released in the center of the transparent tank, and their behaviors were immediately recorded for 10 min (without acclimation) and for another 10 min (with acclimation). A camera was fixed to the top of the swimming tanks. The distances between the four fish were estimated every 30 s (Figure 3Ab).

#### 2.4.3. Inattentive behavior test

Inattentive behavior test was done as previously reported (Dwivedi et al., 2019) to examine the response of larvae to an aversive stimulus, as well as their cognition. A moving red bar projected using a PowerPoint slide was used as an aversive stimulus. In brief, a plate was kept at the center on a digital display, 10 larvae were put into each lane for acclimation (30 min) on a blank white background, and followed by aversive stimulation (30 min) with a moving red bar on the lower half of the plate (Additional file 2: Figure S3A). The number of larvae in the upper half of plate (i.e., avoiding the lower half) was counted after every 2 min. Inattentive attitude was quantified by normalizing the number of aversive stimulus period over acclimatization period using the following formula:


$$Larvaeinupper\;half\;overacclimatization\;\left(\%\right)=\frac{Aversivestimulus-Acclimatization}{Acclimatization}\times 100\%$$

#### 2.4.4. OFT

OFT utilizes the innate avoidance of a fish to novel open space to measure anxiety. An OFT was performed using a previously described method with slight modifications (Zakaria et al., 2021), by designing a tank that was virtually divided into a peripheral area and a central area (6 × 4 mm) (Figure 5Ab). For testing, a single fish was introduced into the central area for 1 min and video-tracked for 10 min. The time spent in the central zone was evaluated.

**Figure 4:**
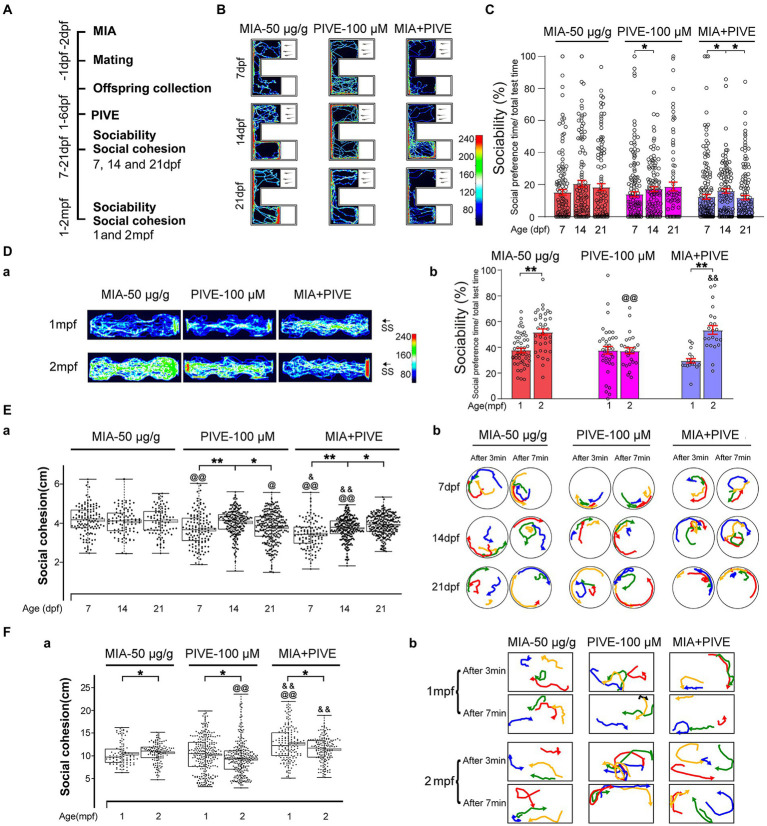
MIA combined with PIVE impaired social approach and decreased social cohesion. **(A)** Zebrafish were tested for sociability and social cohesion at 7, 14, and 21 dpf, and 1 and 2 mpf after MIA + PIVE. **(B)** Video tracking of movements of MIA + PIVE larvae at 7, 14, and 21 dpf, showing the social interaction with the social cue. **(C)** Sociability of MIA + PIVE larvae at 7, 14, and 21 dpf. MIA + PIVE did not further impair the sociability at 7, 14, and 21 dpf. **(Da)** Video tracking of movements of MIA + PIVE fish at 1 and 2 mpf, showing the social interaction with the social cue. **(Db)** Sociability of MIA + PIVE fish at 1 and 2 mpf. Although the sociability in the MIA + PIVE group decreased at 1 mpf, but not statistically significant. **(Ea,Fa)** MIA + PIVE induced an increased social cohesion at 7 and14 dpf (*Z* = 4.73, 4.32, and 3.16, *p* < 0.01, 0.01 and 0.05, respectively, for 7, 14, and 21 dpf vs. MIA-50 μg/g; *Z* = 2.55 and 5.55, *p* < 0.05 and 0.01, respectively, for 7 and 14 dpf vs. PIVE-100 μM), and a decreased social cohesion at 1 and 2 mpf (*Z* = 4.61, *p* < 0.01 for 1 mpf vs. MIA-50 μg/g; *Z* = 5.40 and 6.89, *p* all <0.01 for 1 and 2 mpf vs. PIVE-100 μM). **(Eb,Fb)** Tracking of individual fish in a group of four fish show impaired social cohesion in MIA + PIVE fish. Data are presented as mean ± SEM. ^*^ *p* < 0.05, ^**^ *p* < 0.001; ^@^ *p* < 0.05， ^@@^ *p* < 0.001 compared with the MIA-50 μg/g group of the same age. ^&^ *p* < 0.05, ^&&^ *p* < 0.001 compared with the PIVE-100 μM group of the same age. Detail about the *n* and descriptive data for each group and statistical analysis results could be found in Additional file 11: File S4.

**Figure 5:**
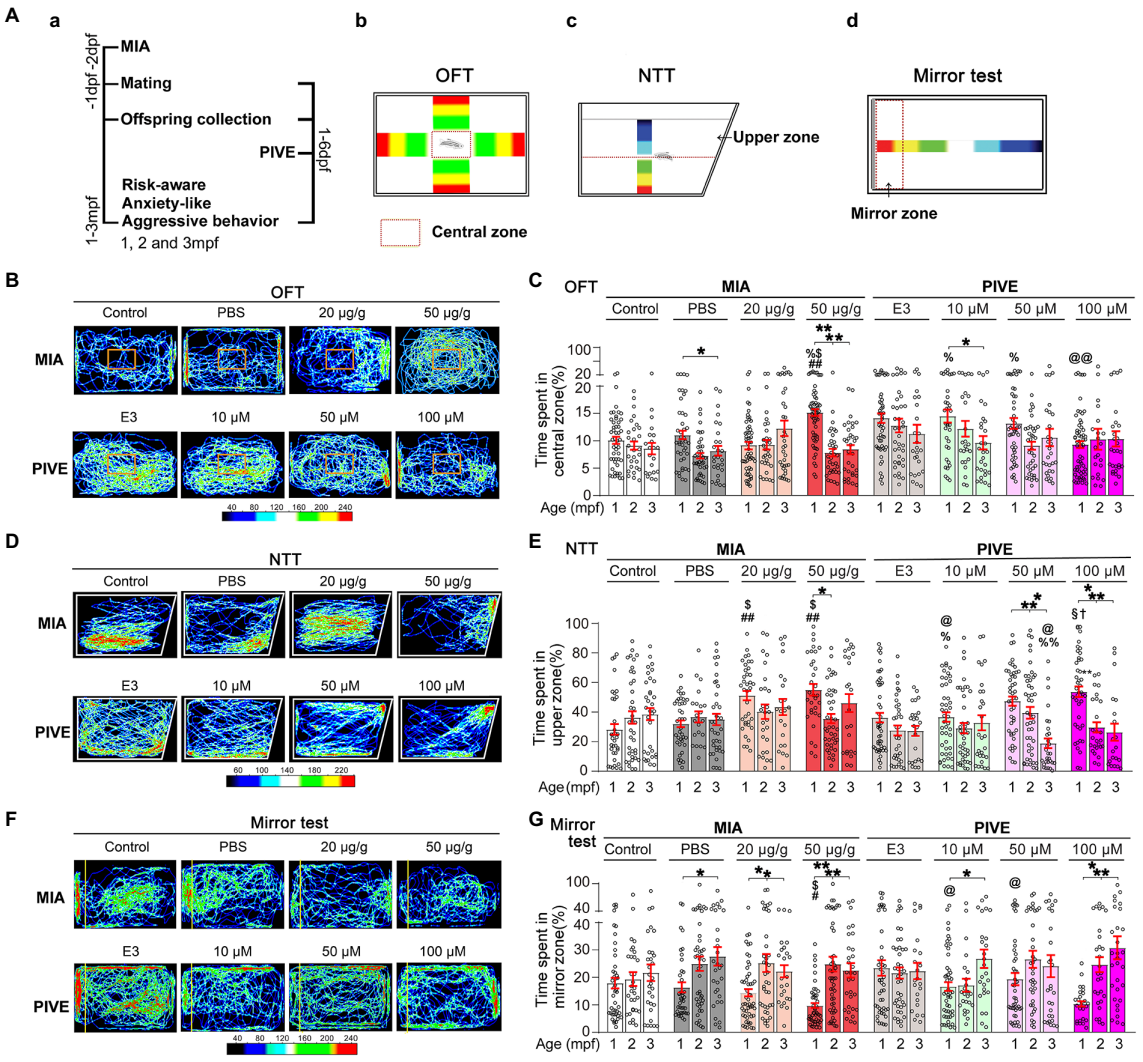
MIA offspring exhibited a decreased risk-aware, anxiety-like and aggressive behavior. **(Aa)** OFT **(Ab)**, NNT **(Ac)**, and mirror **(Ad)** tests were used to evaluate the risk-awareness, anxiety-like and aggressive behavior at 1, 2, and 3 mpf of MIA or PIVE fish. **(B)** Video tracking of movements of MIA or PIVE fish during OFT. **(C)** Only MIA-50 μg/g exhibited a decreased risk-awareness at 1 mpf (MIA-50 μg/g: *Z* = 4.41 and 3.48, *p* < 0.01 and 0.05 vs. MIA-Control and MIA-PBS respectively), as they spent more time in the central zone of the tank. **(D)** Video tracking of movements of MIA or PIVE fish during NNT. **(E)** MIA-20 and 50 μg/g exhibited a decreased anxiety-like behavior at 1 mpf as they spent more time in upper zone of the tank (MIA-20 μg/g: *Z* = 4.17 and 3.46, *p* < 0.01 and < 0.05 vs. MIA-Control and MIA-PBS, respectively. MIA-50 μg/g: *Z* = 4.45 and 3.77, *p* < 0.01 and < 0.05 vs. MIA-Control and MIA-PBS, respectively); as well as observed at 1 mpf in PIVE-100 μM group (*Z* = 3.21, *p* < 0.05 vs. PIVE-E3 group). **(F)** Video tracking of movements of MIA or PIVE fish during mirror test. **(G)** MIA-50 μg/g exhibited a decreased aggressive behavior at 1 mpf as they spent less time in mirror zone of the tank during mirror test (*Z* = 3.17 and 2.96, *p* all <0.05 vs. MIA-Control and MIA-PBS respectively); similar change was not observed in PIVE-100 μM group. Data are presented as mean ± SEM. ^*^ *p* < 0.05, ^**^ *p* < 0.001; ^#^ *p* < 0.05, ^##^ *p* < 0.001 compared with the MIA-control group of the same age with social stimulus; ^$^ *p* < 0.05 compared with the MIA-PBS group of the same age with social stimulus; ^%^ *p* < 0.05， ^%%^ *p* < 0.001 compared with the MIA-20 μg/g group of the same age; ^@^ *p* < 0.05， ^@@^ *p* < 0.001 compared with the MIA-50 μg/g group of the same age.^†^ *p* < 0.05 compared with the PIVE-E3 group of the same age with social stimulus. ^§^ *p* < 0.05 compared with the PIVE-10 μM group of the same age. Detail about the *n* and descriptive data for each group and statistical analysis results could be found in Additional file 11: File S4.

#### 2.4.5. NTT

In this test, a fish was placed into a novel experimental tank for acclimation (10 min) and recorded for 12 min (Audira et al., 2018). The time spent in the upper zone was recorded (Figure 5Ac).

#### 2.4.6. Mirror test

The test was conducted according to a previous study (Vaz et al., 2019), with slight modifications. A tank with a mirror placed on one side was used. A fish was placed at the center of the tank for 6 min. The behavior of the fish was recorded for 10 min using a digital video camera positioned directly above the tank. The area within 3 cm of the mirror was defined as the approach zone. The time spent in the approach zone was recorded (Figure 5Ad).

### 2.5. Library construction and high-throughput sequencing

Total RNA was isolated from brain tissue of zebrafish (1 mpf; MIA: PBS and 50 μg/g groups, PIVE: E3 and 100 μM groups; *n* = 15; about 60 mg of brain tissue; three replicates each group) by the TRIzol (Dingguo, China) according to the manual instruction. The total RNA was qualified and quantified using a Nano Drop and Agilent 2100 Bioanalyzer (Thermo Fisher Scientific, United States). RNA-Seq sequencing library was prepared using TruSeq^™^ RNA sample preparation kit (lllumina, United States). Briefly, purified mRNA was fragmented, then first-strand cDNA was generated using random hexamer-primed reverse transcription, followed by a second-strand cDNA synthesis and end repairing. The cDNA fragments were amplified by PCR to gain double stranded PCR products, which were then heated denatured and circularized by splint oligo sequence to get single strand circle DNA (ssCir DNA), the final library. The final library was amplified with phi29 to make DNA nanoball (DNB), which had more than 300 copies. The DNBs were loaded into a patterned nanoarray and single end 50 bases reads were generated on a BGIseq500 platform (BGI-Shenzhen, China).

### 2.6. Sequencing data processing, and gene ontology (GO) enrichment and Kyoto Encyclopedia of Genes and Genomes (KEGG) analysis of DEGs

**Figure 6:**
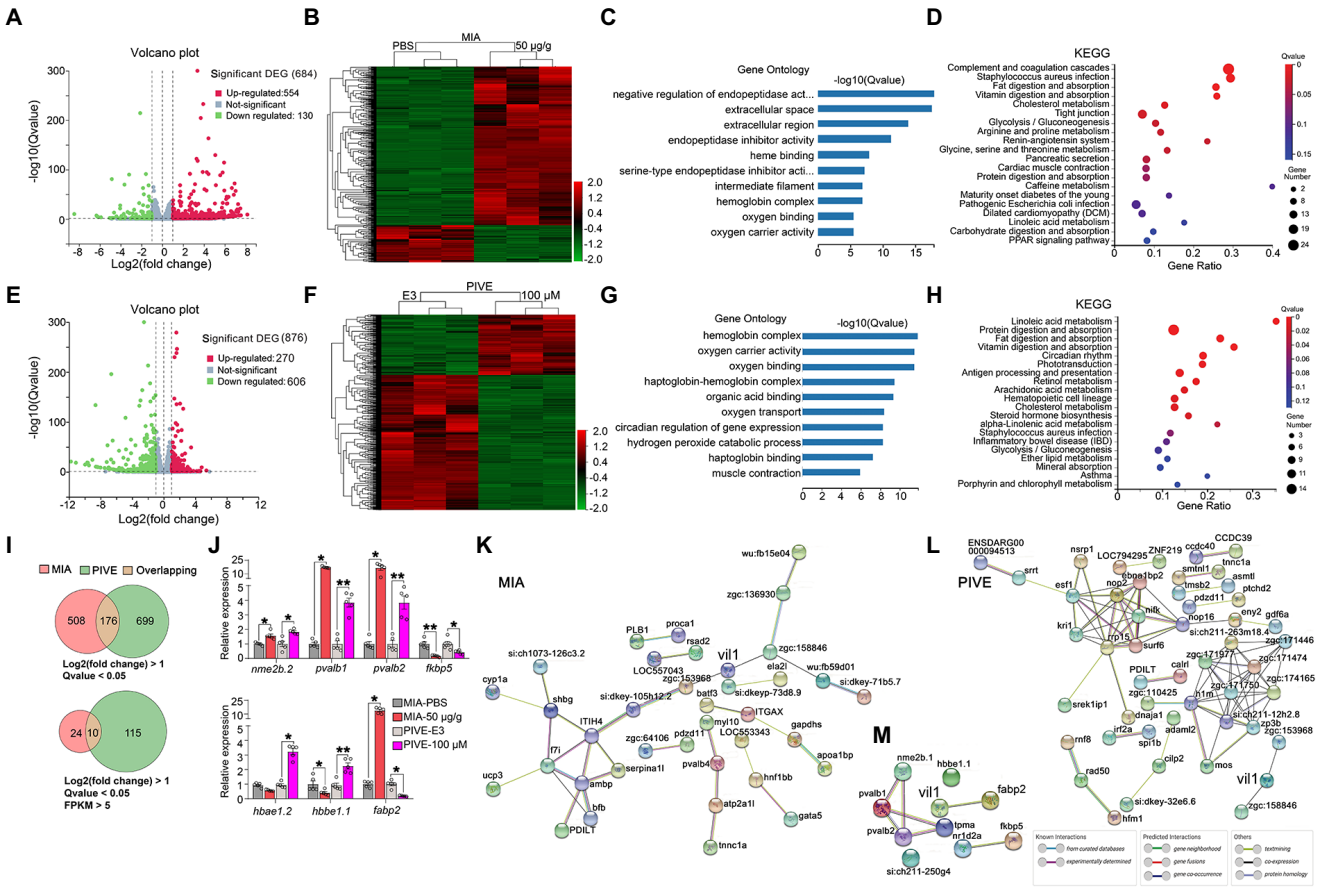
Clustering, GO enrichment and KEGG signaling pathway analysis of DEGs. **(A)** A volcano graph shows DEGs in MIA-50 μg/g zebrafish brain. The red dots indicate up-regulated genes and the green dots indicate down-regulated genes. **(B)** The overall distribution of DEGs between the MIA-50 μg/g group and MIA-PBS group at 1 mpf. Red and green represent up-regulated and down-regulated changes, respectively, in the clustering analysis. The color intensity is directly proportional to the levels of changes. **(C)** GO analysis of MIA-induced DEGs; bar plot shows the top 10 enrichment score [−log10 (*Q*-value)] of DEGs involving biological process, cellular component, and molecular function. **(D)** Significant changes in the KEGG pathway of MIA-induced DEGs. The bubble graph shows enrichment score [−log10 (*Q*-value)] of the significant pathway. The size of the circle represents the number of enriched DEGs. *Q*-value is represented by a color scale, and the statistical significance increases from blue (relatively lower significance) to red (relatively higher significance). **(E)** A volcano graph shows DEGs in PIVE-100 μM zebrafish brain. **(F)** The overall distribution of DEGs in the PIVE-100 μM group and PIVE-E3 group at 1 mpf. **(G)** A GO analysis of PIVE-induced DEGs. **(H)** Significant changes in the KEGG pathway of MIA-induced DEGs. **(I)** Venn diagram denoting the number of all DEGs by two thresholds that are affected in MIA or PIVE offspring. **(J)** Quantitative RT-PCR validation of top 7 co-expressed differential gens in brain of MIA-50 μg/g and PIVE-100 μM group at 1 mpf [MIA-50 μg/g: *t* = 4.33, 25.00(U), 25.00(U), 1.78, 2.50 and 25.00(U), *p* < 0.05, 0.05, 0.05, 0.01, 0.05, and 0.05 for *nme2b.2*, *pvalb1*, *pvalb2*, *fkbp5*, *hbbe1.1*, and *fabp2*, respectively, vs. MIA-PBS. PIVE-100 μM: *t* = 4.18, 7.63, 5.62, 3.04, 25.00(U), 5.13 and 25.00(U), *p* < 0.05, 0.01, 0.01, 0.05, 0.05, 0.01, and 0.05 for *nme2b.2*, *pvalb1*, *pvalb2*, *fkbp5*, *hbbe1.2*, *hbbe1.1*, and *fabp2*, *respectively*, vs. PIVE-E3.]. PPI network of DEGs of MIA-50 μg/g group **(K)** and PIVE-100 μM group **(L)**. **(M)** PPI network of *fabp2*. Nodes represent genes, lines represent the interaction of proteins with genes, and the results within the nodes represent the structure of proteins. Line colors represent evidence of interactions between proteins. Data are presented as mean ± SEM. ^*^ *p* < 0.05, ^**^ *p* < 0.001. Detail about the *n* and descriptive data for each group and statistical analysis results could be found in Additional file 11: File S4.

The sequencing data was filtered with SOAPnuke (v1.5.2)^1^ by removing reads containing sequencing adapter, reads whose low-quality base ratio (base quality less than or equal to 5) is more than 20%, and reads whose unknown base (“N” base) ratio is more than 5%. Then, the clean reads were mapped to the reference genome using HISAT2 (v2.0.4).^2^ Bowtie2 (v2.2.5)^3^ was applied to align the clean reads to the reference coding gene set, then expression level of gene was calculated by RSEM (v1.2.12).^4^ The heatmap was drawn by pheatmap (v1.0.8)^5^ according to the gene expression in different groups. Essentially, differential expression analysis was performed using the DESeq2 (v1.4.5)^6^ with Q value ≤0.05. To learn more about the biological role of these DEGs, GO^7^ and KEGG^8^ enrichment analysis of annotated DEGs was performed by Phyper^9^ based on Hypergeometric test. The significant levels of terms and pathways were corrected by *Q* value with a rigorous threshold (*Q* value ≤0.05) (Bonferroni correction for multiple comparisons). The STRING database^10^ was used to infer PPIs in DEGs’ enrichment pathways (Szklarczyk et al., 2017) (Figure 6).

### 2.7. Validation of selected genes by RT-qPCR

**Figure 7:**
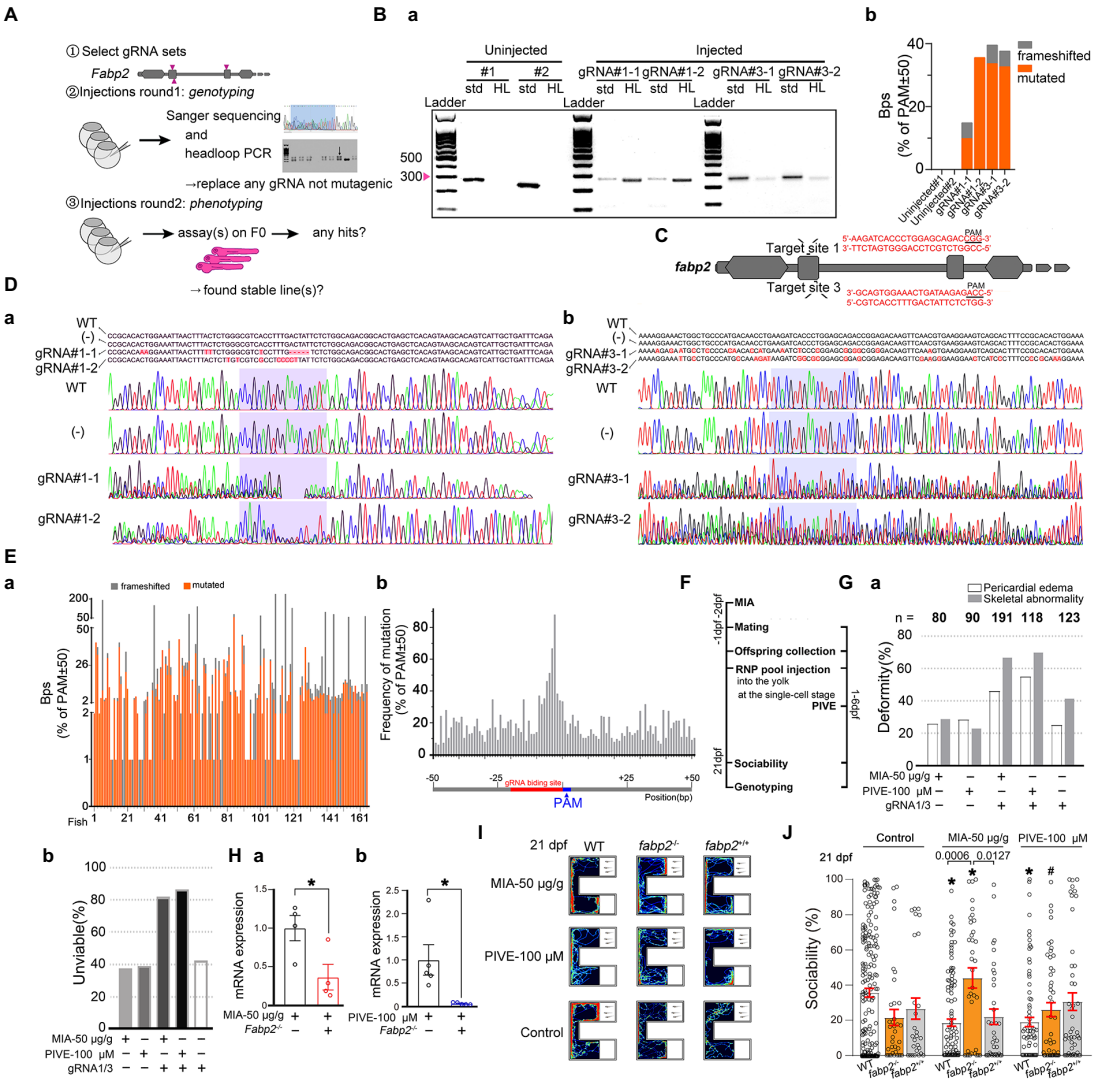
*Fabp2* gene F0 knockout ameliorated MIA-induced autism-like behaviors. **(A)** Two rounds F0 knockout of *fabp2* gene including selection of gRNAs, verification of mutagenic gRNAs and phenotyping. **(Ba)** Target loci of *fabp2* amplified with the PCR primes used for sequencing (std, standard) or with headloop primer (HL). **(Bb)** Frameshifted and mutated bps (Sanger sequencing) of the same samples as showed in **(Ba)**. **(C)** Schematic diagram of the target site in the zebrafish *fabp2* genome. **(Dab)** Sequencing maps of WT and *fabp2^−/−^* zebrafish. (−): gRNA not mutagenic. **(Ea)** Frameshifted and mutated bps (Sanger sequencing) of all samples (83 fish at 21 dpf; 46 fish at 1 mpf which for finding stable lines). **(Eb)** Frequency of mutations of R50-PAM-F50 (83 fish at 21 dpf; 46 fish at 1 mpf which for finding stable lines). **(F)** After maternal poly(I:C) injection (MIA), two-RNP pool was injected into the yolk at the single-cell stage before cell inflation; or two-RNP pool was injected into the yolk at the single-cell stage followed by PIVE to these embryos, and zebrafish were tested for sociability at 21 dpf followed by verification of genotyping. **(Ga)** Deformity of gRNA injection with MIA or PIVE. **(Gb)** Unviability of gRNA injection with MIA or PIVE. **(Hab)** mRNA expressions of *fabp2* with MIA or PIVE (MIA: *t* = 2.72, *p* < 0.05; PIVE: *U* = 0.00, *p* < 0.05). **(I)** Video tracking of movements of WT or *fabp2^−/−^* larvae with MIA or PIVE at 21 dpf. **(J)** Sociability of WT or *fabp2^−/−^* larvae with MIA or PIVE at 21 dpf. *Fabp2* F0 knockout ameliorated MIA-induced autism-like behaviors (*fabp2^−/−^* + MIA: Z = 4.06, *p* < 0.01 vs. *fabp2^−/−^* without MIA). Data are presented as mean ± SEM. ^*^ *p* < 0.05 compared with Control group of the same zebrafish line. ^#^ *p* < 0.05 compared with MIA-50 μg/g group of the same zebrafish line. Detail about the *n* and descriptive data for each group and statistical analysis results could be found in Additional file 11: File S4.

The most differential co-expressed genes of two groups (MIA-50 μg/g and PIVE-100 μM) were selected and detected by quantitative RT-PCR. At 1 mpf, 5–6 fish were collected from the MIA-PBS, MIA-50 μg/g, PIVE-E3 and PIVE-100 μM groups, and total RNA was extracted by TRIZOL reagent (Dingguo). Reverse transcription was performed with PrimeScriptTM RT Reagent Kit (RR036A, TaKaRa, Japan), according to the manufacturer’s protocol. RT-qPCR was performed using a StepOnePlusTM apparatus (ABI, United States) and TB Green Premix Ex TaqTM (RR420A, TaKaRa, Japan). The thermal cycle was as follows: pre-denaturation at 95°C for 30 s, then 40 cycles at 95°C for 5 s, 60°C for 15 s, and 60°C extension for 30 s. The relative gene expression was normalized to an endogenous housekeeping gene (β-actin) and the formula 2^−△△Ct^ was calculated using the comparative Ct method (three replicates for each sample) (Figures 6J, 7Hab). The primer sequences used in this study are listed in Additional file 4: File S3.

### 2.8. F0 knockout of *fabp2* and screens

F0 knockout of *fabp2* gene in zebrafish was carried out as previously reported, using an approach including two rounds of embryo injections: a validation round followed by a phenotyping round (Selvaraj et al., 2019; Figure 7A).

#### 2.8.1. Cas9/gRNA preparation

Synthetic gRNA consisting of two components was bought from Integrated DNA Technologies (IDT, United States): the crRNA (Alt-R CRISPR-Cas9 crRNA) and tracrRNA (Alt-R CRISPR-Cas9 tracrRNA, #1072532). The *fabp2* crRNA was predesigned by IDT (more information sees Additional file 4: File S3).^11^ The crRNA was annealed with the tracrRNA to form the gRNA by mixing each crRNA of the set separately with an equal molar amount of tracrRNA and diluting to 57 μM in Duplex buffer (IDT, #11–01–03-01). For gRNA/Cas9 assembly, Cas9 protein (IDT, Alt-R S.p. Cas9 Nuclease V3, 61 μM, #1072533) was diluted to 57 μM in Cas9 buffer: 20 mM Tris–HCl, 600 mM KCl, 20% glycerol, and then equal volumes of gRNA and Cas9 solutions were mixed (typically 1 μl gRNA; 1 μl Cas9), incubated at 37°C for 5 min then cooled on ice, generating a 28.5 μM RNP solution. The three RNP solutions were pooled in equal amounts before injections. The concentration of each RNP was thus divided by three (9.5 μM each), leaving the total RNP concentration at 28.5 μM. Approximately 1 nl of the three-RNP pool was injected into the yolk at the single-cell stage before cell inflation. This amounts to around 10.0 fmol of RNP (10.0 fmol [1,646 pg] of Cas9 and 10.0 fmol [333 pg] of total gRNA). Each unique RNP was present in equal amounts in the pool. Therefore, in the case of three RNPs, 3.3 fmol of each RNP was co-injected. Three *scrambled* crRNAs (Alt-R CRISPR-Cas9 Negative Control crRNA #1, #2, #3) were prepared into RNPs and injected following the same steps as above (more information sees Additional file 4: File S3).

#### 2.8.2. Unviability

The percentage of unviable embryos/larvae was evaluated as previously reported (Selvaraj et al., 2019), based on the total number of embryos/larvae that died or were dysmorphic (not associated with the expected phenotype) after 1 dpf. This death/dysmorphic count was divided by the total number of larvae at 1 dpf to get a percentage of unviable embryos/larvae. Percentages of unviable embryos/larvae in the uninjected or scrambled controls were usually low (<9%) (Figure 7Gab).

#### 2.8.3. Genomic DNA extraction

After individual larvae were transferred to 0.2 ml PCR tubes separately, excess liquid was removed from each tube and 50 μl of 1× base solution (25 mM KOH, 0.2 mM EDTA in water) was added. The tubes were incubated at 95°C for 30 min, then cooled to room temperature before the addition of 50 μl of 1× neutralization solution (40 mM Tris–HCL in water). Genomic DNA was stored at 4°C.

#### 2.8.4. PCR and sanger sequencing

Each PCR well contained: 2 μl 5× Phusion HF buffer, 0.2 μl dNTP (10 mM), 0.1 μl Phusion High-Fidelity DNA Polymerase, 1.0 μl genomic DNA, 0.05 μl forward primer (10 μM), 0.05 μl reverse primer (10 μM), and 6.6 μl dH_2_O; for a total of 10.0 μl. The PCR plate was sealed and placed into a thermocycler. The PCR program was: 95°C for 5 min, then 35 cycles of: 95°C for 20 s, 70°C for 20 s, 72°C for 20 s, then 72°C for 7 min then cooled to 4°C until collection. PCR primers are provided in Additional file 4: File S3. The concentration of PCR product was quantified by Qubit, and the length of the product was verified on 2.5% agarose gel. The samples were sent to Tsingke Biotechnology Co., Ltd. (Hangzhou, China) for Sanger sequencing. Briefly, the PCR products were purified and secondary verified on a 1% agarose gel. The BigDye^®^ Terminator v3.1 (Thermo Fisher Scientific) and 3,730 sequencer was used for sequencing.

#### 2.8.5. Headloop PCR

Headloop PCR was done as previously described (Rand et al., 2005; Selvaraj et al., 2019). Assessment of PCR products on agarose gel is sufficient to determine whether the target loci were effective mutants in F0 embryos. The headloop PCR primer pairs and the headloop tags are provided in Additional file 4: File S3. For headloop PCR, each well contained: 2 μl 5× Phusion HF buffer, 0.2 μl dNTPs (10 mM), 0.05 μl forward primer (10 μM), 0.05 μl reverse primer (10 μM), 0.1 μl Phusion Hot Start II polymerase, 1 μl genomic DNA, 6.6 μl dH_2_O; for a total of 10.0 μl. PCR program was: 98°C for 30 s; then 30 cycles of: 98°C for 10 s, 72°C for 20 s; then 72°C for 7 min. Amplification was assessed by agarose gel electrophoresis (Figure 7Da). To calculate the headloop PCR score, GelDoc Go gel imaging system (Bio-Rad) was used to imaging the gel. Then, Quantity One software (Bio-Rad) was used to quantify band intensity.

#### 2.8.6. F0 screens in two rounds of injections

In the first round, each *fabp2* gRNA was injected separately, followed by headloop PCR to confirm mutagenesis, thereby controlling the false negative rate of a screen. If a gRNA was found not to generate enough mutations, it was replaced with a new one and the experiment was repeated; or directly abandoned. The second round of injection used mixed *fabp2* gRNA sets to generate the F0 knockouts for phenotyping. The control larvae were injected with a set of scrambled RNPs, which controlled for any potential effect caused by the injection of Cas9 and exogenous RNA.

### 2.9. Statistical analysis

We used ImageJ software to analyze the behavior tracking data. SPSS 23.0 (IBM, Armonk, NY, United States) were used for statistical analysis. All continuous data conformed to the normal distribution (according to the Kolmogorov–Smirnov test) were analyzed using One/Two-way ANOVA followed by Šídák’sor Tukey’s multiple comparisons test under the condition of a significant *F* value (*p* < 0.05). Those data with non-normal distribution were analyzed using Kruskal-Wallis test followed by Bonferroni’s multiple comparisons test; or using Multiple Mann–Whitney tests with Bonferroni’s correction under the condition of a significant *H* value (*p* < 0.05). Prior to testing, we had performed several pilot experiments identifying the best concepts for our experimental paradigms. To obtain convincing statistical power, we included all samples produced from several batches/crosses for MIA and PIVE paradigm only discarding fishes with obvious deformities or immobility within the test time. Since we did not find any batch/cross effects on our behavior results in statistical tests, no multiple testing corrections were made. Data were expressed as mean ± standard error of the mean (M ± SEM). *p* < 0.05 were considered as statistically significant.

## 3. Results

### 3.1. MIA offspring born to mothers injected with poly(I:C) showed an immune response

In rodents, exposing to MIA (Patterson, 2011) serves as an environmental risk factors-ASD model. At 24 h after maternal poly(I:C) injection, TLR4ba mRNA expressions were significantly down-regulated in intestinal tissue and liver of maternal fish (Figure 1Ca), which can be seen in TLR3 and TLR4bb, but there was no statistical significance. TLR3 and TLR4bb, but not TLR4ba, mRNA expressions were up-regulated in spleen of maternal fish (Figures 1Ba,Ca,Da), which was not statistically significant when compared to those injected with PBS. Compared with the expressions in spleen, TLR3, TLR4ba and TLR4bb mRNA expressions were significantly down-regulated in intestinal tissue of maternal fish (Figures 1Ba,Ca,Da). Considering zebrafish do not have a placenta and are external fertilizers, it is unknown whether mothers injected with poly(I:C) would also induce an immune reaction in MIA offspring. We found that TLR3 (*p* = 0.0667) and TLR4bb mRNA expressions were significantly up-regulated but that of TLR4ba of MIA offspring at 1 dpf was not (Figures 1Bb,Cb,Db). For PIVE, there were no significant changes in TLR3 and TLR4ba/b mRNA expressions of the offspring collected at 1 dpf after a 24 h poly(I:C) treatment (Figures 1Bb,Cb,Db); while both TLR3 and TLR4ba/b mRNA expressions of the offspring collected at 2 dpf after a 24 h poly(I:C) treatment were significantly up-regulated (Figures 1Bb,Cb,Db). Furthermore, we measured the immune response parameters after the poly(I:C) treatment. We found that the levels of IgM were increased in MIA-50 μg/g offspring at 14 dpf (*p* = 0.0835, compared to those injected with PBS). Although the levels of C4 were increased in MIA-20 μg/g and MIA-50 μg/g offspring at 21 dpf the change was not significant when compared with the controls (Figures 1E,F). Interestingly, the levels of IgM, C4 and IgA were significantly increased in PIVE-100 μm larvae only at 21 dpf (Figures 1E–G). At 24 h after poly(I:C) injection, the levels of IgM, C4 and IgA were increased in maternal liver, which were not statistically significant, and it could not be found in brain, spleen and intestinal tissues (Figures 1Ha,Hb,Hc).

### 3.2. MIA offspring born to mothers injected with poly(I:C) exhibited impaired social approach

The offspring produced by pregnant rodents with MIA have abnormal behavior that is reminiscent of autism, which makes MIA a useful disease model (Smith et al., 2007). However, whether this type of autism phenotype can be replicated in zebrafish has not been reported. Normally, zebrafish showed a development of social approach behavior from 7 to 21 dpf (Figures 2B,C). The levels of sociability (as a ratio of either total test time or total interaction time) were significantly lower in MIA-50 μg/g group at 21 dpf compared to the MIA-Control and MIA-PBS group, respectively, (Figures 2B,C; Additional file 2: Figure S2A; Additional file 5: Movie S1). We examined whether directly exposing embryos to poly(I:C) could also impair the social approach behavior and found that PIVE-10, 50, and 100 μM did not impair the sociability (as a ratio of either total test time or total interaction time) at 7 and 14 dpf (Figures 2B,C and Additional file 2: Figure S2A). Only PIVE-100 μM impaired the sociability (as a ratio of total test time) when being compared with the PIVE-E3 group (*p* = 0.0662) at 21 dpf (Figures 2B,C; Additional file 6: Movie S2).

Sociability was also tested for these fish at 1, 2, and 3 mpf. Only MIA-50 μg/g offspring exhibited impaired social approach behavior at 1 mpf (Figures 2D,E; Additional file 2: Figure S2B; Additional file 7: Movie S3). PIVE-100 μM also impaired social approach behavior at 2 mpf (Figures 2D,E; Additional file 2: Figure S2B; Additional file 8: Movie S4). Poly(I:C) exposure also induced changes of total distance moved and average velocity during the social preference test of larvae (Additional file 2: Figure S4) and juvenile/adult zebrafish (Additional file 2: Figure S5). We did not find any sex differences at 3 mpf of these poly(I:C) exposure-induced sociability and movement changes (Additional file 2: Figure S6).

### 3.3. MIA offspring born to mothers injected with poly(I:C) exhibited had decreased social cohesion

We next tested group behavior of the poly(I:C) treated fish using the shoaling assay. The distances between individual fish can indicate their social interaction or impaired social behavior among conspecifics. Zebrafish larvae showed a development of social cohesion from 7 to 21 dpf (Figures 3B,D) and remained stable from 1 to 3 mpf (Figures 3C,E). Comparing MIA and control/PBS fish revealed that MIA groups had significantly larger mean distances between individual fish at 7, 14, and 21 dpf, and 1, 2, and 3 mpf (Figures 3B,D). We plotted the path of individual fish after video tracking (Figures 3D,E). Social cohesion, aggregation, or shoaling behaviors were apparent in the control/PBS fish groups. However, individual fish of the MIA groups moved independently from one another, suggesting a deficit in social interaction (Additional file 9–110: Movies S5, S6). In contrast, this pattern of activity was not detected in PIVE groups (Figures 3B–E). Poly(I:C) exposure also induced changes of total distance moved and average velocity during social cohesion test in zebrafish (Additional file 2: Figure S7).

### 3.4. MIA offspring born to mothers injected with poly(I:C) combined with PIVE exhibited impaired social approach and decreased social cohesion

Since our results found that either MIA or PIVE at least partially impaired social approach and decreased social cohesion, we further tested the effects of MIA combined with PIVE on social approach and cohesion. In the MIA-50 μg/g + PIVE-100 μM group, the offspring did not exhibit impaired social approach behavior during the social stimulating test at 7, 14, and 21 dpf when compared to that of MIA-50 μg/g and PIVE-100 μm groups, respectively, (Figures 4A,B,C). Although the sociability in the MIA-50 μg/g + PIVE-100 μM group decreased (Figures 4B,C), similar to that observed at 1 mpf compared to that of MIA-50 μg/g group (Figures 4Da,Db), but not statistically significant. This group recovered to the normal levels and was significantly higher than that of PIVE-100 μM group at 2 mpf (Figures 4Da,Db; Additional file 2: Figure S2C). For social cohesion, the MIA + PIVE group had significantly shorter mean distances between individual fish at 7 and 14 dpf than the MIA-50 μg/g or PIVE-100 μM group (Figures 4Ea,Eb). However, the MIA + PIVE group did have markedly longer mean distance between individual fish at 1 mpf than the MIA-50 μg/g group (Figures 4Fa,Fb).

### 3.5. MIA offspring born to mothers injected with poly(I:C) exhibited a decreased risk-aware, anxiety-like and aggressive behavior

ASD children often have less risk awareness. Therefore, we use OFT to evaluate risk-awareness of poly(I:C)-treated zebrafish. We found that only MIA-50 μg/g group exhibited a decreased risk-awareness at 1 mpf (Figures 5B,C), as they spent more time in the central zone of the tank. Conversely, PIVE did not affect this behavior (Figures 5B,C). Similarly, MIA-20 and 50 μg/g groups exhibited a decreased anxiety-like behavior at 1 mpf (Figures 5D,E) as they spent more time in upper zone of the tank during NNT; similar changes were observed in PIVE-100 μM group at 1 mpf (Figures 5D,E). We also found that MIA-50 μg/g group exhibited a decreased aggressive behavior at 1 mpf (Figures 5F,G) as they spent less time in mirror zone of the tank during the mirror test; a similar change was observed in PIVE-100 μM group at 1 mpf, which was not statistically significant when compared to the PIVE-E3 group (Figures 5F,G). To determine whether this decreased awareness of risk and new environment and decreased social interaction in fish with poly(I:C) exposure is due to impaired cognition, we used an inattentive behavior test to check their cognition of “aversive” stimulus. We found that only MIA-50 μg/g group did exhibit a decreased cognition at 14 dpf, as the larvae of MIA-50 μg/g were less responsive to “aversive” stimulus and stayed longer in “aversive” stimulus area. Such changes were not observed at 7 and 21 dpf (Additional file 2: Figure S3B). The larvae of PIVE-100 μM did not show a reduced response to “aversive” stimulus compared with PIVE-E3 group at 7, 14, and 21 dpf (Additional file 2: Figure S3B). Therefore, the decreased awareness of risk, new environment and social interaction in fish (1 to 3 mpf) with poly(I:C) exposure might not result from poly(I:C) induced cognition impairment.

### 3.6. Transcriptomic characterization of the brains of MIA fish

Given the striking behavioral abnormalities after poly(I:C) exposure, we performed a transcriptomic survey of the brain of 1 mpf fish (MIA: PBS and 50 μg/g group, PIVE: E3 and 100 μM group; *n* = 15). RNA-sequencing analysis of brain RNA from the MIA group identified 684 DEGs (threshold: *p* < 0.05; Log_2_ (Fold Change) > 1.0 or Log_2_ (Fold Change) < −1.0; Additional file 4: File S3), which are summarized in a volcano plot (Figure 6A). The volcano plot illustrates that the numbers of overexpressed genes were significantly more than that of underexpressed genes (up vs. down: 554 vs. 130 genes). Moreover, a hierarchical clustering of DEGs was conducted (Figure 6B). To gain an insight into the biology of the expression changes observed in the MIA brain, we performed a GO enrichment analysis and a KEGG pathway annotation. These analyses were performed to identify GO enrichment in the categories of cellular components, biological processes, and molecular functions. For GO enrichment, the DEGs were plentiful in negative regulation of endopeptidase activity, extracellular space, extracellular region, endopeptidase inhibitor activity, heme binding, etc. (Figure 6C). The KEGG pathway found that DEGs were mainly enriched in complement and coagulation cascades, fat digestion and absorption, cholesterol metabolism, and tight junction, etc. (Figure 6D). For PIVE, RNA-sequencing analysis of brain RNA identified 876 DEGs (threshold: *p* < 0.05; Log_2_ (Fold Change) > 1.0 or Log_2_ (Fold Change) < −1.0), which are summarized in a volcano plot (Figure 6E). The volcano plot also illustrates that the numbers of underexpressed genes were significantly more than that of overexpressed genes (under vs. over: 606 vs. 270 genes). A hierarchical clustering of DEGs was conducted (Figure 6F). For GO enrichment, the DEGs were plentiful in hemoglobin complex, oxygen carrier activity, oxygen binding, haptoglobin-hemoglobin complex, and organic acid binding (Figure 6G). The KEGG pathway found that DEGs were mainly enriched in the linoleic acid metabolism, protein digestion and absorption, fat digestion and absorption, phototransduction, antigen processing and presentation (Figure 6H).

The top 10 co-expressed differential genes of two groups (MIA-50 μg/g and PIVE-100 μM) were selected and detected by RT-qPCR. Testing on one gene (*hbae1.3*) was not made due to its homology to *hbae1.2*, and two genes (*tpma* and *nr1d2a*) failed in our experiment. The results showed that expressions of the remaining top 7 co-expressed differential genes were similar to that of RNA sequencing (Figures 6I,J and Additional file 2: Figure S8).

To determine the interaction between DEGs related to social behavior deficits in GO enrichment and KEGG signaling pathways, we identified a potential PPI network for these DEGs (Figure 6K for MIA; Figure 6L for PIVE). The PPI network integrated these DEGs using STRING analysis; both PPI enrichment *p*-value was statistically significant (*p* < 0.001). From the PPI network, we found that *vil1* forms a more complicated network with other genes in MIA than in PIVE. We speculated that *vil1* mediated pathways might play an important role in the MIA-induced ASD in zebrafish. Furthermore, using STRING analysis involves the top 10 co-expressed differential genes of the MIA-50 μg/g and PIVE-100 μM groups and *vil1,* we found only *fabp2* interacted with *vil1* (Figure 6M), exactly representing that MIA has the tendency of overexpression and PIVE has the tendency of underexpression.

Next, we searched on SFARI Gene^12^, an evolving online database designed to permit tracking the ever-expanding genetic risk factors that emerge in the literature https://www.sfari.org/resource/sfari-gene/ – bottom for ASD, and got a total of 991 ASD scored genes. We analyzed the RNA-sequencing results for these genes. The MIA group identified 10 DEGs (threshold: *p* < 0.05; Log2 (Fold Change) > 1.0 or Log2 (Fold Change) < −1.0), which are summarized in a volcano plot (Additional file 2: Figure S9A). Moreover, a hierarchical clustering of DEGs was conducted (Additional file 2: Figure S9B). The GO enrichment analysis and a KEGG pathway annotation were performed. For GO enrichment, the DEGs were plentiful in fibrinogen complex, semaphoring receptor complex, lamellipodium, etc. (Additional file 2: Figure S9C). The KEGG pathway found that DEGs were mainly enriched in complement and coagulation cascades, tryptophan metabolism, cGMP-PKG signaling pathway, etc. (Additional file 2: Figure S9D). For PIVE, RNA-sequencing identified 18 DEGs (threshold: *p* < 0.05; Log2 (Fold Change) > 1.0 or Log2 (Fold Change) < −1.0), which are summarized in a volcano plot (Additional file 2: Figure S9E). A hierarchical clustering of DEGs was conducted (Additional file 2: Figure S9F). For GO enrichment, the DEGs were plentiful in RNA polymerase II transcription factor complex, FACT complex, host cell nucleus, etc. (Additional file 2: Figure S9G). The KEGG pathway found that DEGs were mainly enriched in circadian rhythm, endocrine and other factor-regulated calcium reabsorption, mineral absorption, etc. (Additional file 2: Figure S9H). From the PPI network, we found that only *vil1-fabp2* and *atp1a1a.4* co-incidenced in both MIA and PIVE (Additional file 2: Figures S9J,K).

### 3.7. *Fabp2* gene F0 knockout ameliorated MIA-induced autism-like behaviors

In the first round, three gRNAs were injected separately followed by headloop PCR, and we found that two gRNAs generated enough mutations. In the second round of injections, we used two confirmed *fabp2* gRNA sets to generate F0 knockouts for phenotyping (Figures 7A,Bab,C). The rates of deformity and unviability were high with *fabp2* gRNA1/3 set injections (Additional file 2: Figures S10B,C). All headloop PCR results were identical with that of Sanger sequencing. The ratios of mutated and frameshifted events were 89.09% (147/165) and 70.30% (116/165) of the total occurrence, respectively. The mutated and frameshifted base pairs (bps) were 43.20% (1,389/3,215) and 56.80% (1,826/3,215) of the total variation numbers (bps) respectively (Figures 7Da,Db,Ea). Frequency of mutations of R50-PAM-F50 did not show any distribution patterns (Figure 7Eb). We found that the sociability in *fabp2^−/−^* zebrafish was not significantly impaired at 21 dpf when compared to WT (Figures 7F,I,J). However, *fabp2^−/−^* rescued the sociability of MIA-induced social behavior deficits (Figures 7I,J), as the sociability was enhanced significantly in *fabp2^−/−^* zebrafish treated with MIA. (Additional file 2: Figure S2D); *fabp2^−/−^* also ameliorated the sociability of PIVE-induced social behavior deficits but without a statistical significance compared to the *fabp2^−/−^*-Control group (Figures 7I,J).

## 4. Discussion

Although several environmental risk factor-ASD models such as the MIA-induced model have been established in rodent, so far there have been no reported MIA-induced ASD model with behavioral features in zebrafish. In this study, we established a MIA-induced ASD zebrafish model by demonstrating the involvement of immune activations and characterizing its ASD-like phenotypes.

Studies on rodent models have shown that MIA is sufficient to cause ASD independently, with the offspring showing abnormal brain morphology as well as ASD-like phenotypes (Shi et al., 2003; Shin Yim et al., 2017; Garcia-Valtanen et al., 2020; Haddad et al., 2020). Since zebrafish do not have a placenta and are external fertilizers that do not have induced maternal immune response to affect developing fetus during pregnancy, establishing an MIA-induced ASD zebrafish model is relatively hard. Previous studies have established that TLRs detect exogenous and endogenous threats through pathogen-associated molecular patterns (PAMPs) and damage-associated molecular patterns (DAMPs) and then activate the innate immune system to produce pro-inflammatory cytokines (Han et al., 2021). TLRs are expressed on peripheral immune cells and CNS cells, including microglia and neurons. Classic rodent animal models of MIA use the PAMPs poly(I:C) to stimulate TLR3 and trigger a maternal inflammatory response (Meyer, 2014). DAMPs, such as self RNA, self DNA, high mobility group protein B1 (HMGB1) and heat shock proteins, are normal cell constituents that are released from endogenous damaged cells, which stimulate TLR4 to trigger a maternal inflammatory response (Akira and Takeda, 2004; Tang et al., 2012). Here, we showed that poly(I:C) exposure activated the maternal innate immune system not only by TLR3 but also by TLR4. Furthermore, TLR3 and TLR4bb mRNA expressions were significantly up-regulated in MIA offspring as well as the levels of IgM/C4 were increased in MIA offspring (Figures 1Bb,Cb,Db); while there were no significant changes in TLR3 and TLR4ba/b mRNA expressions in PIVE offspring after a 24 h poly(I:C) exposure (Figures 1Bb,Cb,Db). It suggested that the effects of one-dose maternal poly(I:C) injection is stronger than that of 24 h direct poly(I:C) exposure on eggs, indicating that maternal poly(I:C) exposure did activate innate immune system of their offspring and these effects on the eggs in the MIA model were likely a result of MIA but not a direct effect of poly I:C on the eggs penetrating the placenta. The proposed immune mechanisms of transmitting the effects of MIA to the developing fetus include dysregulated maternal innate, adaptive and complement pathways, and maternal autoantibodies (Knuesel et al., 2014). In rodent animal models, an underlying mechanism of MIA-induced behavioral abnormalities in offspring may involve an imbalance of pro-and anti-inflammatory cytokines in the maternal-placental-fetal axis (Horváth et al., 2019; Lammert and Lukens, 2019; Garcia-Valtanen et al., 2020; Haddad et al., 2020; Reed et al., 2020; Jaini et al., 2021). RNA-sequencing analysis of brain cytokine RNA from the MIA group identified 1 DEG (ccl19b) (Additional file: Figures S12A,B), indicating potential roles of cytokines in mediating the effects of MIA on the developing offspring. Testing cytokine expressions in early developing offspring might get more interesting results. Comparing with MIA, the increased numbers of DEGs of brain cytokine RNA from RNA-sequencing analysis suggest that PIVE larvae have a stronger immune activation with upregulated TLR expressions and increased levels of complement and autoantibodies. We proposed that MIA offspring did not have a continuous effect of the maternal immune response on the developing larvae. Our results indicated that poly(I:C) exposure (both MIA and PIVE) activated the innate immune system through both PAMPs and DAMPs.

ASD is characterized by altered social communication (Lai et al., 2014). In this study, we found that if zebrafish mothers were exposed to poly(I:C) before mating, their offspring exhibited social impairments, especially in social cohesion, as indicated by the significantly larger mean distance between individual fish during the whole study periods (Figures 3B,D). A tendency of moving independently from one another of the fish suggests a likely deficit in social interaction (Additional file 9–10: Movies S5, S6). A comparison among maternal exposure, *in vitro* exposure to embryos, and maternal exposure combined with *in vitro* exposure to embryos (Additional file 2: Figure S11) reveals obviously differences in the degree of social behavioral deficits. For example, the MIA-50 μg/g offspring showed impaired sociability at early larvae stage, decreased social cohesion during whole study age periods, and decreased risk-aware, anxiety-like and aggressive behavior; but PIVE-100 μM zebrafish did not exhibit the same pattern (Meshalkina et al., 2018). Our results indicated that the social behavior tests in this study were sensitive and that different exposures led to different phenotypes. Consistently, PIVE group with a stronger immune activation had a more severely impaired social approach and social cohesion. MIA + PIVE group also had a more severely impaired social approach and social cohesion than MIA or PIVE, but not in social cohesion at 7, 14, and 21 dpf. In comparisons with control/PBS group, the social cohesion of MIA + PIVE group impaired gradually from 7 to 21 dpf as the distance between individuals was lowest at 7 dpf, and highest at 21 dpf; while the distance between individuals of control/PBS group gradually decreased from 7 to 21 dpf. Why did the social cohesion of MIA + PIVE group show inconsistent results? The reason is unknown. We speculate that part of the reason may be related to the weakening of juvenile activities and the reduction of their moving distance caused by MIA + PIVE treatment, thus masking the impact on social cohesion. With the continuous growth, the side effects of this treatment gradually subside. A meta-analysis of 15 studies found that common maternal bacterial infections during pregnancy increased the odds of offspring ASD by 13% (Jiang et al., 2016). The present results provided evidences that immune activation was directly correlated with the phenotypic variety/severity of ASD (Ashwood and Wakefield, 2006; Atladóttir et al., 2010).

Poly(I:C) is a synthetic dsRNA that is recognized by TLR3 and can activate host immune defense. Systemic administration of poly(I:C) induces viral-like acute inflammatory response (Tsukada et al., 2021). Our results as mentioned above indicated that maternal poly(I:C) exposure activated the innate immune system in zebrafish through not only TLR3 but also TLR4.The presence of behavioral anomalies and immune activations of poly(I:C)-exposed offspring (both MIA and PIVE) provides a unique opportunity for identifying molecular correlates of resilience and susceptibility to poly(I:C) exposure [28]. Therefore, we performed RNA sequencing to compare genome-wide transcriptional changes in these poly(I:C)-exposed offspring. Our GO and KEGG analyses of RNA-sequencing results showed that the pathways of DEGs were different between MIA and PIVE-offspring brain tissues. The KEGG analysis showed that MIA-offspring brain tissues were plentiful in complement and coagulation cascades. Similar results were observed in those with poly(I:C) exposure to zebrafish embryos except complement and coagulation cascades. We found overexpression of complement genes in MIA brain tissues, including *si:dkey-105 h12.2*, *si:dkey-32n7.4*, *f7*, cfb, *c3a.1*, *serpinc1*, *plg*, *f2*, *f5*, *fgb*, *fga*, *proca*, *c8g*, *fgg*, *c8a*, *kng1*, *c3a.6*, *cfh*, *c9*, *serpinf2a*, *serpinf2b*, *c3a.3*, *c5*, and *serping1.* However, the expression levels of these genes in the brain tissues in PIVE-100 μM group did not change or even decreased. As observed in this study, different immune alterations are associated with different phenotypes, which is consistent with the existence of various characteristics or subgroups of human ASD phenotypes (Ashwood and Wakefield, 2006; Atladóttir et al., 2010). Meanwhile, the results indicate that complement activation played an important role in MIA-induced ASD. Accumulating evidence suggests that the pathogenesis of ASD involves a dysregulated complement pathway (Fagan et al., 2017), which includes increased frequencies of C4B alleles in ASD patients and their mothers (Warren et al., 1991), increased levels of C1q and C3 and C3 fragments in the plasma of ASD children (Corbett et al., 2007; Momeni et al., 2012), and hyper-activation of the complement system in postmortem brain tissue from ASD patients (Hutsler and Zhang, 2010; Tang et al., 2014). The abnormal complement signaling as a result of inflammatory insult during pre-and postnatal development may lead to alterations of cerebral connectivity resulting from diminished complement-mediated synaptic pruning, and may contribute to ASD pathophysiology (Schafer et al., 2012). According to previous observation in rodent MIA-model, the top identified canonical signaling pathways range from altered neuronal signaling pathways such as dopamine-and cAMP-regulated phosphoprotein 32 kDa (DARPP-32) signaling, γ-aminobutyric acid receptor signaling, and opioid signaling, to mitochondrial oxidative phosphorylation and translation initiation by eukaryotic initiation factor 2 signaling, which was dependent on brain region and markedly differed between subgroups (Mueller et al., 2021). Although we were not able to stratify MIA/PIVE-exposed offspring into resilient and susceptible subgroups by the cluster analysis and to correlate DEGs to these subgroups, the different phenotypes and patterns of DEGs between MIA and PIVE we have found indicate that poly(I:C)-exposed zebrafish was a useful ASD model for studying phenotypes and molecular mechanisms. We further analyzed the RNA-sequencing results for 991 ASD scored genes. From PPI network, we found that only *vil1-fabp2* and *atp1a1a.4* appeared simultaneously in MIA and PIVE groups (Additional file 2: Figures S9J,K), indicating that these genes may be key mediators of poly(I:C) exposure behavior changes from MIA and PIVE in zebrafish. This result is different from what have been observed in rodent MIA models relating multiple cell signaling pathways, such as opioid signaling, G-protein-coupled receptor signaling, CXCR4 signaling, CREB signaling in neurons, MTOR signaling, oxidative phosphorylation, EIF2 signaling, camp-mediated signaling, DARRP-32 signaling, and GABA receptor signaling, which contained more ASD-risk genes in their observed DEGs (Mueller et al., 2021).

The different phenotypes and patterns of DEGs between MIA and PIVE groups and the results of PPI network analysis suggest that *vil1* forms a more complicated network with other genes in MIA than in PIVE. In the top 10 co-expressed differential genes of two groups (MIA-50 μg/g and PIVE-100 μM), we found only *fabp2* interacted with *vil1* (Figure 6M). We demonstrated that MIA-induced social behavioral deficits were ameliorated by *fabp2* knockout. Similar effect was achieved in those treated with PIVE, as the sociability was enhanced significantly although the amplitude was lesser than MIA. A role of *fabp2* in ASD has not been reported previously. Only one previous study found that intestinal fatty acid binding protein (IFABP, FABP2), an index of gastrointestinal permeability, was significantly increased in serum of ASD patients (Saresella et al., 2016). A relevant result showed that the plasma *fabp2* level in patients with anxiety and depression was significantly higher than that in the control group (Stevens et al., 2018). *Fabp2* is a biomarker of barrier integrity of gut epithelium tight junction, which is upregulated and released by the presence of dysbiotic microbiota. Altogether, *fabp2* can be considered as a novel biomarker or target for psychiatric diseases including ASD, anxiety, and depression. On the other hand, *Vil1* is altered following an induction of cell stress in intestinal epithelial cells. Acute changes in actin dynamics increased intestinal epithelial cell survival, whereas long-term changes in actin dynamics lead to intestinal epithelial cell death and intestinal inflammation (Roy et al., 2018). Thus, we proposed that *fabp2*-*vil1* signaling may play a pivotal role in MIA-induced ASD.

There are several studies have generated intestinal *FABP* (*FABP2*) and liver *FABP* (*FABP1*) knockout mice and their phenotypes have been characterized. Both *FABPs* are important in the net intake of dietary lipids, which have unique functions in intestinal lipid assimilation involved in systemic energy metabolism (Gajda et al., 2023). *FABP2*-knockout did not cause death of mice, but their weight would change with a hyperinsulinemia, but *FABP2* was not necessary for dietary fat absorption (Vassileva et al., 2000). Another study results showed that *FABP2*-knockout led to changes in gut motility and morphology, resulting in a relatively lean phenotype at the whole-body level (Lackey et al., 2020). Concluded, *FABP2* may participate in dietary lipid sensing and signal transduction, affect intestinal motility, intestinal structure and nutrient absorption, and thus affect systemic energy metabolism. However, there was no mental disease-like phenotype such as ASD observed in *FABP2*-knockout mice. Zhao et al. (2020) knocking out or overexpressing of *fabp2* in zebrafish and found that *fabp2* could promote intestinal n-3 PUFA absorption to mediate TAG synthesis and CL homeostasis, by regulating the genes involved in lipid metabolism, as well as there was no mental disease-like phenotype could be observed in *fabp2^−/−^* zebrafish. In contrast, we observed that the sociability in *fabp2^−/−^* zebrafish (21 dpf) was impaired (Figures 7I,J), which was not shown in adulthood (data not shown). Furthermore, *fabp2^−/−^* rescued the sociability of MIA/PIVE-induced social behavior deficits. It is unclear how *fabp2* is related to the ASD-like behavior, and how *fabp2 ^−/−^* affects MIA/PIVE-induced social behavior defects in zebrafish. Recently, Wei et al. (2022) carried out a bioinformatics analysis of genomic and immune infiltration patterns in ASD. They used weighted correlation network analysis (WGCNA) to separate 5,000 DEGs into eight significant modules and two hub genes were found (one of was *FABP2*). Immune cell infiltration showed that *FABP2* was significantly associated with memory B cells and CD8 T cells and could affect multiple pathways of immunity. We hypothesize that *fabp2* may influence the immune microenvironment by regulating immune cells and immune-related pathways inducing an ASD behavior as observed in the present study that *fabp2 ^−/−^* larvae showed an impaired sociability. As mentioned above, MIA-offspring brain tissues were plentiful in complement and coagulation cascades, *fabp2*-knockout may also influence the immune microenvironment to rescue the social behavior deficits from the Poly(I:C) exposure. *Fabp2* not only is a candidate molecular marker for the development of ASD, but also influence the ASD behavior in both causing and ameliorating directions.

In addition, the prevalence of ASD in male is generally higher than that in female (4: 1) (Werling and Geschwind, 2013). It may be related to gene difference on X chromosome or sex hormone, epigenetic regulations which may be sex-biased. There are many ASD-related genes on the X chromosome, such as *FRM1*, *NLGN4X* (Nguyen et al., 2020; Wong et al., 2020). However, we did not find any gender difference at 3 mpf of the MIA-induced sociability and movement changes (Additional file 2: Figure S6), indicating that the immune activation involved in the MIA-induced ASD zebrafish model may not lead to phenotypic differences between gender.

## 5. Limitations

The major limitation of the present study is that the results are confined to a single maternal injection of poly(I:C) within 24 h before mating to induce MIA. Particularly, it is not known what the effects of longer-term treatment on behavior would be. Further studies are required to titrate the lowest dose of poly(I:C) for altering social behavior. It remains possible that a combination of different doses, injection time and / or frequency, interval and / or route of administration may result in better model generation with improved phenotype. It remains unknown whether there is any difference between genders in larvae of MIA-induced ASD. It is also not known what the phenotype of *fabp2^−/−^* fish would be and what the longer-term effects of *fabp2* knockout would be on social behavior after poly(I:C) exposures, as we only tested up to 21 dpf. Finally, the underlying molecular and neural mechanisms about how *fabp2* knockout rescues social behavior deficits require further study.

## 6. Conclusion

In this study, we established an environmental risk factor-ASD model in zebrafish and demonstrated its social behavior impairments that mimic human ASD phenotypes. The model replicated the phenotype of human ASD with multiple comorbidities and characteristics. Both maternal exposure and direct embryo exposure of poly(I:C) resulted in activations of the innate immune system through toll-like receptor 3/4. GO and KEGG analysis of RNA sequencing data found that the MIA-induced DEGs were mainly concentrated in complement and coagulation cascade pathways. PPI network analysis of the detected DEGs suggested that *vil1* pathways may play a key role in MIA-induced ASD. *Fabp2,* the only gene in the top 10 DEGs which interacted with the key node (*vil1*) of the concentrated PPI network, was upregulated in MIA offspring but downregulated in PIVE offspring. Knocking out *fabp2* rescued the social behavior deficits in both MIA and PIVE offspring. Overall, our work established an ASD model with assessable behavior phenotype in zebrafish and provided key insights into environmental risk factor and the influence of *fabp2* gene on ASD-like behavior.

## Data availability statement

The original contributions presented in the study are included in the article/Supplementary material, further inquiries can be directed to the corresponding author.

## Ethics statement

The animal study was reviewed and approved by the Animal Care and Use Committee at Zhejiang University School of Medicine (16779).

## Author contributions

JWu, KJ, and XJ designed the experiments. JWu, XL, DW, BY, PZ, MB, JWa, and CY performed the main experiments. JWu, XL, and KJ created the *fabp2* knockout F0 zebrafish. JWu, ZL, and KJ performed the RNA sequencing analysis. JWu and KJ wrote the manuscript with the help of XJ. All authors read and approved the final manuscript.

## Funding

KJ was supported by the National Natural Science Foundation of China (81871012 and 81571263), ZL was supported by the National Natural Science Foundation of China (81901325), and also supported by the Natural Science Foundation of Zhejiang Province (LY20H090017 and LY20H090015).

## Conflict of interest

The authors declare that the research was conducted in the absence of any commercial or financial relationships that could be construed as a potential conflict of interest.

## Publisher’s note

All claims expressed in this article are solely those of the authors and do not necessarily represent those of their affiliated organizations, or those of the publisher, the editors and the reviewers. Any product that may be evaluated in this article, or claim that may be made by its manufacturer, is not guaranteed or endorsed by the publisher.

## Supplementary material

The Supplementary material for this article can be found online at: https://www.frontiersin.org/articles/10.3389/fnmol.2022.1068019/full#supplementary-material

## Footnotes

^1^ https://github.com/BGI-flexlab/SOAPnuke

^2^ http://www.ccb.jhu.edu/software/hisat/index.shtml

^3^ http://bowtiebio.sourceforge.net/%20Bowtie2%20/index.shtml

^4^ https://github.com/deweylab/RSEM

^5^ https://cran.r-project.org/web/packages/pheatmap/index.html

^6^http://www.bioconductor.org/packages/release/bioc/html/ DESeq2.html

^7^ http://www.geneontology.org/

^8^ https://www.kegg.jp/

^9^ https://en.wikipedia.org/wiki/Hypergeometric_distribution

^10^ https://string-db.org/

^11^ eu.idtdna.com

^12^ https://www.sfari.org/resource/sfari-gene

## Abbreviations

ASD: Autism spectrum disorders
DEG: Differential expression genes
DNB: DNA nanoball
dpf: Days post-fertilization
E3: Embryonic media
Fabp2: Fatty acid-binding protein 2
GO: Gene ontology
i.p.: Intraperitoneal injection
IFABP: Intestinal fatty acid binding protein
KEGG: Kyoto Encyclopedia of Genes and Genomes
LC50: Half-maximal lethal concentrations
MIA: Maternal immune activation
mpf: Months post-fertilization
NTT: Novel tank test
OFT: Open field test
PBS: Phosphate buffered solution
PIVE: Polyinosinic:polycytidylic acid in vitro exposure
PPI: Protein–protein interactions
Poly(I:C): Polyinosinic:polycytidylic acid
RT-qPCR: Reverse transcription quantitative PCR
SEM: Standard error of the mean
SS: Social stimulus
TLR: Toll-like receptor
TS: Test subject
Vil1: villin-1
WT: Wild type
