# Supplementary material for: Poly(I:C)-exposed zebrafish shows autism-like behaviors which are ameliorated by fabp2 gene knockout
Source: Front Mol Neurosci. 2023 Jan 5;15:1068019. doi: 10.3389/fnmol.2022.1068019 (PMC9849760; doi:10.3389/fnmol.2022.1068019)
Supplement: Supplementary file 1 [file Data_Sheet_1.DOCX]

**Additional file 1: File S1. The zebrafish husbandry methodology**

**Zebrafish care and husbandry**

All experiments and animal handling were performed according to the Guide for the Care and Use of Laboratory Zebrafish by the China Zebrafish Resource Center and were approved by the Animal Care and Use Committee at Zhejiang University School of Medicine (16779). Wild-type (WT) zebrafish AB strain was housed in a modular zebrafish system (Haisheng, China) as we previously reported(Wu et al., 2022), and all fish were kept in a 10-h dark / 14-h light cycle, and 28 ± 0.5 °C filtered and UV sterilized water. We collected fertilized embryos within 1 h after mating. After the collection, the fertilized embryos ( ≤ 50) were put into Petri dishes (90 mm in diameter) containing embryonic media (E3) (pH = 7.0), and then move to an incubator (28 °C). Change the water in the morning and evening every day. At 5 days post fertilization (dpf), take them out of the incubator and put into a breeding tank (700 ml, n ≤ 50/L). Feed Paramecium at 5 - 7 dpf, Paramecium + #100 at 7 - 15 dpf, three times a day, and change water 20 min after each feeding. After 15 dpf, put them into the circulation system (700 ml, n ≤ 30/L), feed #150 + shrimp at 15 - 30 dpf, and feed adult fish regular food + shrimp after 30 dpf (700 ml, n ≤ 8/L). The fish tank is cleaned once a week. The pH value of circulating water is 7.0 - 8.0; the conductivity is generally about 500 - 800 µS/cm.

**Breeding**

For breeding, after keeping adult female and male fish (female vs male =2:1) separate overnight in a 1-L crossing tank, we released them by removing the divider at 8-9 am the next morning and collected the fertilized embryos within 1 h after their releasing. After being maintained in embryonic media E3 for 24 h (petridish, diameter = 90 mm, 28.5℃, n ≤ 50), healthy embryos were selected and raised regularly.

During zebrafish breeding, catch dead fish, isolate diseased fish, and euthanize old fish in time.

Wu, J., Yan, B., Bao, M., Shen, J., Zheng, P., Wu, D., et al. (2022). Early life exposure to chronic unpredictable stress induces anxiety-like behaviors and increases the excitability of cerebellar neurons in zebrafish. *Behav Brain Res* 437**,** 114160. doi: 10.1016/j.bbr.2022.114160.
