# Supplementary material for: Poly(I:C)-exposed zebrafish shows autism-like behaviors which are ameliorated by fabp2 gene knockout
Source: Front Mol Neurosci. 2023 Jan 5;15:1068019. doi: 10.3389/fnmol.2022.1068019 (PMC9849760; doi:10.3389/fnmol.2022.1068019)

**Additional file 1: Fig.S1-12**

Figure S1


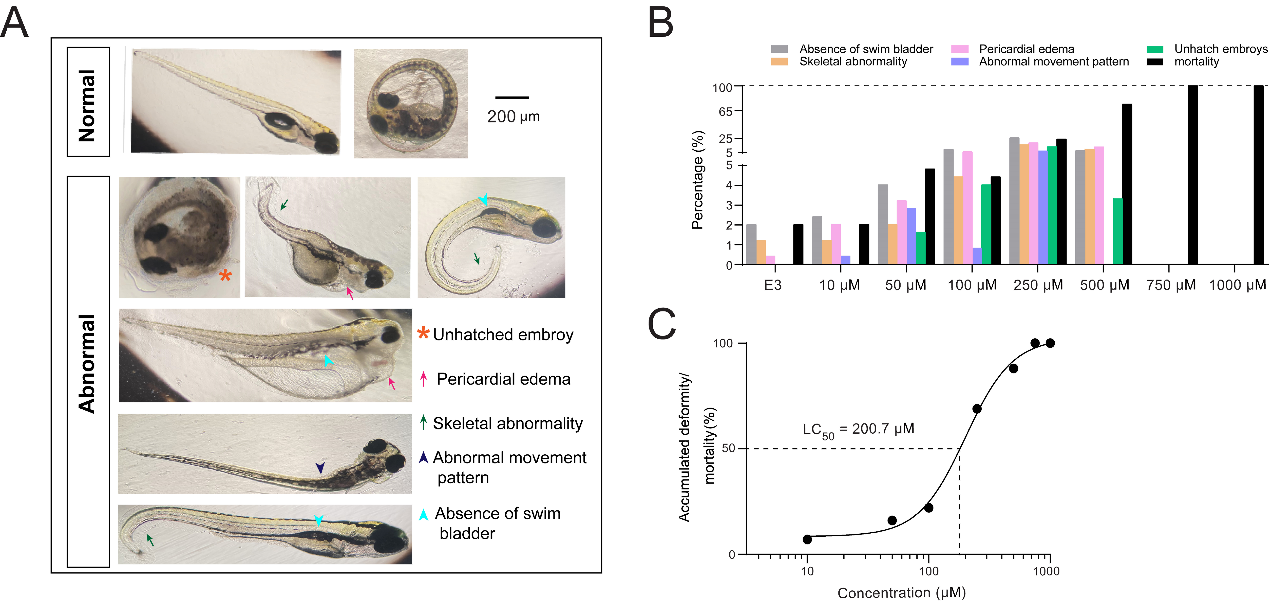


Figure S2


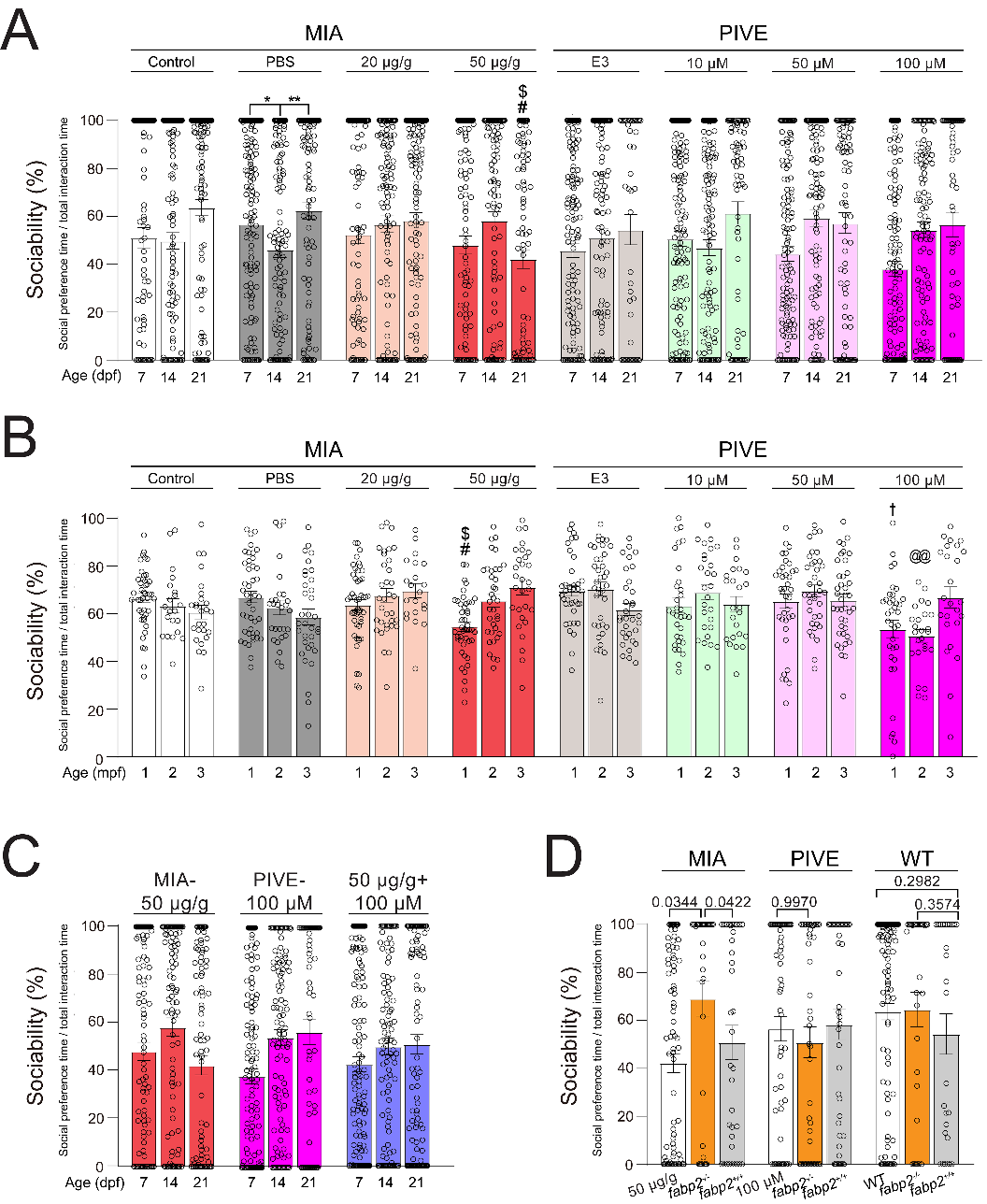


Figure S3


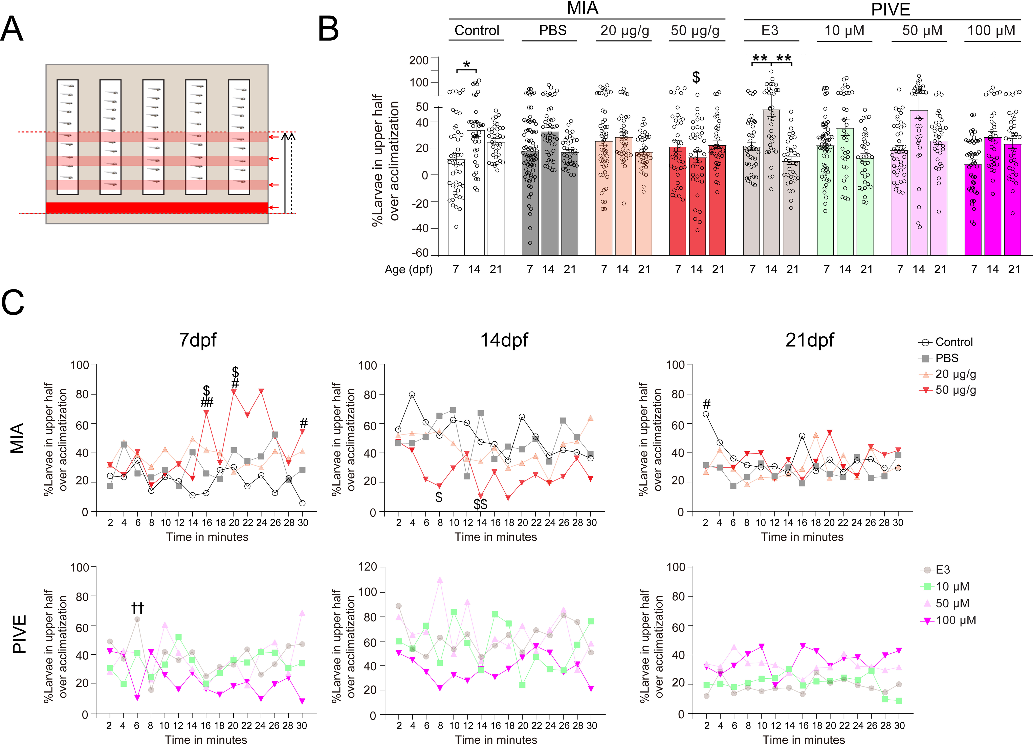


Figure S4


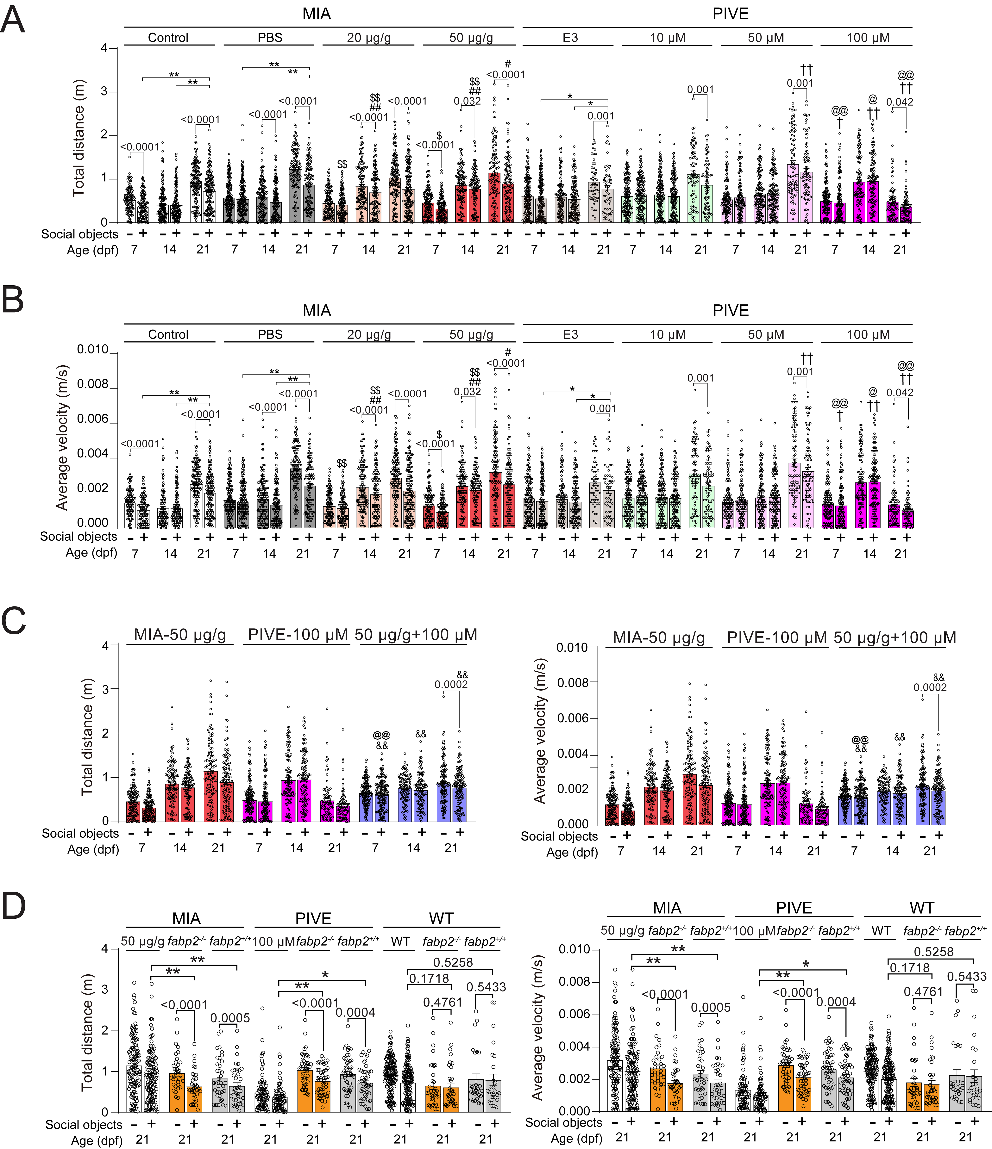


Figure S5


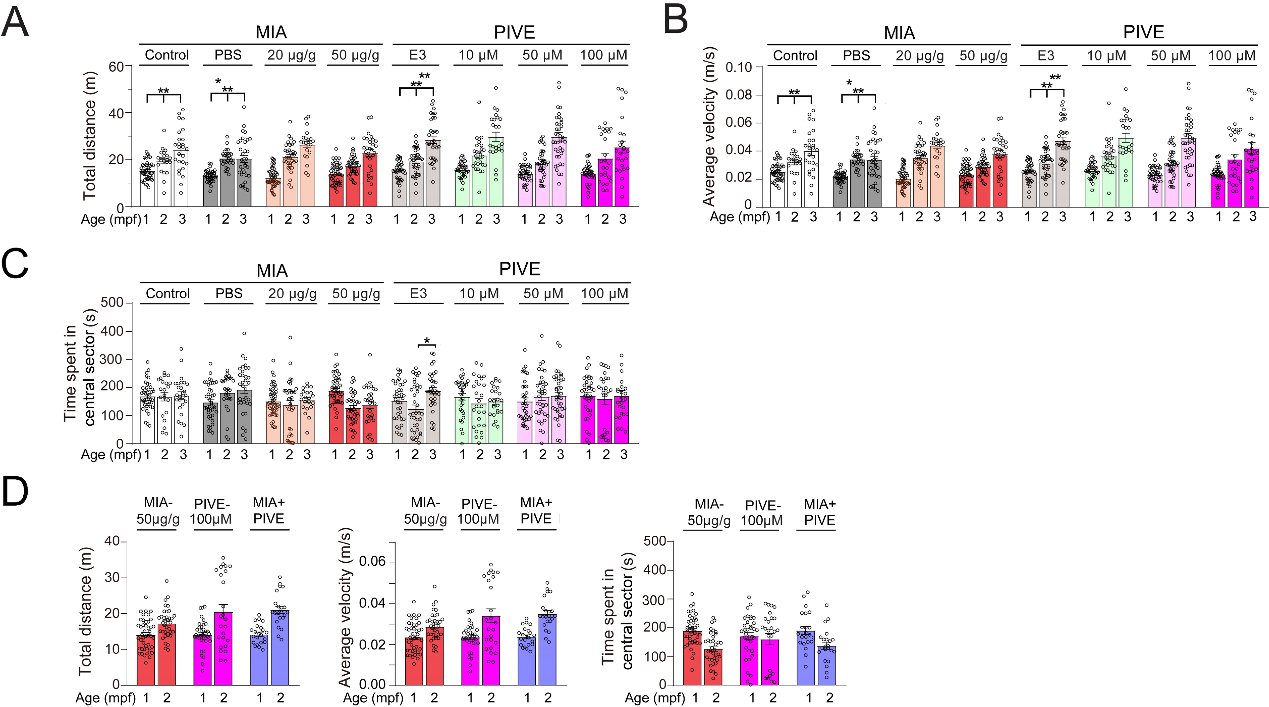


Figure S6


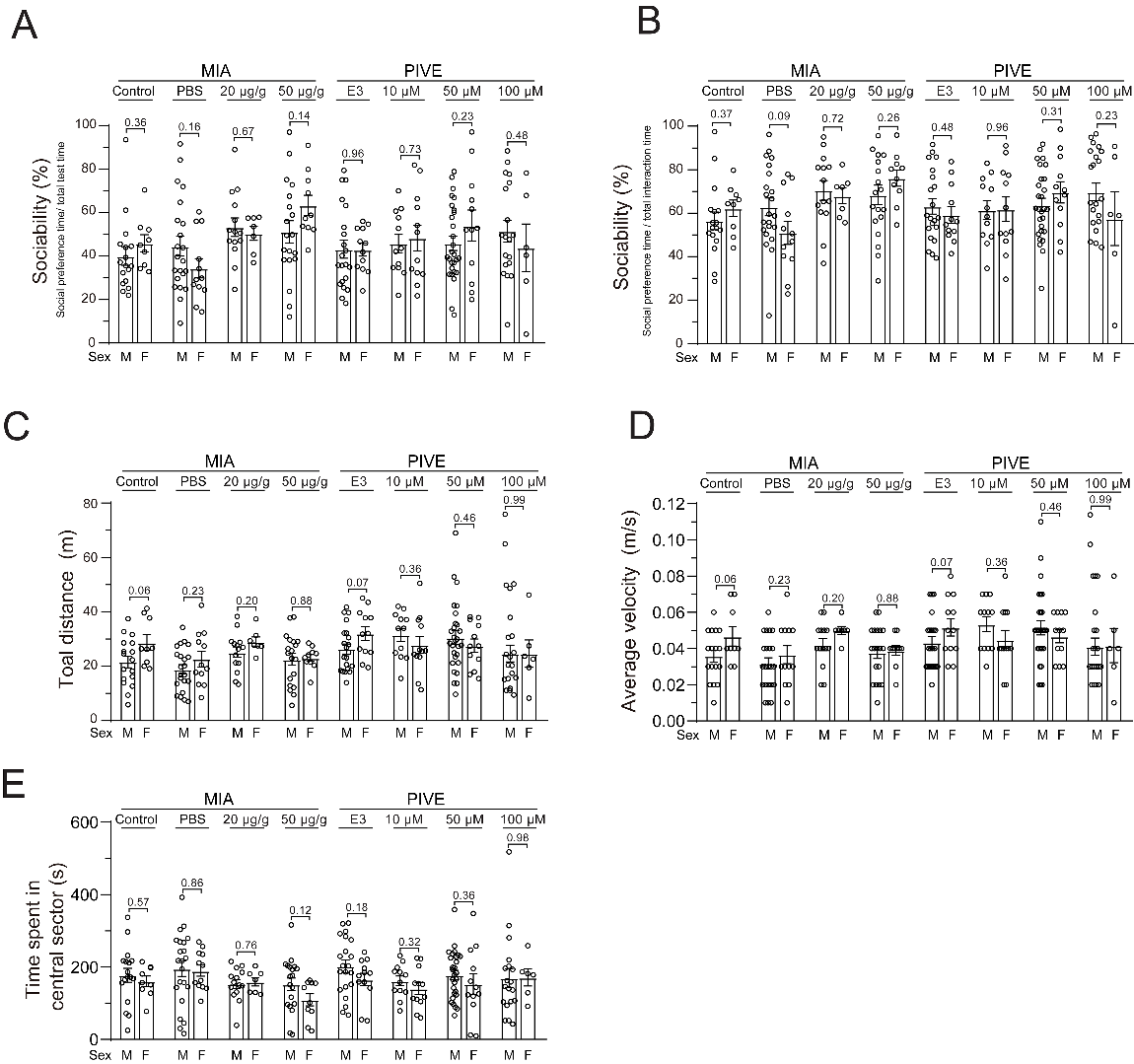


Figure S7


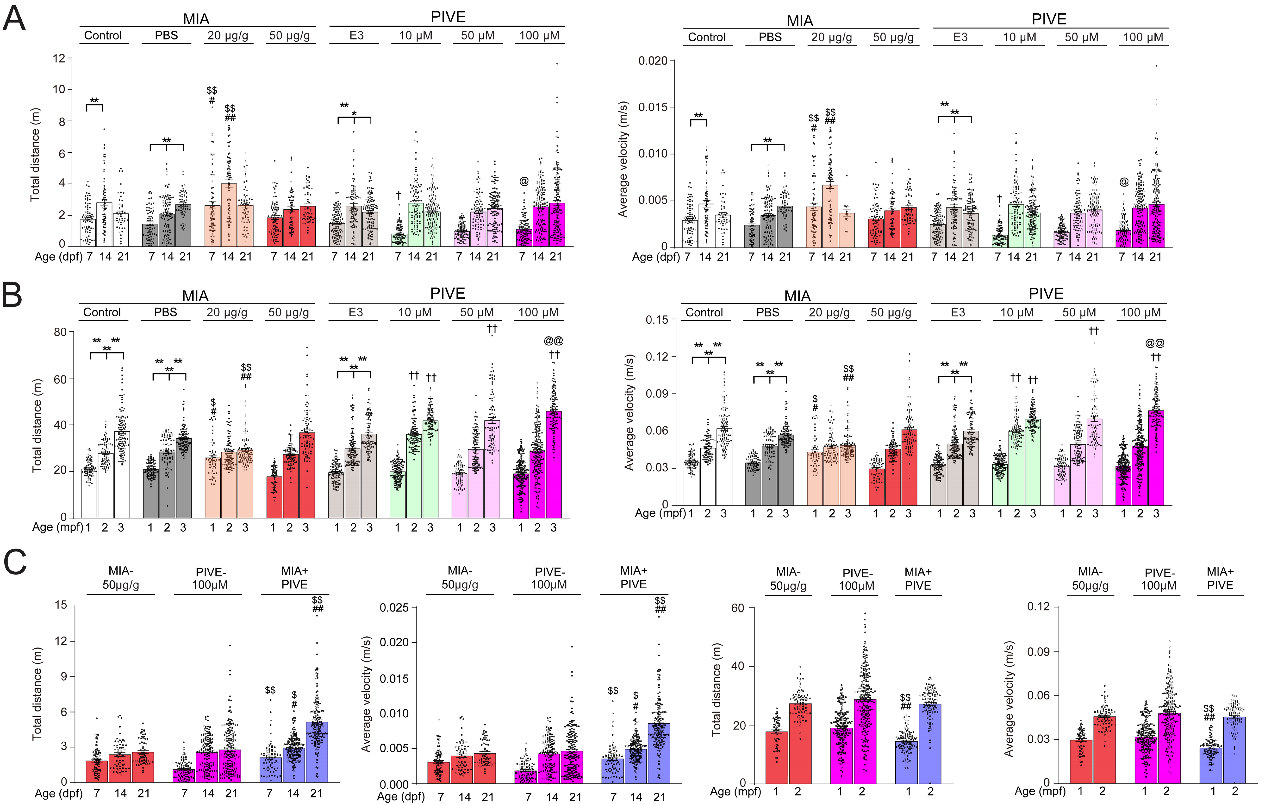


Figure S8


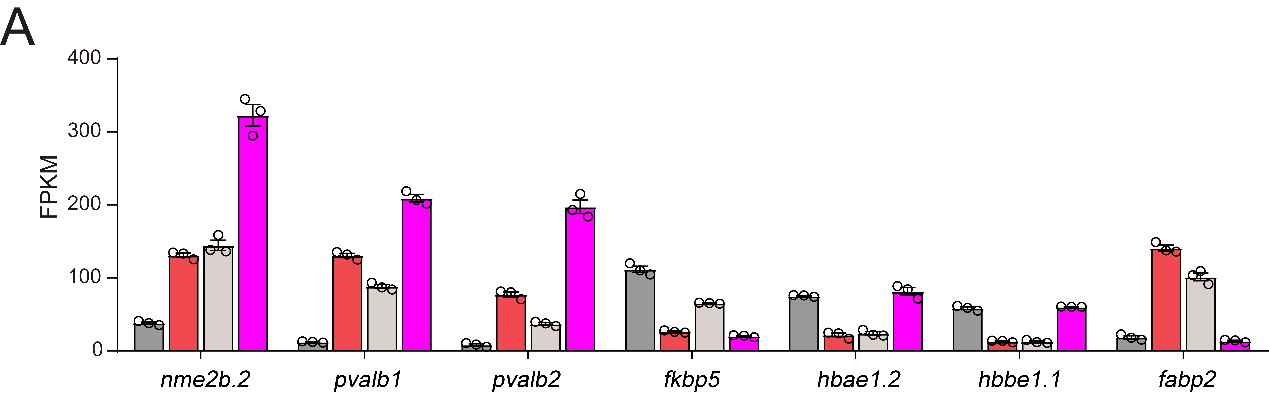


Figure S9


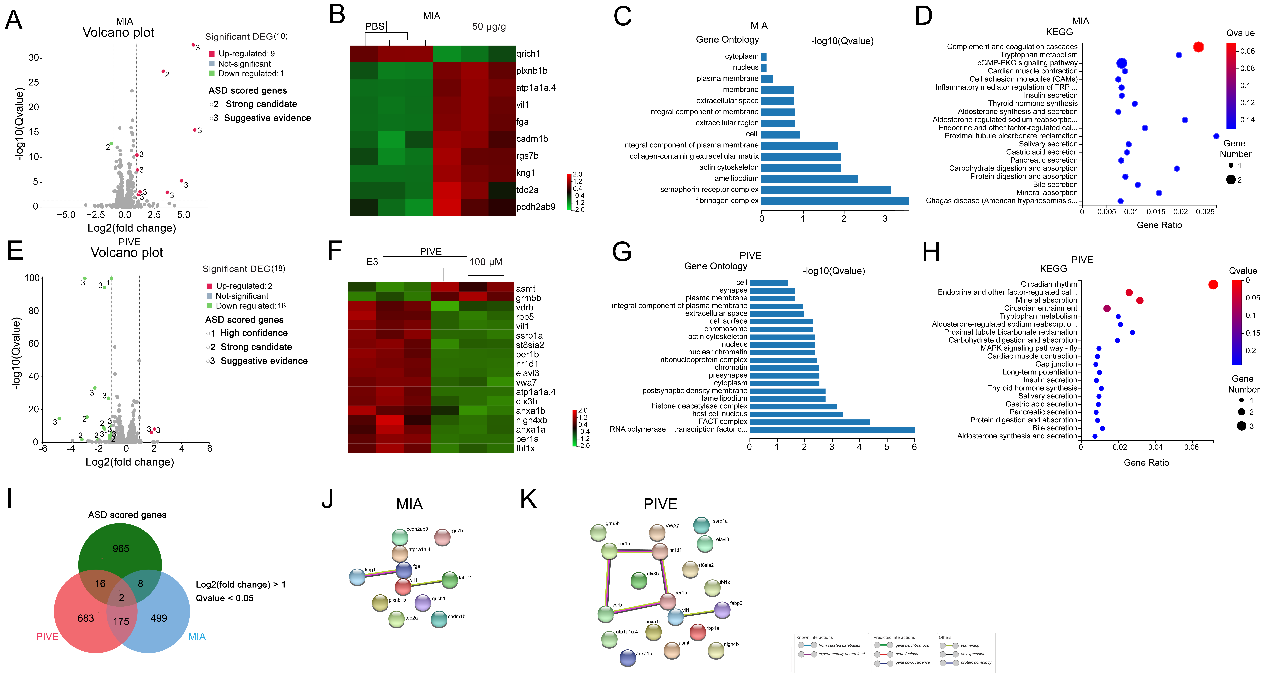


Figure S10


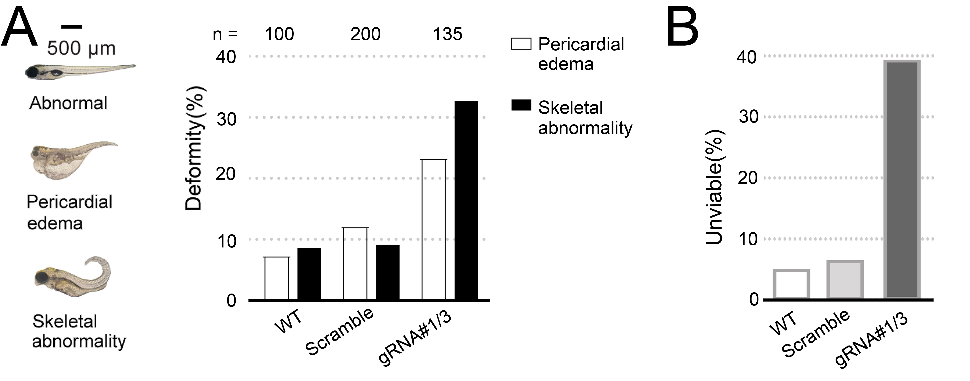


Figure S11


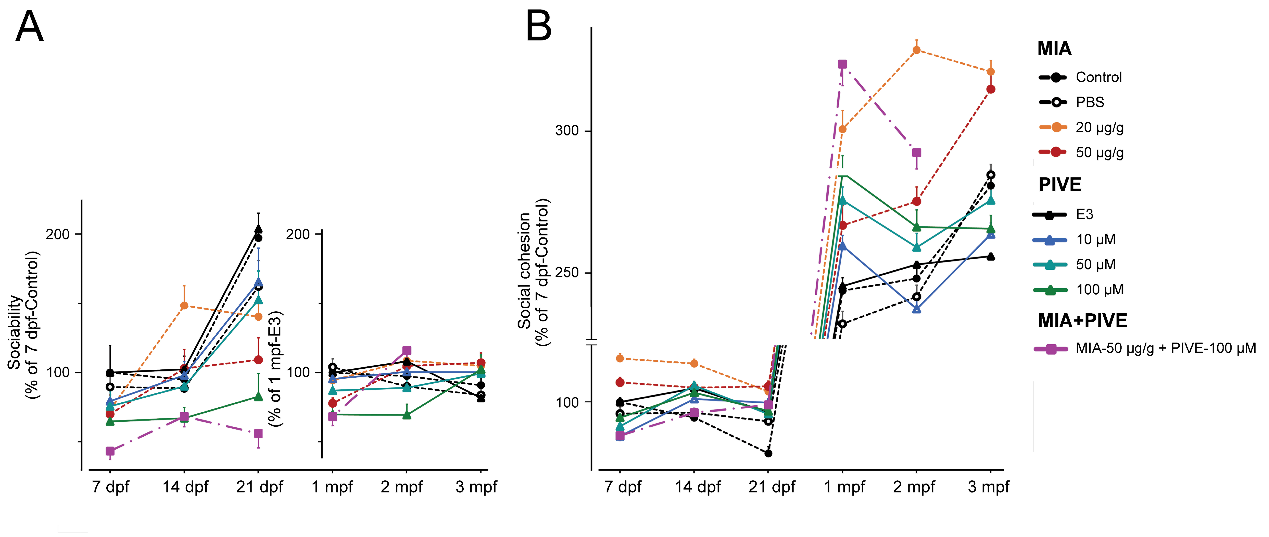


Figure S12


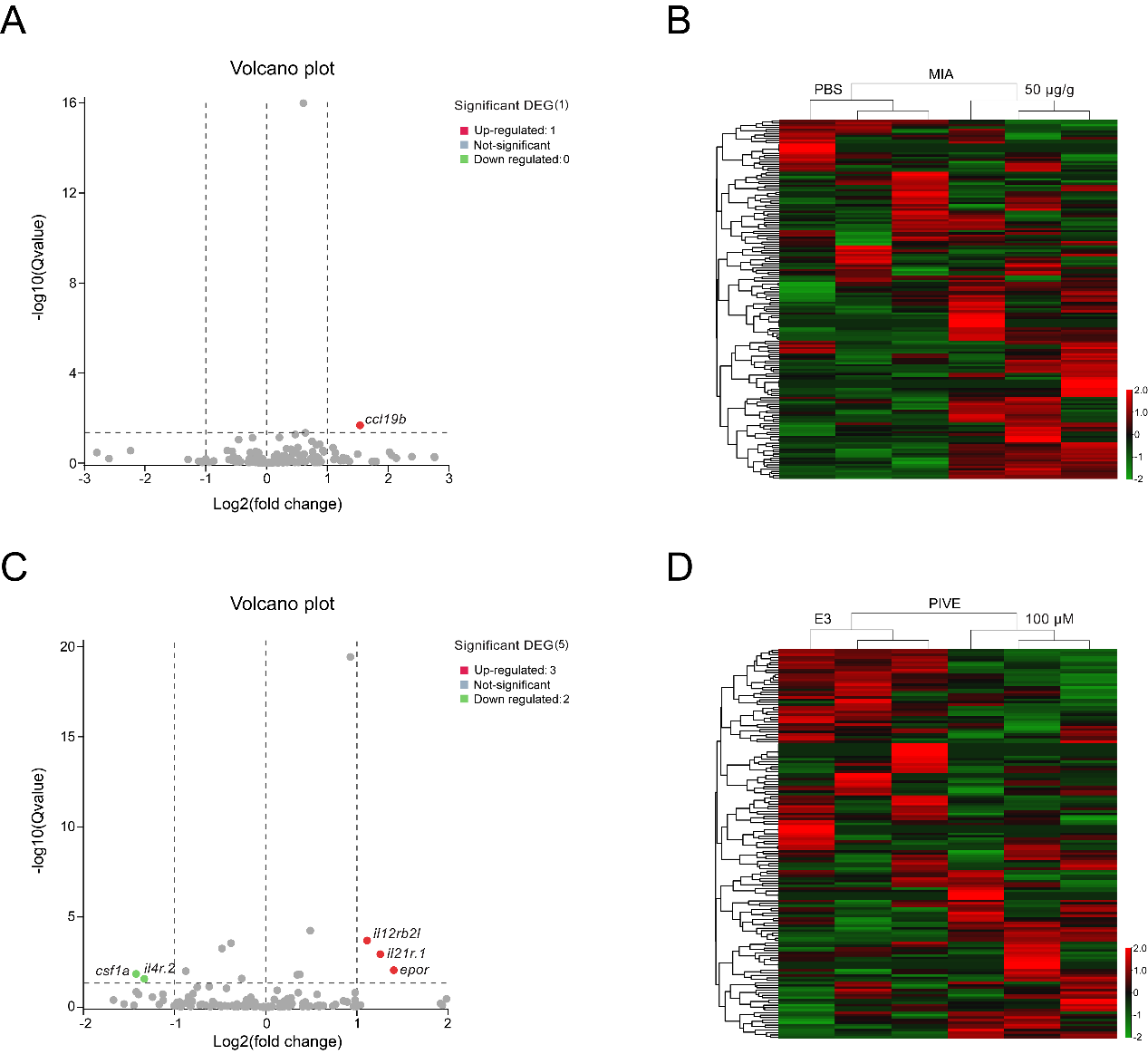

Supplement: Supplementary file 2 [file Data_Sheet_2.DOCX]
