# Supplementary material for: Poly(I:C)-exposed zebrafish shows autism-like behaviors which are ameliorated by fabp2 gene knockout
Source: Front Mol Neurosci. 2023 Jan 5;15:1068019. doi: 10.3389/fnmol.2022.1068019 (PMC9849760; doi:10.3389/fnmol.2022.1068019)
Supplement: Supplementary file 5 [file Data_Sheet_5.DOCX]

**Additional file 11: File S4.** Basic data (*n*, M ± SEM or Media ± Percentile) of each group for Figure 1-7.

- **Figure 1**
- **Fig. 1Ba**

| **MIA** | | | | |
| --- | --- | --- | --- | --- |
|  | **Age** | **Tissue** | ***n*** | **M ± SEM** |
| PBS | 1dpf | Liver | 4 | 1.00±0.28 |
|  | 1dpf | Brain | 5 | 1.00±0.16 |
|  | 1dpf | Spleen | 3 | 1.00±0.14 |
|  | 1dpf | Intestinal tissue | 5 | 1.00±0.16 |
| 50μg/g | 1dpf | Liver | 5 | 0.77±0.17 |
|  | 1dpf | Brain | 5 | 0.98±0.18 |
|  | 1dpf | Spleen | 3 | 2.55±0.56 |
|  | 1dpf | Intestinal tissue | 4 | 0.13±0.04 |

**Step 1. Normal distribution analysis**

**Normality**

| **Shapiro-Wilk test** | | | | |
| --- | --- | --- | --- | --- |
| **Fig** | **W** | **df** | **P** | **Sig.** |
| 1-Ba | 0.8662 | 34 | 0.0007 | ** |

****nonnormal distribution**

**Step 2. compare within PBS**

Kruskal-Wallis test

| **df** | **H** | **P** | **Sig.** |
| --- | --- | --- | --- |
| 3 | 0.2824 | 0.9696 | ns |

**Step 3. compare within MIA-50μg/g**

Kruskal-Wallis test

| **df** | **H** | **P** | **Sig.** |
| --- | --- | --- | --- |
| 3 | 11.5500 | 0.0010 | * |

Bonferroni's multiple comparisons test

| **Treatment** | **Comparison** | **Z** | **P** | **Sig.** |
| --- | --- | --- | --- | --- |
| 50μg/g | Brain vs. Liver | 0.31 | >0.9999 | ns |
| 50μg/g | Spleen vs. Liver | 1.95 | 0.3054 | ns |
| 50μg/g | Intestinal tissue vs. Liver | 1.71 | 0.5212 | ns |
| 50μg/g | Spleen vs. Brain | 1.68 | 0.5563 | ns |
| 50μg/g | Intestinal tissue vs. Brain | 2.01 | 0.2682 | ns |
| 50μg/g | Intestinal tissue vs. Spleen | 3.37 | 0.0045 | * |

**Step 4. MIA-50μg/g vs. PBS**

Multiple Mann-Whitney tests with Bonferroni's correction

| **Tissue** | **Comparison** | **U** | **P** | **Sig.** |
| --- | --- | --- | --- | --- |
| Liver | 50μg/g vs. PBS | 5.00 | >0.9999 | ns |
| Brain | 50μg/g vs. PBS | 12.00 | >0.9999 | ns |
| Spleen | 50μg/g vs. PBS | 0.00 | 0.4000 | ns |
| Intestinal tissue | 50μg/g vs. PBS | 0.00 | 0.0635 | ns |

- **Fig.1Bb**

| **MIA** | | | | **PIVE-1** | | | | **PIVE-2** | | | |
| --- | --- | --- | --- | --- | --- | --- | --- | --- | --- | --- | --- |
|  | **Age** | **N** | **M±SEM** |  | **Age** | **N** | **M±SEM** |  | **Age** | ***n*** | **M±SEM** |
| PBS | 1dpf | 4 | 1.00±0.38 | E3 | 1dpf | 4 | 1.00±0.07 | E3 | 1dpf | 6 | 1.00±0.05 |
| 50μg/g | 1dpf | 6 | 2.24±0.36 | 100M | 1dpf | 4 | 1.07±0.14 | 100M | 1dpf | 7 | 2.28±0.22 |

**Step 1. Normal distribution analysis**

**Normality**

| **Shapiro-Wilk test** | | | | |
| --- | --- | --- | --- | --- |
| **Fig** | **W** | **df** | **P** | **Sig.** |
| 1-Bb | 0.9244 | 34 | 0.0309 | * |

***nonnormal distribution**

**Step 2. compare within MIA、PIVE-1、PIVE-2**

Mann-Whitney tests

| **Group** | **Comparison** | **U** | **P** | **Sig.** |
| --- | --- | --- | --- | --- |
| MIA | 50μg/g vs. PBS | 3.00 | 0.0667 | ns |
| PIVE-1 | 100μM vs. E3 | 6.00 | 0.6857 | ns |
| PIVE-2 | 100μM vs. E3 | 0.00 | 0.0012 | * |

**Step 3. 50 μg/g vs. 100 μM (PIVE-1 & PIVE-2)**

Kruskal-Wallis test

| **df** | **H** | **P** | **Sig.** |
| --- | --- | --- | --- |
| 2 | 7.4370 | 0.0159 | * |

Bonferroni's multiple comparisons test

| **Comparison** | **Z** | **P** | **Sig.** |
| --- | --- | --- | --- |
| 100μM (PIVE-1) vs. 50μg/g | 2.30 | 0.0640 | ns |
| 100μM (PIVE-)2 vs. 50μg/g | 0.23 | >0.9999 | ns |
| 100μM (PIVE-2)) vs. 100μM (PIVE-1 | 2.57 | 0.0303 | * |

- **Fig.1Ca**

| **MIA** | | | | |
| --- | --- | --- | --- | --- |
|  | **Age** | **Tissue** | ***n*** | **M ± SEM** |
| PBS | 1dpf | Liver | 3 | 1.00±0.12 |
|  | 1dpf | Brain | 4 | 1.00±0.17 |
|  | 1dpf | Spleen | 4 | 1.00±0.11 |
|  | 1dpf | Intestinal tissue | 5 | 1.00±0.13 |
| 50μg/g | 1dpf | Liver | 4 | 0.40±0.09 |
|  | 1dpf | Brain | 5 | 0.58±0.10 |
|  | 1dpf | Spleen | 3 | 0.80±0.24 |
|  | 1dpf | Intestinal tissue | 4 | 0.13±0.05 |

**Step 1. Normal distribution analysis**

**Normality**

| **Shapiro-Wilk test** | | | | |
| --- | --- | --- | --- | --- |
| **Fig** | **W** | **df** | **P** | **Sig.** |
| 1-Ca | 0.9730 | 32 | 0.5860 | ns |

**ns: normal distribution**

**Step 2. compare within PBS and MIA 50 μg/g**

**Two-way ANOVA**

| **Variation** | **df** | **F** | **P** | **Sig.** |
| --- | --- | --- | --- | --- |
| Interaction | 3 | 2.399 | 0.0928 | ns |
| MIA | 1 | 32.070 | <0.0001 | ** |
| Tissue | 3 | 2.399 | 0.0928 | ns |

Šídák's multiple comparisons test

| **Tissue** | **Comparison** | **df** | **t** | **P** | **Sig.** |
| --- | --- | --- | --- | --- | --- |
| Liver | 50 μg/g vs. PBS | 24 | 3.057 | 0.0215 | $ |
| Brain | 50 μg/g vs. PBS | 24 | 2.462 | 0.0828 | ns |
| Spleen | 50 μg/g vs. PBS | 24 | 1.013 | 0.7875 | ns |
| Intestinal tissue | 50 μg/g vs. PBS | 24 | 5.039 | 0.0002 | $$ |

Šídák's multiple comparisons test

| **Treatment** | **Comparison** | **df** | **t** | **P** | **Sig.** |
| --- | --- | --- | --- | --- | --- |
| PBS | Brain vs. Liver | 24 | 0.000 | >0.9999 | ns |
|  | Spleen vs. Liver | 24 | 0.000 | >0.9999 | ns |
|  | Intestinal tissue vs. Liver | 24 | 0.000 | >0.9999 | ns |
|  | Spleen vs. Brain | 24 | 0.000 | >0.9999 | ns |
|  | Intestinal tissue vs. Brain | 24 | 0.000 | >0.9999 | ns |
|  | Intestinal tissue vs. Spleen | 24 | 0.000 | >0.9999 | ns |
| 50 μg/g | Brain vs. Liver | 24 | 1.018 | 0.8999 | ns |
|  | Spleen vs. Liver | 24 | 2.044 | 0.2746 | ns |
|  | Intestinal tissue vs. Liver | 24 | 1.478 | 0.6289 | ns |
|  | Spleen vs. Brain | 24 | 1.202 | 0.8089 | ns |
|  | Intestinal tissue vs. Brain | 24 | 2.577 | 0.0953 | ns |
|  | Intestinal tissue vs. Spleen | 24 | 3.413 | 0.0136 | * |

- **Fig.1Cb**

| **MIA** | | | **PIVE-1** | | | | | **PIVE-2** | | | | |
| --- | --- | --- | --- | --- | --- | --- | --- | --- | --- | --- | --- | --- |
|  | **Age** | **N** | **M ± SEM** |  | **Age** | **N** | **M ± SEM** |  | **Age** | ***n*** | **M ± SEM** | |
| PBS | 1dpf | 5 | 1.00±0.35 | E3 | 1dpf | 4 | 1.00 ±0.07 | E3 | 1dpf | 7 | 1.00±0.11 | |
| 50μg/g | 1dpf | 7 | 1.10±0.22 | 100μM | 1dpf | 4 | 0.81 ±0.19 | 100M | 1dpf | 5 | 2.26±0.20 |  |

**Step 1. Normal distribution analysis**

**Normality**

| **Shapiro-Wilk test** | | | | |
| --- | --- | --- | --- | --- |
| **Fig** | **W** | **df** | **P** | **Sig.** |
| 1-Cb | 0.8864 | 32 | 0.0028 | * |

***nonnormal distribution**

**Step 2. compare within MIA、PIVE-1、PIVE-2**

Mann-Whitney tests

| **Group** | **Comparison** | **U** | **P** | **Sig.** |
| --- | --- | --- | --- | --- |
| MIA | 50 μg/g vs. PBS | 14.00 | 0.6389 | ns |
| PIVE-1 | 100μM vs. E3 | 5.00 | 0.4857 | ns |
| PIVE-2 | 100μM vs. E3 | 0.00 | 0.0025 | * |

**Step 3. MIA50 μg/g vs. 100 μM (PIVE-1 & PIVE-2)**

Kruskal-Wallis test

| **df** | **H** | **P** | **Sig.** |
| --- | --- | --- | --- |
| 2 | 8.3470 | 0.0074 | * |

Bonferroni's multiple comparisons test

| **Comparison** | **Z** | **P** | **Sig.** |
| --- | --- | --- | --- |
| 100 μM (PIVE-1) vs. 50 μg/g | 0.8020 | >0.9999 | ns |
| 100 μM (PIVE-2) vs. 50 μg/g | 2.2450 | 0.0744 | ns |
| 100 μM (PIVE-2) vs. 100 μM (PIVE-1) | 2.7080 | 0.0203 | * |

- **Fig.1Da**

| **MIA** | | | | |
| --- | --- | --- | --- | --- |
|  | **Age** | **Tissue** | ***n*** | **M ± SEM** |
| PBS | 1dpf | Liver | 3 | 1.00±0.29 |
|  | 1dpf | Brain | 3 | 1.00±0.31 |
|  | 1dpf | Spleen | 4 | 1.00±0.18 |
|  | 1dpf | Intestinal tissue | 4 | 1.00±0.11 |
| 50μg/g | 1dpf | Liver | 4 | 0.25±0.05 |
|  | 1dpf | Brain | 5 | 0.24±0.03 |
|  | 1dpf | Spleen | 3 | 2.21±0.81 |
|  | 1dpf | Intestinal tissue | 4 | 0.15±0.05 |

**Step 1. Normal distribution analysis**

**Normality**

| **Shapiro-Wilk test** | | | | |
| --- | --- | --- | --- | --- |
| **Fig** | **W** | **df** | **P** | **Sig.** |
| 1-Da | 0.7654 | 30 | <0.0001 | ** |

****nonnormal distribution**

**Step 2. compare within PBS**

Kruskal-Wallis test

| **df** | **H** | **P** | **Sig.** |
| --- | --- | --- | --- |
| 3 | 0.0762 | 0.9963 | ns |

**Step 3. compare within MIA50 μg/g**

Kruskal-Wallis test

| **df** | **H** | **P** | **Sig.** |
| --- | --- | --- | --- |
| 3 | 8.5240 | 0.0186 | * |

Bonferroni's multiple comparisons test

| **Treatment** | **Comparison** | **Z** | **P** | **Sig.** |
| --- | --- | --- | --- | --- |
| 50μg/g | Brain vs. Liver | 0.22 | >0.9999 | ns |
| 50μg/g | Spleen vs. Liver | 1.79 | 0.4431 | ns |
| 50μg/g | Intestinal tissue vs. Liver | 1.19 | >0.9999 | ns |
| 50μg/g | Spleen vs. Brain | 2.07 | 0.2303 | ns |
| 50μg/g | Intestinal tissue vs. Brain | 1.03 | >0.9999 | ns |
| 50μg/g | Intestinal tissue vs. Spleen | 2.89 | 0.0233 | * |

**Step 4. MIA50 μg/g vs. PBS**

Multiple Mann-Whitney tests with Bonferroni's correction

| **Tissue** | **Comparison** | **U** | **P** | **Sig.** |
| --- | --- | --- | --- | --- |
| Liver | 50 μg/g vs. PBS | 0.00 | 0.2286 | ns |
| Brain | 50 μg/g vs. PBS | 0.00 | 0.1429 | ns |
| Spleen | 50 μg/g vs. PBS | 1.00 | 0.4571 | ns |
| Intestinal tissue | 50 μg/g vs. PBS | 0.00 | 0.1143 | ns |

- **Fig.1Db**

| **MIA** | | | | **PIVE-1** | | | | **PIVE-2** | | | |
| --- | --- | --- | --- | --- | --- | --- | --- | --- | --- | --- | --- |
|  | **Age** | ***n*** | **M ± SEM** |  | **Age** | ***n*** | **M ± SEM** |  | **Age** | ***n*** | **M ± SEM** |
| PBS | 1dpf | 3 | 1.00±0.37 | E3 | 1dpf | 4 | 1.00±0.11 | E3 | 1dpf | 7 | 1.00±0.08 |
| 50μg/g | 1dpf | 6 | 2.32±0.24 | 100M | 1dpf | 4 | 0.80±0.11 | 100M | 1dpf | 5 | 2.22±0.48 |

**Step 1. Normal distribution analysis**

**Normality**

| **Shapiro-Wilk test** | | | | |
| --- | --- | --- | --- | --- |
| **Fig** | **W** | **df** | **P** | **Sig.** |
| 1-Db | 0.8642 | 29 | 0.0015 | * |

***nonnormal distribution**

**Step 2. compare within MIA、PIVE-1、PIVE-2**

Mann-Whitney tests

| **Group** | **Comparison** | **U** | **P** | **Sig.** |
| --- | --- | --- | --- | --- |
| MIA | 50μg/g vs. PBS | 1.00 | 0.0476 | * |
| PIVE-1 | 100μM vs. E3 | 5.00 | 0.4857 | ns |
| PIVE-2 | 100μM vs. E3 | 1.00 | 0.0051 | * |

**Step 3. 50 μg/g vs. 100 μM (PIVE-1 & PIVE-2)**

Kruskal-Wallis test

| **df** | **H** | **P** | **Sig.** |
| --- | --- | --- | --- |
| 2 | 8.323 | 0.0066 | * |

Bonferroni's multiple comparisons test

| **Comparison** | **Z** | **P** | **Sig.** |
| --- | --- | --- | --- |
| 100 μM (PIVE-1) vs. 50 μg/g | 2.71 | 0.0200 | * |
| 100 μM (PIVE-2) vs. 50 μg/g | 0.27 | >0.9999 | ns |
| 100 μM (PIVE-2) vs. 100 μM (PIVE-1) | 2.37 | 0.0538 | ns |

- **Fig.1E**

| **MIA** | | | | **PIVE** | | | |
| --- | --- | --- | --- | --- | --- | --- | --- |
|  | **Age** | ***n*** | **M ± SEM** |  | **Age** | ***n*** | **M ± SEM** |
| Control | 7dpf | 6 | 41.67±4.01 | E3 | 7dpf | 6 | 45.00±4.28 |
|  | 14dpf | 6 | 55.00±5.00 |  | 14dpf | 6 | 43.33±4.22 |
|  | 21dpf | 6 | 58.33±7.03 |  | 21dpf | 6 | 46.67±4.22 |
| PBS | 7dpf | 6 | 41.67±5.43 | 10μM | 7dpf | 6 | 43.33±3.33 |
|  | 14dpf | 6 | 50.00±5.16 |  | 14dpf | 6 | 43.33±4.94 |
|  | 21dpf | 6 | 51.67±3.07 |  | 21dpf | 6 | 46.67±4.22 |
| 20μg/g | 7dpf | 6 | 43.33±3.33 | 50μM | 7dpf | 6 | 43.33±3.33 |
|  | 14dpf | 6 | 48.33±4.77 |  | 14dpf | 6 | 46.67±2.11 |
|  | 21dpf | 6 | 48.33±3.07 |  | 21dpf | 6 | 50.00±3.65 |
| 50μg/g | 7dpf | 6 | 55.00±6.19 | 100μM | 7dpf | 6 | 43.33±4.22 |
|  | 14dpf | 6 | 76.67±7.15 |  | 14dpf | 6 | 43.33±3.33 |
|  | 21dpf | 6 | 78.33±7.49 |  | 21dpf | 6 | 313.33±31.59 |

**Step 1. Normal distribution analysis**

**Normality**

| **Shapiro-Wilk test** | | | | |
| --- | --- | --- | --- | --- |
| **Fig** | **W** | **df** | **P** | **Sig.** |
| 1-E | 0.4033 | 144 | <0.0001 | ** |

****nonnormal distribution**

**Step 2. compare different ages within the same treatment**

**MIA-Control**

Kruskal-Wallis test

| **df** | **H** | **P** | **Sig.** |
| --- | --- | --- | --- |
| 2 | 4.5400 | 0.1041 | ns |

**MIA-PBS**

Kruskal-Wallis test

| **df** | **H** | **P** | **Sig.** |
| --- | --- | --- | --- |
| 2 | 2.3140 | 0.3178 | ns |

**MIA-20μg/g**

Kruskal-Wallis test

| **df** | **H** | **P** | **Sig.** |
| --- | --- | --- | --- |
| 2 | 1.2090 | 0.5900 | ns |

**MIA-50μg/g**

Kruskal-Wallis test

| **df** | **H** | **P** | **Sig.** |
| --- | --- | --- | --- |
| 2 | 5.0580 | 0.0773 | ns |

**PIVE-E3**

Kruskal-Wallis test

| **df** | **H** | **P** | **Sig.** |
| --- | --- | --- | --- |
| 2 | 0.4580 | 0.8075 | ns |

**PIVE-10μM**

Kruskal-Wallis test

| **df** | **H** | **P** | **Sig.** |
| --- | --- | --- | --- |
| 2 | 0.4850 | 0.8488 | ns |

**PIVE-50μM**

Kruskal-Wallis test

| **df** | **H** | **P** | **Sig.** |
| --- | --- | --- | --- |
| 2 | 1.6870 | 0.5090 | ns |

**PIVE-100μM**

Kruskal-Wallis test

| **df** | **H** | **P** | **Sig.** |
| --- | --- | --- | --- |
| 2 | 11.7700 | 0.0003 | ** |

Bonferroni's multiple comparisons test

| **Treatment** | **Comparison** | **Z** | **P** | **Sig.** |
| --- | --- | --- | --- | --- |
| 100 μM | 14dpf vs. 7dpf | 0.11 | >0.9999 | ns |
| 100 μM | 21dpf vs. 7dpf | 3.02 | 0.0075 | * |
| 100 μM | 21dpf vs. 14dpf | 2.91 | 0.0107 | * |

**Step 3. compare different treatments within the same age**

**MIA-7dpf**

Kruskal-Wallis test

| **df** | **H** | **P** | **Sig.** |
| --- | --- | --- | --- |
| 3 | 4.0850 | 0.2524 | ns |

**MIA-14dpf**

Kruskal-Wallis test

| **df** | **H** | **P** | **Sig.** |
| --- | --- | --- | --- |
| 3 | 8.1760 | 0.0425 | * |

Bonferroni's multiple comparisons test

| **Age** | **Comparison** | **Z** | **P** | **Sig.** |
| --- | --- | --- | --- | --- |
| 14dpf | PBS vs. Control | 0.65 | >0.9999 | ns |
| 14dpf | 20 μg/g vs. Control | 0.67 | >0.9999 | ns |
| 14dpf | 50 μg/g vs. Control | 1.81 | 0.4187 | ns |
| 14dpf | 20 μg/g vs. PBS | 0.02 | >0.9999 | ns |
| 14dpf | 50 μg/g vs. PBS | 2.46 | 0.0835 | ns |
| 14dpf | 50 μg/g vs. 20 μg/g | 2.48 | 0.0788 | ns |

**MIA-21dpf**

Kruskal-Wallis test

| **df** | **H** | **P** | **Sig.** |
| --- | --- | --- | --- |
| 3 | 8.4860 | 0.0370 | * |

Bonferroni's multiple comparisons test

| **Age** | **Comparison** | **Z** | **P** | **Sig.** |
| --- | --- | --- | --- | --- |
| 21dpf | PBS vs. Control | 0.55 | >0.9999 | ns |
| 21dpf | 20 μg/g vs. Control | 1.15 | >0.9999 | ns |
| 21dpf | 50 μg/g vs. Control | 1.62 | 0.6343 | ns |
| 21dpf | 20 μg/g vs. PBS | 0.60 | >0.9999 | ns |
| 21dpf | 50 μg/g vs. PBS | 2.17 | 0.1795 | ns |
| 21dpf | 50 μg/g vs. 20 μg/g | 2.77 | 0.0339 | * |

**PIVE-7dpf**

Kruskal-Wallis test

| **df** | **H** | **P** | **Sig.** |
| --- | --- | --- | --- |
| 3 | 0.1553 | 0.9845 | ns |

**PIVE-14dpf**

Kruskal-Wallis test

| **df** | **H** | **P** | **Sig.** |
| --- | --- | --- | --- |
| 3 | 0.7441 | 0.8628 | ns |

**PIVE-21dpf**

Kruskal-Wallis test

| **df** | **H** | **P** | **Sig.** |
| --- | --- | --- | --- |
| 3 | 13.7700 | 0.0032 | * |

Bonferroni's multiple comparisons test

| **Age** | **Comparison** | **Z** | **P** | **Sig.** |
| --- | --- | --- | --- | --- |
| 21dpf | 10μM vs. E3 | 0.000 | >0.9999 | ns |
| 21dpf | 50μM vs. E3 | 0.376 | >0.9999 | ns |
| 21dpf | 100μM vs. E3 | 3.135 | 0.0103 | * |
| 21dpf | 50μM vs. 10μM | 0.376 | >0.9999 | ns |
| 21dpf | 100μM vs. 10μM | 3.135 | 0.0103 | * |
| 21dpf | 100μM vs. 50μM | 2.759 | 0.0348 | * |

**Step 4. MIA vs. PIVE in same age**

**MIA-20 μg/g vs. PIVE-10/ 50/100 μM**

Multiple Mann-Whitney tests with Bonferroni's correction

| **Age** | **Comparison** | **U** | **P** | **Sig.** |
| --- | --- | --- | --- | --- |
| 7dpf | 10 μM vs. 20 μg/g | 18.00 | >0.9999 | ns |
| 14dpf | 10 μM vs. 20 μg/g | 13.50 | >0.9999 | ns |
| 21dpf | 10 μM vs. 20 μg/g | 17.00 | >0.9999 | ns |
| 7dpf | 50 μM vs. 20 μg/g | 18.00 | >0.9999 | ns |
| 14dpf | 50 μM vs. 20 μg/g | 18.00 | >0.9999 | ns |
| 21dpf | 50 μM vs. 20 μg/g | 18.00 | >0.9999 | ns |
| 7dpf | 100 μM vs. 20 μg/g | 17.00 | >0.9999 | ns |
| 14dpf | 100 μM vs. 20 μg/g | 12.50 | >0.9999 | ns |
| 21dpf | 100 μM vs. 20 μg/g | 0.00 | 0.0064 | % |

**MIA-50μg/g vs. PIVE-50 μM/100 μM**

Multiple Mann-Whitney tests with Bonferroni's correction

| **Age** | **Comparison** | **U** | **P** | **Sig.** |
| --- | --- | --- | --- | --- |
| 7dpf | 10 μM vs. 50 μg/g | 8.50 | 0.4221 | ns |
| 14dpf | 10 μM vs. 50 μg/g | 2.50 | 0.0455 | @ |
| 21dpf | 10 μM vs. 50 μg/g | 3.00 | 0.0584 | ns |
| 7dpf | 50μM vs. 50μg/g | 8.50 | 0.4221 | ns |
| 14dpf | 50μM vs. 50μg/g | 2.00 | 0.0325 | @ |
| 21dpf | 50μM vs. 50μg/g | 4.00 | 0.0779 | ns |
| 7dpf | 100μM vs. 50μg/g | 9.00 | 0.4935 | ns |
| 14dpf | 100μM vs. 50μg/g | 1.50 | 0.0260 | @ |
| 21dpf | 100μM vs. 50μg/g | 0.00 | 0.0065 | @ |

- **Fig.1F**

| **MIA** | | | | **PIVE** | | | |
| --- | --- | --- | --- | --- | --- | --- | --- |
|  | **Age** | ***n*** | **M ± SEM** |  | **Age** | ***n*** | **M ± SEM** |
| Control | 7dpf | 6 | 0.00±0.00 | E3 | 7dpf | 6 | 0.17±0.17 |
|  | 14dpf | 6 | 0.67±0.21 |  | 14dpf | 6 | 0.17±0.17 |
|  | 21dpf | 6 | 0.50±0.34 |  | 21dpf | 6 | 0.17±0.17 |
| PBS | 7dpf | 6 | 0.33±0.33 | 10μM | 7dpf | 6 | 0.17±0.17 |
|  | 14dpf | 6 | 0.17±0.17 |  | 14dpf | 6 | 0.67±0.33 |
|  | 21dpf | 6 | 0.33±0.33 |  | 21dpf | 6 | 0.33±0.21 |
| 20μg/g | 7dpf | 6 | 0.00±0.00 | 50μM | 7dpf | 6 | 0.33±0.21 |
|  | 14dpf | 6 | 0.17±0.17 |  | 14dpf | 6 | 0.17±0.17 |
|  | 21dpf | 6 | 1.17±0.17 |  | 21dpf | 6 | 0.17±0.17 |
| 50μg/g | 7dpf | 6 | 0.67±0.33 | 100μM | 7dpf | 6 | 0.00±0.00 |
|  | 14dpf | 6 | 0.50±0.22 |  | 14dpf | 6 | 0.33±0.21 |
|  | 21dpf | 6 | 2.50±1.12 |  | 21dpf | 6 | 35.83±4.35 |

**Step 1. Normal distribution analysis**

**Normality**

| **Shapiro-Wilk test** | | | | |
| --- | --- | --- | --- | --- |
| **Fig** | **W** | **df** | **P** | **Sig.** |
| 1-F | 0.2632 | 144 | <0.0001 | ** |

****nonnormal distribution**

**Step 2. compare different ages within the same treatment**

**MIA-Control**

Kruskal-Wallis test

| **df** | **H** | **P** | **Sig.** |
| --- | --- | --- | --- |
| 2 | 4.991 | 0.0845 | ns |

**MIA-PBS**

Kruskal-Wallis test

| **df** | **H** | **P** | **Sig.** |
| --- | --- | --- | --- |
| 2 | 0.0208 | >0.9999 | ns |

**MIA-20 μg/g**

Kruskal-Wallis test

| **df** | **H** | **P** | **Sig.** |
| --- | --- | --- | --- |
| 2 | 13.65 | 0.0010 | * |

Bonferroni's multiple comparisons test

| **Treatment** | **Comparison** | **Z** | **P** | **Sig.** |
| --- | --- | --- | --- | --- |
| 20 μg/g | 14dpf vs. 7dpf | 0.54 | >0.9999 | ns |
| 20 μg/g | 21dpf vs. 7dpf | 3.43 | 0.0018 | * |
| 20 μg/g | 21dpf vs. 14dpf | 2.90 | 0.0113 | * |

**MIA-50μg/g**

Kruskal-Wallis test

| **df** | **H** | **P** | **Sig.** |
| --- | --- | --- | --- |
| 2 | 0.8655 | 0.6886 | ns |

**PIVE-E3**

Kruskal-Wallis test

| **df** | **H** | **P** | **Sig.** |
| --- | --- | --- | --- |
| 2 | 0 | >0.9999 | ns |

**PIVE-10μM**

Kruskal-Wallis test

| **df** | **H** | **P** | **Sig.** |
| --- | --- | --- | --- |
| 2 | 1.722 | 0.5273 | ns |

**PIVE-50μM**

Kruskal-Wallis test

| **df** | **H** | **P** | **Sig.** |
| --- | --- | --- | --- |
| 2 | 0.6071 | >0.9999 | ns |

**PIVE-100μM**

Kruskal-Wallis test

| **df** | **H** | **P** | **Sig.** |
| --- | --- | --- | --- |
| 2 | 14.23 | <0.0001 | ** |

Bonferroni's multiple comparisons test

| **Treatment** | **Comparison** | **Z** | **P** | **Sig.** |
| --- | --- | --- | --- | --- |
| 100 μM | 14dpf vs. 7dpf | 0.71 | >0.9999 | ns |
| 100 μM | 21dpf vs. 7dpf | 3.56 | 0.0011 | * |
| 100 μM | 21dpf vs. 14dpf | 2.85 | 0.0131 | * |

**Step 3. compare different treatments within the same age**

**MIA-7dpf**

Kruskal-Wallis test

| **df** | **H** | **P** | **Sig.** |
| --- | --- | --- | --- |
| 3 | 6.479 | 0.0905 | ns |

**MIA-14dpf**

Kruskal-Wallis test

| **df** | **H** | **P** | **Sig.** |
| --- | --- | --- | --- |
| 3 | 4.6 | 0.2035 | ns |

**MIA-21dpf**

Kruskal-Wallis test

| **df** | **H** | **P** | **Sig.** |
| --- | --- | --- | --- |
| 3 | 5.356 | 0.1475 | ns |

**PIVE-7dpf**

Kruskal-Wallis test

| **df** | **H** | **P** | **Sig.** |
| --- | --- | --- | --- |
| 3 | 2.3 | 0.5125 | ns |

**PIVE-14dpf**

Kruskal-Wallis test

| **df** | **H** | **P** | **Sig.** |
| --- | --- | --- | --- |
| 3 | 2.505 | 0.4743 | ns |

**PIVE-21dpf**

Kruskal-Wallis test

| **df** | **H** | **P** | **Sig.** |
| --- | --- | --- | --- |
| 3 | 16.47 | 0.0009 | ** |

Bonferroni's multiple comparisons test

| **Age** | **Comparison** | **Z** | **P** | **Sig.** |
| --- | --- | --- | --- | --- |
| 21dpf | 10 μM vs. E3 | 0.41 | >0.9999 | ns |
| 21dpf | 50 μM vs. E3 | 0.00 | >0.9999 | ns |
| 21dpf | 100 μM vs. E3 | 3.43 | 0.0036 | * |
| 21dpf | 50 μM vs. 10 μM | 0.4114 | >0.9999 | ns |
| 21dpf | 100 μM vs. 10 μM | 3.017 | 0.0153 | * |
| 21dpf | 100 μM vs. 50 μM | 3.428 | 0.0036 | * |

**Step 4. MIA vs. PIVE in same age**

**MIA-20μg/g vs. PIVE-10/50/100μM**

Multiple Mann-Whitney tests with Bonferroni's correction

| **Age** | **Comparison** | **U** | **P** | **Sig.** |
| --- | --- | --- | --- | --- |
| 7dpf | 10μM vs. 20μg/g | 15.00 | >0.9999 | ns |
| 14dpf | 10μM vs. 20μg/g | 11.50 | >0.9999 | ns |
| 21dpf | 10μM vs. 20μg/g | 5.00 | 0.1364 | ns |
| 7dpf | 50μM vs. 20μg/g | 9.00 | 0.5455 | ns |
| 14dpf | 50μM vs. 20μg/g | 12.00 | >0.9999 | ns |
| 21dpf | 50μM vs. 20μg/g | 18.00 | >0.9999 | ns |
| 7dpf | 100μM vs. 20μg/g | 18.00 | >0.9999 | ns |
| 14dpf | 100μM vs. 20μg/g | 15.00 | >0.9999 | ns |
| 21dpf | 100μM vs. 20μg/g | 0.00 | 0.0065 | % |

**MIA-50μg/g vs. PIVE-10/50/100μM**

Multiple Mann-Whitney tests with Bonferroni's correction

| **Age** | **Comparison** | **U** | **P** | **Sig.** |
| --- | --- | --- | --- | --- |
| 7dpf | 10μM vs. 50μg/g | 11.50 | >0.9999 | ns |
| 14dpf | 10μM vs. 50μg/g | 16.50 | >0.9999 | ns |
| 21dpf | 10μM vs. 50μg/g | 12.00 | 0.9545 | ns |
| 7dpf | 50μM vs. 50μg/g | 18.00 | >0.9999 | ns |
| 14dpf | 50μM vs. 50μg/g | 18.00 | >0.9999 | ns |
| 21dpf | 50μM vs. 50μg/g | 18.00 | >0.9999 | ns |
| 7dpf | 100μM vs. 50μg/g | 9.00 | 0.5455 | ns |
| 14dpf | 100μM vs. 50μg/g | 15.00 | >0.9999 | ns |
| 21dpf | 100μM vs. 50μg/g | 0.00 | 0.0065 | @ |

- **Fig.1G**

| **MIA** | | | | **PIVE** | | | |
| --- | --- | --- | --- | --- | --- | --- | --- |
|  | **Age** | ***n*** | **M ± SEM** |  | **Age** | ***n*** | **M ± SEM** |
| Control | 7dpf | 6 | 23.33±2.11 | E3 | 7dpf | 6 | 28.33±3.07 |
|  | 14dpf | 6 | 25.00±3.42 |  | 14dpf | 6 | 18.33±1.67 |
|  | 21dpf | 6 | 28.33±1.67 |  | 21dpf | 6 | 21.67±1.67 |
| PBS | 7dpf | 6 | 23.33±2.11 | 10μM | 7dpf | 6 | 26.67±2.11 |
|  | 14dpf | 6 | 23.33±2.11 |  | 14dpf | 6 | 23.33±2.11 |
|  | 21dpf | 6 | 26.67±2.11 |  | 21dpf | 6 | 23.33±2.11 |
| 20μg/g | 7dpf | 6 | 20.00±2.58 | 50μM | 7dpf | 6 | 21.67±1.67 |
|  | 14dpf | 6 | 28.33±1.67 |  | 14dpf | 6 | 21.67±1.67 |
|  | 21dpf | 6 | 25.00±3.42 |  | 21dpf | 6 | 26.67±3.33 |
| 50μg/g | 7dpf | 6 | 30.00±2.58 | 100μM | 7dpf | 6 | 21.67±1.67 |
|  | 14dpf | 6 | 26.67±2.11 |  | 14dpf | 6 | 20.00±2.58 |
|  | 21dpf | 6 | 30.00±3.65 |  | 21dpf | 6 | 86.67±8.03 |

**Step 1. Normal distribution analysis**

**Normality**

| **Shapiro-Wilk test** | | | | |
| --- | --- | --- | --- | --- |
| **Fig** | **W** | **df** | **P** | **Sig.** |
| 1-G | 0.5414 | 144 | <0.0001 | ** |

****nonnormal distribution**

**Step 2. compare different ages within the same treatment**

**MIA-Control**

Kruskal-Wallis test

| **df** | **H** | **P** | **Sig.** |
| --- | --- | --- | --- |
| 2 | 2.744 | 0.2709 | ns |

**MIA-PBS**

Kruskal-Wallis test

| **df** | **H** | **P** | **Sig.** |
| --- | --- | --- | --- |
| 2 | 1.7 | 0.5886 | ns |

**MIA-20μg/g**

Kruskal-Wallis test

| **df** | **H** | **P** | **Sig.** |
| --- | --- | --- | --- |
| 2 | 4.958 | 0.0893 | ns |

**MIA-50μg/g**

Kruskal-Wallis test

| **df** | **H** | **P** | **Sig.** |
| --- | --- | --- | --- |
| 2 | 0.8863 | 0.7523 | ns |

**PIVE-E3**

Kruskal-Wallis test

| **df** | **H** | **P** | **Sig.** |
| --- | --- | --- | --- |
| 2 | 7.279 | 0.0252 | ns |

Bonferroni's multiple comparisons test

| **Treatment** | **Comparison** | **Z** | **P** | **Sig.** |
| --- | --- | --- | --- | --- |
| E3 | 14dpf vs. 7dpf | 2.66 | 0.0234 | * |
| E3 | 21dpf vs. 7dpf | 1.72 | 0.2566 | ns |
| E3 | 21dpf vs. 14dpf | 0.94 | >0.9999 | ns |

**PIVE-10μM**

Kruskal-Wallis test

| **df** | **H** | **P** | **Sig.** |
| --- | --- | --- | --- |
| 2 | 1.7 | 0.5886 | ns |

**PIVE-50μM**

Kruskal-Wallis test

| **df** | **H** | **P** | **Sig.** |
| --- | --- | --- | --- |
| 2 | 2.41 | 0.4265 | ns |

**PIVE-100μM**

Kruskal-Wallis test

| **df** | **H** | **P** | **Sig.** |
| --- | --- | --- | --- |
| 2 | 13.2 | 0.0002 | ** |

Bonferroni's multiple comparisons test

| **Treatment** | **Comparison** | **Z** | **P** | **Sig.** |
| --- | --- | --- | --- | --- |
| 100 μM | 14dpf vs. 7dpf | 0.29 | >0.9999 | ns |
| 100 μM | 21dpf vs. 7dpf | 2.99 | 0.0085 | * |
| 100 μM | 21dpf vs. 14dpf | 3.28 | 0.0032 | * |

**Step 3. compare different treatments within the same age**

**MIA-7dpf**

Kruskal-Wallis test

| **df** | **H** | **P** | **Sig.** |
| --- | --- | --- | --- |
| 3 | 7.042 | 0.0706 | ns |

**MIA-14dpf**

Kruskal-Wallis test

| **df** | **H** | **P** | **Sig.** |
| --- | --- | --- | --- |
| 3 | 3.152 | 0.3688 | ns |

**MIA-21dpf**

Kruskal-Wallis test

| **df** | **H** | **P** | **Sig.** |
| --- | --- | --- | --- |
| 3 | 1.204 | 0.7522 | ns |

**PIVE-7dpf**

Kruskal-Wallis test

| **df** | **H** | **P** | **Sig.** |
| --- | --- | --- | --- |
| 3 | 6.14 | 0.1050 | ns |

**PIVE-14dpf**

Kruskal-Wallis test

| **df** | **H** | **P** | **Sig.** |
| --- | --- | --- | --- |
| 3 | 3.264 | 0.3527 | ns |

**PIVE-21dpf**

Kruskal-Wallis test

| **df** | **H** | **P** | **Sig.** |
| --- | --- | --- | --- |
| 3 | 15.76 | 0.0013 | ** |

Bonferroni's multiple comparisons test

| **Age** | **Comparison** | **Z** | **P** | **Sig.** |
| --- | --- | --- | --- | --- |
| 21dpf | 10 μM vs. E3 | 0.37 | >0.9999 | ns |
| 21dpf | 50 μM vs. E3 | 0.88 | >0.9999 | ns |
| 21dpf | 100 μM vs. E3 | 3.58 | 0.0021 | * |
| 21dpf | 50 μM vs. 10 μM | 0.5049 | >0.9999 | ns |
| 21dpf | 100 μM vs. 10 μM | 3.205 | 0.0081 | ns |
| 21dpf | 100 μM vs. 50 μM | 2.7 | 0.0416 | ns |

**Step 4. MIA vs. PIVE in same age**

**MIA-20μg/g vs. PIVE-10/50/100μM**

Multiple Mann-Whitney tests with Bonferroni's correction

| **Age** | **Comparison** | **U** | **P** | **Sig.** |
| --- | --- | --- | --- | --- |
| 7dpf | 10μM vs. 20μg/g | 8.00 | 0.5325 | ns |
| 14dpf | 10μM vs. 20μg/g | 9.00 | 0.7273 | ns |
| 21dpf | 10μM vs. 20μg/g | 14.00 | >0.9999 | ns |
| 7dpf | 50μM vs. 20μg/g | 15.50 | >0.9999 | ns |
| 14dpf | 50μM vs. 20μg/g | 6.00 | 0.2403 | ns |
| 21dpf | 50μM vs. 20μg/g | 17.50 | >0.9999 | ns |
| 7dpf | 100μM vs. 20μg/g | 15.50 | >0.9999 | ns |
| 14dpf | 100μM vs. 20μg/g | 5.50 | 0.2013 | ns |
| 21dpf | 100μM vs. 20μg/g | 0.00 | 0.0065 | % |

**MIA-50μg/g vs. PIVE-10/50/100μM**

Multiple Mann-Whitney tests with Bonferroni's correction

| **Age** | **Comparison** | **U** | **P** | **Sig.** |
| --- | --- | --- | --- | --- |
| 7dpf | 10μM vs. 50μg/g | 13.00 | >0.9999 | ns |
| 14dpf | 10μM vs. 50μg/g | 12.00 | >0.9999 | ns |
| 21dpf | 10μM vs. 50μg/g | 10.00 | 0.8247 | ns |
| 7dpf | 50μM vs. 50μg/g | 5.50 | 0.2013 | ns |
| 14dpf | 50μM vs. 50μg/g | 9.00 | 0.7273 | ns |
| 21dpf | 50μM vs. 50μg/g | 14.00 | >0.9999 | ns |
| 7dpf | 100μM vs. 50μg/g | 5.50 | 0.2013 | ns |
| 14dpf | 100μM vs. 50μg/g | 8.00 | 0.5325 | ns |
| 21dpf | 100μM vs. 50μg/g | 0.00 | 0.0065 | @ |

- **Fig.1Ha**

| **MIA** | | | | |
| --- | --- | --- | --- | --- |
|  | **Age** | **Tissue** | ***n*** | **M ± SEM** |
| PBS | 1dpf | Liver | 6 | 303.33±12.56 |
|  | 1dpf | Brain | 6 | 123.33±6.67 |
|  | 1dpf | Spleen | 6 | 43.33±6.67 |
|  | 1dpf | Intestinal tissue | 6 | 53.33±3.33 |
| 50μg/g | 1dpf | Liver | 6 | 350.00±5.77 |
|  | 1dpf | Brain | 6 | 110.00±5.77 |
|  | 1dpf | Spleen | 6 | 40.00±5.77 |
|  | 1dpf | Intestinal tissue | 6 | 61.67±3.07 |

**Step 1. Normal distribution analysis**

**Normality**

| **Shapiro-Wilk test** | | | | |
| --- | --- | --- | --- | --- |
| **Fig** | **W** | **df** | **P** | **Sig.** |
| 1-Ha | 0.7563 | 33 | <0.0001 | ** |

****nonnormal distribution**

**Step 2. compare within PBS**

Kruskal-Wallis test

| **df** | **H** | **P** | **Sig.** |
| --- | --- | --- | --- |
| 3 | 14.88 | <0.0001 | ** |

Bonferroni's multiple comparisons test

| **Treatment** | **Comparison** | **Z** | **P** | **Sig.** |
| --- | --- | --- | --- | --- |
| PBS | Brain vs. Liver | 1.20 | >0.9999 | ns |
| PBS | Spleen vs. Liver | 3.25 | 0.007 | * |
| PBS | Intestinal tissue vs. Liver | 3.16 | 0.0094 | * |
| PBS | Spleen vs. Brain | 1.77 | 0.4577 | ns |
| PBS | Intestinal tissue vs. Brain | 1.38 | >0.9999 | ns |
| PBS | Intestinal tissue vs. Spleen | 0.67 | >0.9999 | ns |

**Step 3. compare within MIA50 μg/g**

Kruskal-Wallis test

| **df** | **H** | **P** | **Sig.** |
| --- | --- | --- | --- |
| 3 | 12.74 | <0.0001 | ** |

Bonferroni's multiple comparisons test

| **Treatment** | **Comparison** | **Z** | **P** | **Sig.** |
| --- | --- | --- | --- | --- |
| 50 μg/g | Brain vs. Liver | 0.83 | >0.9999 | ns |
| 50 μg/g | Spleen vs. Liver | 3.26 | 0.0067 | * |
| 50 μg/g | Intestinal tissue vs. Liver | 2.41 | 0.0955 | ns |
| 50 μg/g | Spleen vs. Brain | 2.43 | 0.0901 | ns |
| 50 μg/g | Intestinal tissue vs. Brain | 1.46 | 0.8704 | ns |
| 50 μg/g | Intestinal tissue vs. Spleen | 1.35 | >0.9999 | ns |

**Step 4. 50 μg/g vs. PBS**

Multiple Mann-Whitney tests with Bonferroni's correction

| **Tissue** | **Comparison** | **U** | **P** | **Sig.** |
| --- | --- | --- | --- | --- |
| Liver | 50 μg/g vs. PBS | 1.50 | 0.2381 | ns |
| Brain | 50 μg/g vs. PBS | 1.50 | >0.9999 | ns |
| Spleen | 50 μg/g vs. PBS | 3.50 | >0.9999 | ns |
| Intestinal tissue | 50 μg/g vs. PBS | 8.50 | 0.7532 | ns |

- **Fig.1Hb**

| **MIA** | | | | |
| --- | --- | --- | --- | --- |
|  | **Age** | **Tissue** | ***n*** | **M ± SEM** |
| PBS | 1dpf | Liver | 6 | 21.33 ±2.04 |
|  | 1dpf | Brain | 6 | 5.33 ±0.33 |
|  | 1dpf | Spleen | 6 | 0.00 ±0.00 |
|  | 1dpf | Intestinal tissue | 6 | 0.83 ±0.31 |
| 50μg/g | 1dpf | Liver | 6 | 34.00 ±2.00 |
|  | 1dpf | Brain | 6 | 5.33 ±0.67 |
|  | 1dpf | Spleen | 6 | 0.00 ±0.00 |
|  | 1dpf | Intestinal tissue | 6 | 1.33 ±0.33 |

**Step 1. Normal distribution analysis**

**Normality**

| **Shapiro-Wilk test** | | | | |
| --- | --- | --- | --- | --- |
| **Fig** | **W** | **df** | **P** | **Sig.** |
| 1-Hb | 0.7345 | 33 | <0.0001 | ** |

****nonnormal distribution**

**Step 2. compare within PBS**

Kruskal-Wallis test

| **df** | **H** | **P** | **Sig.** |
| --- | --- | --- | --- |
| 3 | 15.24 | <0.0001 | ** |

Bonferroni's multiple comparisons test

| **Treatment** | **Comparison** | **Z** | **P** | **Sig.** |
| --- | --- | --- | --- | --- |
| PBS | Brain vs. Liver | 1.21 | >0.9999 | ns |
| PBS | Spleen vs. Liver | 3.36 | 0.0048 | * |
| PBS | Intestinal tissue vs. Liver | 3.12 | 0.0108 | * |
| PBS | Spleen vs. Brain | 1.86 | 0.3778 | ns |
| PBS | Intestinal tissue vs. Brain | 1.34 | >0.9999 | ns |
| PBS | Intestinal tissue vs. Spleen | 0.81 | >0.9999 | ns |

**Step 3. compare within MIA50 μg/g**

Kruskal-Wallis test

| **df** | **H** | **P** | **Sig.** |
| --- | --- | --- | --- |
| 3 | 12.59 | <0.0001 | ** |

Bonferroni's multiple comparisons test

| **Treatment** | **Comparison** | **Z** | **P** | **Sig.** |
| --- | --- | --- | --- | --- |
| 50 μg/g | Brain vs. Liver | 0.83 | >0.9999 | ns |
| 50 μg/g | Spleen vs. Liver | 3.20 | 0.0083 | * |
| 50 μg/g | Intestinal tissue vs. Liver | 2.49 | 0.0769 | ns |
| 50 μg/g | Spleen vs. Brain | 2.36 | 0.1085 | ns |
| 50 μg/g | Intestinal tissue vs. Brain | 1.53 | 0.7629 | ns |
| 50 μg/g | Intestinal tissue vs. Spleen | 1.20 | >0.9999 | ns |

**Step 4. 50 μg/g vs. PBS**

Multiple Mann-Whitney tests with Bonferroni's correction

| **Tissue** | **Comparison** | **U** | **P** | **Sig.** |
| --- | --- | --- | --- | --- |
| Liver | 50 μg/g vs. PBS | 0.00 | 0.0952 | ns |
| Brain | 50 μg/g vs. PBS | 4.00 | >0.9999 | ns |
| Spleen | 50 μg/g vs. PBS | 4.50 | >0.9999 | ns |
| Intestinal tissue | 50 μg/g vs. PBS | 11.50 | >0.9999 | ns |

- **Fig.1Hc**

| **MIA** | | | | |
| --- | --- | --- | --- | --- |
|  | **Age** | **Tissue** | ***n*** | **M ± SEM** |
| PBS | 1dpf | Liver | 6 | 96.67±5.58 |
|  | 1dpf | Brain | 6 | 46.67±3.33 |
|  | 1dpf | Spleen | 6 | 16.67±3.33 |
|  | 1dpf | Intestinal tissue | 6 | 23.33±4.22 |
| 50μg/g | 1dpf | Liver | 6 | 126.67±8.82 |
|  | 1dpf | Brain | 6 | 43.33±6.67 |
|  | 1dpf | Spleen | 6 | 20.00±5.77 |
|  | 1dpf | Intestinal tissue | 6 | 96.67±5.58 |

**Step 1. Normal distribution analysis**

**Normality**

| **Shapiro-Wilk test** | | | | |
| --- | --- | --- | --- | --- |
| **Fig** | **W** | **df** | **P** | **Sig.** |
| 1-Hc | 0.8343 | 33 | 0.0002 | ** |

****nonnormal distribution**

**Step 2. compare within PBS**

Kruskal-Wallis test

| **df** | **H** | **P** | **Sig.** |
| --- | --- | --- | --- |
| 3 | 14.63 | <0.0001 | ** |

Bonferroni's multiple comparisons test

| **Treatment** | **Comparison** | **Z** | **P** | **Sig.** |
| --- | --- | --- | --- | --- |
| PBS | Brain vs. Liver | 1.25 | >0.9999 | ns |
| PBS | Spleen vs. Liver | 3.13 | 0.0104 | * |
| PBS | Intestinal tissue vs. Liver | 3.23 | 0.0073 | * |
| PBS | Spleen vs. Brain | 1.63 | 0.6213 | ns |
| PBS | Intestinal tissue vs. Brain | 1.39 | 0.9919 | ns |
| PBS | Intestinal tissue vs. Spleen | 0.49 | >0.9999 | ns |

**Step 3. compare within 50 μg/g**

Kruskal-Wallis test

| **df** | **H** | **P** | **Sig.** |
| --- | --- | --- | --- |
| 3 | 10.42 | 0.0012 | * |

Bonferroni's multiple comparisons test

| **Treatment** | **Comparison** | **Z** | **P** | **Sig.** |
| --- | --- | --- | --- | --- |
| 50 μg/g | Brain vs. Liver | 1.09 | >0.9999 | ns |
| 50 μg/g | Spleen vs. Liver | 2.79 | 0.0317 | * |
| 50 μg/g | Intestinal tissue vs. Liver | 2.68 | 0.0449 | * |
| 50 μg/g | Spleen vs. Brain | 1.70 | 0.5326 | ns |
| 50 μg/g | Intestinal tissue vs. Brain | 1.42 | 0.9348 | ns |
| 50 μg/g | Intestinal tissue vs. Spleen | 0.55 | >0.9999 | ns |

**Step 4. 50 μg/g vs. PBS**

Multiple Mann-Whitney tests with Bonferroni's correction

| **Tissue** | **Comparison** | **U** | **P** | **Sig.** |
| --- | --- | --- | --- | --- |
| Liver | 50 μg/g vs. PBS | 1.00 | 0.2381 | ns |
| Brain | 50 μg/g vs. PBS | 4.00 | >0.9999 | ns |
| Spleen | 50 μg/g vs. PBS | 3.50 | >0.9999 | ns |
| Intestinal tissue | 50 μg/g vs. PBS | 15.00 | >0.9999 | ns |

- **Figure 2**
- **Fig. 2C**

| **MIA** | | | | **PIVE** | | | |
| --- | --- | --- | --- | --- | --- | --- | --- |
|  | **Age** | ***n*** | **M±SEM** |  | **Age** | ***n*** | **M±SEM** |
| Control | 7dpf | 127 | 18.19±2.28 | E3 | 7dpf | 194 | 20.22±1.84 |
|  | 14dpf | 182 | 18.09±1.96 |  | 14dpf | 137 | 17.67±2.09 |
|  | 21dpf | 160 | 36.45±2.72 |  | 21dpf | 53 | 31.98±4.90 |
| PBS | 7dpf | 208 | 19.58±1.55 | 10μM | 7dpf | 166 | 19.11±1.56 |
|  | 14dpf | 168 | 17.84±1.84 |  | 14dpf | 156 | 21.56±1.93 |
|  | 21dpf | 128 | 33.65±2.94 |  | 21dpf | 82 | 32.97±3.42 |
| 20μg/g | 7dpf | 143 | 16.25±1.99 | 50μM | 7dpf | 145 | 15.33±1.53 |
|  | 14dpf | 144 | 21.00±1.83 |  | 14dpf | 159 | 22.52±2.03 |
|  | 21dpf | 174 | 27.59±2.11 |  | 21dpf | 92 | 32.67±3.35 |
| 50μg/g | 7dpf | 148 | 15.27±1.80 | 100μM | 7dpf | 171 | 14.13±1.57 |
|  | 14dpf | 124 | 20.59±2.25 |  | 14dpf | 119 | 17.30±1.52 |
|  | 21dpf | 120 | 18.47±2.21 |  | 21dpf | 83 | 14.90±2.40 |

**Step 1. Normal distribution analysis**

**Normality**

| **Shapiro-Wilk test** | | | | |
| --- | --- | --- | --- | --- |
| **Fig** | **W** | **df** | **P** | **Sig.** |
| 2-C | 0.8056 | 3395 | <0.0001 | ** |

****nonnormal distribution**

**Step 2. compare different ages within the same treatment**

**MIA-Control**

Kruskal-Wallis test

| **df** | **H** | **P** | **Sig.** |
| --- | --- | --- | --- |
| 2 | 25.21 | <0.0001 | ** |

Bonferroni's multiple comparisons test

| **Treatment** | **Comparison** | **Z** | **P** | **Sig.** |
| --- | --- | --- | --- | --- |
| Control | 14dpf vs. 7dpf | 0.13 | >0.9999 | ns |
| Control | 21dpf vs. 7dpf | 4.00 | 0.0002 | ** |
| Control | 21dpf vs. 14dpf | 4.55 | <0.0001 | ** |

**MIA-PBS**

Kruskal-Wallis test

| **df** | **H** | **P** | **Sig.** |
| --- | --- | --- | --- |
| 2 | 20.96 | <0.0001 | ** |

Bonferroni's multiple comparisons test

| **Treatment** | **Comparison** | **Z** | **P** | **Sig.** |
| --- | --- | --- | --- | --- |
| PBS | 14dpf vs. 7dpf | 1.36 | 0.5258 | ns |
| PBS | 21dpf vs. 7dpf | 3.42 | 0.0019 | * |
| PBS | 21dpf vs. 14dpf | 4.48 | <0.0001 | ** |

**MIA-20μg/g**

Kruskal-Wallis test

| **df** | **H** | **P** | **Sig.** |
| --- | --- | --- | --- |
| 2 | 12.57 | 0.0019 | * |

Bonferroni's multiple comparisons test

| **Treatment** | **Comparison** | **Z** | **P** | **Sig.** |
| --- | --- | --- | --- | --- |
| 20μg/g | 14dpf vs. 7dpf | 1.954 | 0.1519 | ns |
| 20μg/g | 21dpf vs. 7dpf | 3.544 | 0.0012 | * |
| 20μg/g | 21dpf vs. 14dpf | 1.503 | 0.3989 | ns |

**MIA-50μg/g**

Kruskal-Wallis test

| **df** | **H** | **P** | **Sig.** |
| --- | --- | --- | --- |
| 2 | 4.391 | 0.1113 | ns |

**PIVE-E3**

Kruskal-Wallis test

| **df** | **H** | **P** | **Sig.** |
| --- | --- | --- | --- |
| 2 | 3.392 | 0.1834 | ns |

**PIVE-10μM**

Kruskal-Wallis test

| **df** | **H** | **P** | **Sig.** |
| --- | --- | --- | --- |
| 2 | 8.502 | 0.00143 | * |

Bonferroni's multiple comparisons test

| **Treatment** | **Comparison** | **Z** | **P** | **Sig.** |
| --- | --- | --- | --- | --- |
| 10μM | 14dpf vs. 7dpf | 0.2129 | >0.9999 | ns |
| 10μM | 21dpf vs. 7dpf | 2.750 | 0.0179 | * |
| 10μM | 21dpf vs. 14dpf | 2.547 | 0.0326 | * |

**PIVE-50μM**

Kruskal-Wallis test

| **df** | **H** | **P** | **Sig.** |
| --- | --- | --- | --- |
| 2 | 11.86 | 0.0027 | * |

Bonferroni's multiple comparisons test

| **Treatment** | **Comparison** | **Z** | **P** | **Sig.** |
| --- | --- | --- | --- | --- |
| 50μM | 14dpf vs. 7dpf | 1.649 | 0.2974 | ns |
| 50μM | 21dpf vs. 7dpf | 3.442 | 0.0017 | * |
| 50μM | 21dpf vs. 14dpf | 2.057 | 0.1192 | ns |

**PIVE-100μM**

Kruskal-Wallis test

| **df** | **H** | **P** | **Sig.** |
| --- | --- | --- | --- |
| 2 | 11.41 | 0.0033 | * |

Bonferroni's multiple comparisons test

| **Treatment** | **Comparison** | **Z** | **P** | **Sig.** |
| --- | --- | --- | --- | --- |
| 100μM | 14dpf vs. 7dpf | 3.144 | 0.0050 | * |
| 100μM | 21dpf vs. 7dpf | 0.02550 | >0.9999 | ns |
| 100μM | 21dpf vs. 14dpf | 2.600 | 0.0279 | * |

**Step 3. compare different treatments within the same age**

**MIA-7dpf**

Kruskal-Wallis test

| **df** | **H** | **P** | **Sig.** |
| --- | --- | --- | --- |
| 3 | 7.616 | 0.0547 | ns |

**MIA-14dpf**

Kruskal-Wallis test

| **df** | **H** | **P** | **Sig.** |
| --- | --- | --- | --- |
| 3 | 6.535 | 0.0883 | ns |

**MIA-21dpf**

Kruskal-Wallis test

| **df** | **H** | **P** | **Sig.** |
| --- | --- | --- | --- |
| 3 | 8.486 | 0.0370 | * |

Bonferroni's multiple comparisons test

| **Age** | **Comparison** | **Z** | **P** | **Sig.** |
| --- | --- | --- | --- | --- |
| 21dpf | PBS vs. Control | 0.18 | >0.9999 | ns |
| 21dpf | MIA-20μg/g vs. Control | 1.76 | 0.4723 | ns |
| 21dpf | MIA-50μg/g vs. Control | 4.16 | 0.0002 | ## |
| 21dpf | MIA-20μg/g vs. PBS | 1.806 | 0.4256 | ns |
| 21dpf | MIA-50μg/g vs. PBS | 4.056 | 0.0003 | $$ |
| 21dpf | MIA-50μg/g vs. MIA-20μg/g | 2.571 | 0.0609 | ns |

**PIVE-7dpf**

Kruskal-Wallis test

| **df** | **H** | **P** | **Sig.** |
| --- | --- | --- | --- |
| 3 | 11.53 | 0.0092 | * |

Bonferroni's multiple comparisons test

| **Age** | **Comparison** | **Z** | **P** | **Sig.** |
| --- | --- | --- | --- | --- |
| 7dpf | PIVE-10 μM vs. E3 | 1.33 | 0.5521 | ns |
| 7dpf | PIVE-50 μm vs. E3 | 0.17 | >0.9999 | ns |
| 7dpf | PIVE-100 μM vs. E3 | 2.15 | 0.0951 | ns |
| 7dpf | PIVE-50 μm vs. PIVE-10 μM | 1.397 | 0.9746 | ns |
| 7dpf | PIVE-100 μM vs. PIVE-10 μM | 3.357 | 0.0047 | § |
| 7dpf | PIVE-100 μM vs. PIVE-50 μM | 1.833 | 0.4004 | ns |

**PIVE-14dpf**

Kruskal-Wallis test

| **df** | **H** | **P** | **Sig.** |
| --- | --- | --- | --- |
| 3 | 6.291 | 0.0983 | ns |

**PIVE-21dpf**

Kruskal-Wallis test

| **df** | **H** | **P** | **Sig.** |
| --- | --- | --- | --- |
| 3 | 17.86 | 0.0005 | ** |

Bonferroni's multiple comparisons test

| **Age** | **Comparison** | **Z** | **P** | **Sig.** |
| --- | --- | --- | --- | --- |
| 21dpf | PIVE-10 μM vs. E3 | 0.6633 | >0.9999 | ns |
| 21dpf | PIVE-50 Μm vs. E3 | 0.7241 | >0.9999 | ns |
| 21dpf | PIVE-100 μM vs. E3 | 2.542 | 0.0662 | ns |
| 21dpf | PIVE-50 μm vs. PIVE-10 μM | 0.05240 | >0.9999 | ns |
| 21dpf | PIVE-100 μM vs. PIVE-10 μM | 3.600 | 0.0019 | § |
| 21dpf | PIVE-100 μM vs. PIVE-50 μM | 3.751 | 0.0011 | £ |

**Step 4. MIA vs. PIVE in same age**

MIA-20μg/g vs. PIVE-10/50/100μM

Multiple Mann-Whitney tests with Bonferroni's correction

| **Age** | **Comparison** | **U** | **P** | **Sig.** |
| --- | --- | --- | --- | --- |
| 7dpf | 10 μM vs. 20 μg/g | 9742 | 0.017699 | **%** |
| 14dpf | 10 μM vs. 20 μg/g | 11172 | >0.999999 | ns |
| 21dpf | 10 μM vs. 20 μg/g | 6537 | 0.275130 | ns |
| 7dpf | 50 μM vs. 20 μg/g | 9191 | 0.274526 | ns |
| 14dpf | 50 μM vs. 20 μg/g | 11175 | >0.999999 | ns |
| 21dpf | 50 μM vs. 20 μg/g | 7298 | 0.232951 | ns |
| 7dpf | 100 μM vs. 20 μg/g | 11861 | >0.999999 | ns |
| 14dpf | 100 μM vs. 20 μg/g | 8290 | >0.999999 | ns |
| 21dpf | 100 μM vs. 20 μg/g | 5025 | 0.002215 | **%** |

MIA-50μg/g vs. PIVE-10/50/100μM

Multiple Mann-Whitney tests with Bonferroni's correction

| **Age** | **Comparison** | **U** | **P** | **Sig.** |
| --- | --- | --- | --- | --- |
| 7dpf | 10 μM vs. 50 μg/g | 9916 | 0.008084 | @ |
| 14dpf | 10 μM vs. 50 μg/g | 9479 | >0.999999 | ns |
| 21dpf | 10 μM vs. 50 μg/g | 3658 | 0.004586 | @ |
| 7dpf | 50 μM vs. 50 μg/g | 9415 | 0.195304 | ns |
| 14dpf | 50 μM vs. 50 μg/g | 9265 | >0.999999 | ns |
| 21dpf | 50 μM vs. 50 μg/g | 4032 | 0.001776 | @ |
| 7dpf | 100 μM vs. 50 μg/g | 12542 | >0.999999 | ns |
| 14dpf | 100 μM vs. 50 μg/g | 7091 | >0.999999 | ns |
| 21dpf | 100 μM vs. 50 μg/g | 4376 | >0.999999 | ns |

- **Fig. 2E**

| **MIA** | | | | **PIVE** | | | |
| --- | --- | --- | --- | --- | --- | --- | --- |
|  | **Age** | ***n*** | **M ± SEM** |  | **Age** | ***n*** | **M ± SEM** |
| Control | 1mpf | 43 | 48.52±1.86 | E3 | 1mpf | 38 | 53.15±2.89 |
|  | 2mpf | 21 | 46.90±3.72 |  | 2mpf | 38 | 57.31±3.43 |
|  | 3mpf | 26 | 43.84±3.08 |  | 3mpf | 33 | 43.05±2.75 |
| PBS | 1mpf | 43 | 52.07±2.73 | 10μM | 1mpf | 32 | 47.77±3.72 |
|  | 2mpf | 28 | 45.19±3.66 |  | 2mpf | 27 | 53.42±4.20 |
|  | 3mpf | 34 | 40.94±3.45 |  | 3mpf | 24 | 48.94±3.32 |
| 20μg/g | 1mpf | 48 | 48.52±2.12 | 50μM | 1mpf | 37 | 50.21±3.23 |
|  | 2mpf | 34 | 54.26±3.65 |  | 2mpf | 36 | 52.18±3.19 |
|  | 3mpf | 21 | 52.33±2.86 |  | 3mpf | 40 | 48.31±3.13 |
| 50μg/g | 1mpf | 45 | 37.92±1.83 | 100μM | 1mpf | 36 | 37.67±3.20 |
|  | 2mpf | 36 | 51.86±2.64 |  | 2mpf | 25 | 37.32±2.60 |
|  | 3mpf | 29 | 55.28±3.86 |  | 3mpf | 25 | 49.70±4.67 |

**Step 1. Normal distribution analysis**

**Normality**

| **Shapiro-Wilk test** | | | | |
| --- | --- | --- | --- | --- |
| **Fig** | **W** | **df** | **P** | **Sig.** |
| 2-E | 0.9787 | 799 | <0.0001 | ** |

****nonnormal distribution**

**Step 2. compare different ages within the same treatment**

**MIA-Control**

Kruskal-Wallis test

| **df** | **H** | **P** | **Sig.** |
| --- | --- | --- | --- |
| 2 | 4.107 | 0.1283 | ns |

**MIA-PBS**

Kruskal-Wallis test

| **df** | **H** | **P** | **Sig.** |
| --- | --- | --- | --- |
| 2 | 8.574 | 0.0137 | * |

Bonferroni's multiple comparisons test

| **Treatment** | **Comparison** | **Z** | **P** | **Sig.** |
| --- | --- | --- | --- | --- |
| PBS | 2mpf vs. 1mpf | 1.75 | 0.2412 | ns |
| PBS | 3mpf vs. 1mpf | 2.86 | 0.0125 | * |
| PBS | 3mpf vs. 2mpf | 0.91 | >0.9999 | ns |

**MIA-20μg/g**

Kruskal-Wallis test

| **df** | **H** | **P** | **Sig.** |
| --- | --- | --- | --- |
| 2 | 1.331 | 0.5141 | ns |

**MIA-50μg/g**

Kruskal-Wallis test

| **df** | **H** | **P** | **Sig.** |
| --- | --- | --- | --- |
| 2 | 21.99 | <0.0001 | * |

Bonferroni's multiple comparisons test

| **Treatment** | **Comparison** | **Z** | **P** | **Sig.** |
| --- | --- | --- | --- | --- |
| **50μg/g** | 2mpf vs. 1mpf | 3.782 | 0.0005 | ** |
| **50μg/g** | 3mpf vs. 1mpf | 4.097 | 0.0001 | ** |
| **50μg/g** | 3mpf vs. 2mpf | 0.5204 | >0.9999 | ns |

**PIVE-E3**

Kruskal-Wallis test

| **df** | **H** | **P** | **Sig.** |
| --- | --- | --- | --- |
| 2 | 9.892 | 0.0071 | ns |

Bonferroni's multiple comparisons test

| **Treatment** | **Comparison** | **Z** | **P** | **Sig.** |
| --- | --- | --- | --- | --- |
| E3 | 2mpf vs. 1mpf | 0.73 | >0.9999 | ns |
| E3 | 3mpf vs. 1mpf | 2.33 | 0.0592 | ns |
| E3 | 3mpf vs. 2mpf | 3.03 | 0.0073 | * |

**PIVE-10μM**

Kruskal-Wallis test

| **df** | **H** | **P** | **Sig.** |
| --- | --- | --- | --- |
| 2 | 1.456 | 0.4828 | ns |

**PIVE-50μM**

Kruskal-Wallis test

| **df** | **H** | **P** | **Sig.** |
| --- | --- | --- | --- |
| 2 | 0.7867 | 0.6748 | ns |

**PIVE-100μM**

Kruskal-Wallis test

| **df** | **H** | **P** | **Sig.** |
| --- | --- | --- | --- |
| 2 | 6.440 | 0.0400 | * |

Bonferroni's multiple comparisons test

| **Treatment** | **Comparison** | **Z** | **P** | **Sig.** |
| --- | --- | --- | --- | --- |
| 100μM | 2mpf vs. 1mpf | 0.3347 | >0.9999 | ns |
| 100μM | 3mpf vs. 1mpf | 2.157 | 0.0929 | ns |
| 100μM | 3mpf vs. 2mpf | 2.294 | 0.0654 | ns |

**Step 3. compare different treatments within the same age**

**MIA-1mpf**

Kruskal-Wallis test

| **df** | **H** | **P** | **Sig.** |
| --- | --- | --- | --- |
| 3 | 20.65 | 0.0001 | ** |

Bonferroni's multiple comparisons test

| **Age** | **Comparison** | **Z** | **P** | **Sig.** |
| --- | --- | --- | --- | --- |
| 1mpf | PBS vs. Control | 0.39 | >0.9999 | ns |
| 1mpf | 20 μg/g vs. Control | 0.24 | >0.9999 | ns |
| 1mpf | 50 μg/g vs. Control | 3.59 | 0.002 | # |
| 1mpf | 20 μg/g vs. PBS | 0.6363 | >0.9999 | ns |
| 1mpf | 50 μg/g vs. PBS | 3.985 | 0.0004 | $$ |
| 1mpf | 50 μg/g vs. 20 μg/g | 3.451 | 0.0033 | % |

**MIA-2mpf**

Kruskal-Wallis test

| **df** | **H** | **P** | **Sig.** |
| --- | --- | --- | --- |
| 3 | 6.877 | 0.0759 | ns |

**MIA-3mpf**

Kruskal-Wallis test

| **df** | **H** | **P** | **Sig.** |
| --- | --- | --- | --- |
| 3 | 13.78 | 0.0032 | * |

Bonferroni's multiple comparisons test

| **Age** | **Comparison** | **Z** | **P** | **Sig.** |
| --- | --- | --- | --- | --- |
| 3mpf | Control vs. PBS | 0.64 | >0.9999 | ns |
| 3mpf | 20 μg/g vs. Control | 1.93 | 0.3193 | ns |
| 3mpf | 50 μg/g vs. Control | 2.34 | 0.1167 | ns |
| 3mpf | 20 μg/g vs. PBS | 2.645 | 0.049 | ns |
| 3mpf | 50 μg/g vs. PBS | 3.157 | 0.0096 | $ |
| 3mpf | 50 μg/g vs. 20 μg/g | 0.2233 | >0.9999 | ns |

**PIVE-1mpf**

Kruskal-Wallis test

| **df** | **H** | **P** | **Sig.** |
| --- | --- | --- | --- |
| 3 | 11.78 | 0.0082 | * |

Bonferroni's multiple comparisons test

| **Age** | **Comparison** | **Z** | **P** | **Sig.** |
| --- | --- | --- | --- | --- |
| 1mpf | 10 μM vs. E3 | 1.67 | 0.2832 | ns |
| 1mpf | 50 μM vs. E3 | 0.79 | >0.9999 | ns |
| 1mpf | 100 μM vs. E3 | 3.27 | 0.0032 | ns |
| 1mpf | 50 μM vs. 10 μM | 0.9112 | >0.9999 | ns |
| 1mpf | 100 μM vs. 10 μM | 1.478 | 0.8370 | ns |
| 1mpf | 100 μM vs. 50 μM | 2.473 | 0.0804 | ns |

**PIVE-2mpf**

Kruskal-Wallis test

| **df** | **H** | **P** | **Sig.** |
| --- | --- | --- | --- |
| 3 | 15.1 | 0.0017 | * |

Bonferroni's multiple comparisons test

| **Age** | **Comparison** | **Z** | **P** | **Sig.** |
| --- | --- | --- | --- | --- |
| 2mpf | 10 μM vs. E3 | 0.73 | >0.9999 | ns |
| 2mpf | 50 μM vs. E3 | 0.85 | >0.9999 | ns |
| 2mpf | 100 μM vs. E3 | 3.74 | 0.0006 | †† |
| 2mpf | 50 μM vs. 10 μM | 0.06175 | >0.9999 | ns |
| 2mpf | 100 μM vs. 10 μM | 2.808 | 0.0299 | § |
| 2mpf | 100 μM vs. 50 μM | 2.933 | 0.0202 | £ |

**PIVE-3mpf**

Kruskal-Wallis test

| **df** | **H** | **P** | **Sig.** |
| --- | --- | --- | --- |
| 3 | 2.184 | 0.5352 | ns |

**Step 4. MIA vs. PIVE in same age**

MIA-20μg/g vs. PIVE-10/50/100μM

Multiple Mann-Whitney tests with Bonferroni's correction

| **Age** | **Comparison** | **U** | **P** | **Sig.** |
| --- | --- | --- | --- | --- |
| 1mpf | 10 μM vs. 20 μg/g | 653.0 | 0.787410 | ns |
| 2mpf | 10 μM vs. 20 μg/g | 447.0 | >0.999999 | ns |
| 3mpf | 10 μM vs. 20 μg/g | 218.0 | >0.999999 | ns |
| 1mpf | 50 μM vs. 20 μg/g | 848.0 | >0.999999 | ns |
| 2mpf | 50 μM vs. 20 μg/g | 589.0 | >0.999999 | ns |
| 3mpf | 50 μM vs. 20 μg/g | 353.0 | 0.946564 | ns |
| 1mpf | 100 μM vs. 20 μg/g | 530.0 | 0.006798 | % |
| 2mpf | 100 μM vs. 20 μg/g | 212.0 | 0.002575 | % |
| 3mpf | 100 μM vs. 20 μg/g | 235.0 | >0.999999 | ns |

MIA-50μg/g vs. PIVE-10/50/100μM

Multiple Mann-Whitney tests with Bonferroni's correction

| **Age** | **Comparison** | **U** | **P** | **Sig.** |
| --- | --- | --- | --- | --- |
| 1mpf | 10 μM vs. 50 μg/g | 561.0 | 0.304757 | ns |
| 2mpf | 10 μM vs. 50 μg/g | 472.5 | >0.999999 | ns |
| 3mpf | 10 μM vs. 50 μg/g | 269.0 | 0.485973 | ns |
| 1mpf | 50 μM vs. 50 μg/g | 526.5 | 0.012003 | @ |
| 2mpf | 50 μM vs. 50 μg/g | 641.5 | >0.999999 | ns |
| 3mpf | 50 μM vs. 50 μg/g | 448.0 | 0.330832 | ns |
| 1mpf | 100 μM vs. 50 μg/g | 808.0 | >0.999999 | ns |
| 2mpf | 100 μM vs. 50 μg/g | 210.0 | 0.000922 | @@ |
| 3mpf | 100 μM vs. 50 μg/g | 307.0 | >0.999999 | ns |

- **Figure 3**
- **Fig. 3B**

| **MIA** | | | | **PIVE** | | | |
| --- | --- | --- | --- | --- | --- | --- | --- |
|  | **Age** | ***n*** | **Media [IQ3, IQ1]** |  | **Age** | ***n*** | **Media [IQ3, IQ1]** |
| Control | 7dpf | 76 | 3.96[4.36, 3.48] | E3 | 7dpf | 104 | 3.99[4.49, 3.41] |
|  | 14dpf | 64 | 3.91[4.41, 2.91] |  | 14dpf | 84 | 4.09[4.58, 3.76] |
|  | 21dpf | 48 | 3.25[4.02, 2.97] |  | 21dpf | 108 | 3.83[4.42, 3.24] |
| PBS | 7dpf | 64 | 3.81[4.36, 3.02] | 10μM | 7dpf | 88 | 3.55[4.03, 2.79] |
|  | 14dpf | 108 | 3.82[4.25, 3.31] |  | 14dpf | 92 | 4.09[4.40, 3.61] |
|  | 21dpf | 56 | 3.76[4.12, 3.25] |  | 21dpf | 152 | 4.00[4.40, 3.44] |
| 20μg/g | 7dpf | 72 | 4.49[4.89, 4.01] | 50μM | 7dpf | 92 | 3.67[4.11, 2.91] |
|  | 14dpf | 76 | 4.50[4.88, 4.05] |  | 14dpf | 84 | 4.22[4.54, 3.81] |
|  | 21dpf | 64 | 4.06[4.47, 3.65] |  | 21dpf | 120 | 3.87[4.19, 3.48] |
| 50μg/g | 7dpf | 60 | 4.17[4.69, 3.75] | 100μM | 7dpf | 92 | 3.70[4.28, 3.09] |
|  | 14dpf | 124 | 4.12[4.54, 3.60] |  | 14dpf | 136 | 4.14[4.47, 3.67] |
|  | 21dpf | 60 | 4.13[4.62, 3.63] |  | 21dpf | 168 | 3.87[4.35, 3.31] |

**Step 1. Normal distribution analysis**

**Normality**

| **Shapiro-Wilk test** | | | | |
| --- | --- | --- | --- | --- |
| **Fig** | **W** | **df** | **P** | **Sig.** |
| 3-B | 0.988 | 3216 | <0.0001 | ****** |

****nonnormal distribution**

**Step 2. compare different ages within the same treatment**

**MIA-Control**

Kruskal-Wallis test

| **df** | **H** | **P** | **Sig.** |
| --- | --- | --- | --- |
| 2 | 18.22 | 0.0001 | ** |

Bonferroni's multiple comparisons test

| **Treatment** | **Comparison** | **Z** | **P** | **Sig.** |
| --- | --- | --- | --- | --- |
| Control | 14dpf vs. 7dpf | 1.47 | 0.4289 | ns |
| Control | 21dpf vs. 7dpf | 4.25 | <0.0001 | ** |
| Control | 21dpf vs. 14dpf | 2.81 | 0.0151 | * |

**MIA-PBS**

Kruskal-Wallis test

| **df** | **H** | **P** | **Sig.** |
| --- | --- | --- | --- |
| 2 | 1.054 | 0.5903 | ns |

**MIA-20μg/g**

Kruskal-Wallis test

| **df** | **H** | **P** | **Sig.** |
| --- | --- | --- | --- |
| 2 | 25.55 | <0.0001 | ** |

Bonferroni's multiple comparisons test

| **Treatment** | **Comparison** | **Z** | **P** | **Sig.** |
| --- | --- | --- | --- | --- |
| 20μg/g | 14dpf vs. 7dpf | 0.3635 | >0.9999 | ns |
| 20μg/g | 21dpf vs. 7dpf | 4.585 | <0.0001 | ** |
| 20μg/g | 21dpf vs. 14dpf | 4.233 | <0.0001 | ** |

**MIA-50μg/g**

Kruskal-Wallis test

| **df** | **H** | **P** | **Sig.** |
| --- | --- | --- | --- |
| 2 | 0.5090 | 0.7753 | ns |

**PIVE-E3**

Kruskal-Wallis test

| **df** | **H** | **P** | **Sig.** |
| --- | --- | --- | --- |
| 2 | 10.23 | 0.0060 | * |

Bonferroni's multiple comparisons test

| **Treatment** | **Comparison** | **Z** | **P** | **Sig.** |
| --- | --- | --- | --- | --- |
| E3 | 14dpf vs. 7dpf | 1.89 | 0.1765 | ns |
| E3 | 21dpf vs. 7dpf | 1.37 | 0.5159 | ns |
| E3 | 21dpf vs. 14dpf | 3.20 | 0.0042 | * |

**PIVE-10μM**

Kruskal-Wallis test

| **df** | **H** | **P** | **Sig.** |
| --- | --- | --- | --- |
| 2 | 32.87 | <0.0001 | ** |

Bonferroni's multiple comparisons test

| **Treatment** | **Comparison** | **Z** | **P** | **Sig.** |
| --- | --- | --- | --- | --- |
| 10μM | 14dpf vs. 7dpf | 5.172 | <0.0001 | ** |
| 10μM | 21dpf vs. 7dpf | 4.978 | <0.0001 | ** |
| 10μM | 21dpf vs. 14dpf | 0.7908 | >0.9999 | ns |

**PIVE-50μM**

Kruskal-Wallis test

| **df** | **H** | **P** | **Sig.** |
| --- | --- | --- | --- |
| 2 | 42.88 | <0.0001 | ** |

Bonferroni's multiple comparisons test

| **Treatment** | **Comparison** | **Z** | **P** | **Sig.** |
| --- | --- | --- | --- | --- |
| 50μM | 14dpf vs. 7dpf | 6.071 | <0.0001 | ** |
| 50μM | 21dpf vs. 7dpf | 4.847 | <0.0001 | ** |
| 50μM | 21dpf vs. 14dpf | 0.6624 | >0.9999 | ns |

**PIVE-100μM**

Kruskal-Wallis test

| **df** | **H** | **P** | **Sig.** |
| --- | --- | --- | --- |
| 2 | 42.88 | <0.0001 | ** |

Bonferroni's multiple comparisons test

| **Treatment** | **Comparison** | **Z** | **P** | **Sig.** |
| --- | --- | --- | --- | --- |
| 100μM | 14dpf vs. 7dpf | 4.511 | <0.0001 | ** |
| 100μM | 21dpf vs. 7dpf | 1.659 | 0.2913 | ns |
| 100μM | 21dpf vs. 14dpf | 3.413 | 0.0019 | * |

**Step 3. compare different treatments within the same age**

**MIA-7dpf**

Kruskal-Wallis test

| **df** | **H** | **P** | **Sig.** |
| --- | --- | --- | --- |
| 3 | 53.24 | <0.0001 | ** |

Bonferroni's multiple comparisons test

| **Age** | **Comparison** | **Z** | **P** | **Sig.** |
| --- | --- | --- | --- | --- |
| 7dpf | PBS vs. Control | 1.26 | >0.9999 | ns |
| 7dpf | MIA-20μg/g vs. Control | 5.66 | <0.0001 | **##** |
| 7dpf | MIA-50μg/g vs. Control | 2.54 | 0.0665 | **ns** |
| 7dpf | MIA-20μg/g vs. PBS | 6.658 | <0.0001 | **$$** |
| 7dpf  7dpf | MIA-50μg/g vs. PBS  MIA-50μg/g vs. MIA-20μg/g | 3.696  3.225 | 0.0013  0.0075 | **$**  **%** |

**MIA-14dpf**

Kruskal-Wallis test

| **df** | **H** | **P** | **Sig.** |
| --- | --- | --- | --- |
| 3 | 64.87 | <0.0001 | ** |

Bonferroni's multiple comparisons test

| **Age** | **Comparison** | **Z** | **P** | **Sig.** |
| --- | --- | --- | --- | --- |
| 14dpf | PBS vs. Control | 0.31 | >0.9999 | ns |
| 14dpf | MIA-20μg/g vs. Control | 6.31 | <0.0001 | **##** |
| 14dpf | MIA-50μg/g vs. Control | 2.68 | 0.0444 | **#** |
| 14dpf | MIA-20μg/g vs. PBS | 7.452 | <0.0001 | **$$** |
| 14dpf  14dpf | MIA-50μg/g vs. PBS  MIA-50μg/g vs. MIA-20μg/g | 3.295  3.451 | 0.0059  0.0033 | **$**  **%** |

**MIA-21dpf**

Kruskal-Wallis test

| **df** | **H** | **P** | **Sig.** |
| --- | --- | --- | --- |
| 3 | 49.11 | <0.0001 | ** |

Bonferroni's multiple comparisons test

| **Age** | **Comparison** | **Z** | **P** | **Sig.** |
| --- | --- | --- | --- | --- |
| 21dpf | PBS vs. Control | 2.07 | 0.2316 | ns |
| 21dpf | MIA-20μg/g vs. Control | 5.57 | <0.0001 | **##** |
| 21dpf | MIA-50μg/g vs. Control | 5.95 | <0.0001 | **##** |
| 21dpf | MIA-20μg/g vs. PBS | 3.593 | 0.002 | **$** |
| 21dpf  21dpf | MIA-50μg/g vs. PBS  MIA-50μg/g vs. MIA-20μg/g | 4.013  0.4906 | 0.0004  >0.9999 | **$$**  ns |

**PIVE-7dpf**

Kruskal-Wallis test

| **df** | **H** | **P** | **Sig.** |
| --- | --- | --- | --- |
| 3 | 25.19 | <0.0001 | ** |

Bonferroni's multiple comparisons test

| **Age** | **Comparison** | **Z** | **P** | **Sig.** |
| --- | --- | --- | --- | --- |
| 7dpf | PIVE-10 μM vs. E3 | 4.79 | <0.0001 | **††** |
| 7dpf | PIVE-50 μM vs. E3 | 3.56 | 0.0022 | **†** |
| 7dpf | PIVE-100 μM vs. E3 | 2.64 | 0.0494 | **†** |
| 7dpf  7dpf  7dpf | PIVE-50 μm vs. PIVE-10 μM  PIVE-100 μM vs.PIVE-10 μM  PIVE-100 μM vs.PIVE-50 μM | 1.234  2.113  0.8891 | >0.9999  0.2077  >0.9999 | ns  ns  ns |

**PIVE-14dpf**

Kruskal-Wallis test

| **df** | **H** | **P** | **Sig.** |
| --- | --- | --- | --- |
| 3 | 5.848 | 0.1192 | ns |

**PIVE-21dpf**

Kruskal-Wallis test

| **df** | **H** | **P** | **Sig.** |
| --- | --- | --- | --- |
| 3 | 4.329 | 0.2280 | ns |

**Step 4. MIA vs. PIVE in same age**

**MIA-20μg/g vs. PIVE-10/50/100μM**

Multiple Mann-Whitney tests with Bonferroni's correction

| **Age** | **Comparison** | **U** | **P** | **Sig.** |
| --- | --- | --- | --- | --- |
| 7dpf | 10μM vs. 20μg/g | 2594 | <0.0001 | %% |
| 14dpf | 10μM vs. 20μg/g | 4524 | <0.0001 | %% |
| 21dpf | 10μM vs. 20μg/g | 9846 | 0.1543 | ns |
| 7dpf | 50μM vs. 20μg/g | 2906 | <0.0001 | %% |
| 14dpf | 50μM vs. 20μg/g | 5067 | 0.0023 | % |
| 21dpf | 50μM vs. 20μg/g | 6609 | 0.0012 | % |
| 7dpf | 100μM vs. 20μg/g | 3425 | <0.0001 | %% |
| 14dpf | 100μM vs. 20μg/g | 7176 | 0.0001 | %% |
| 21dpf | 100μM vs. 20μg/g | 9897 | 0.0086 | % |

**MIA-50μg/g vs. PIVE-10/50/100μM**

Multiple Mann-Whitney tests with Bonferroni's correction

| **Age** | **Comparison** | **U** | **P** | **Sig.** |
| --- | --- | --- | --- | --- |
| 7dpf | 10μM vs. 50μg/g | 4267 | <0.0001 | @@ |
| 14dpf | 10μM vs. 50μg/g | 5717 | 0.3124 | ns |
| 21dpf | 10μM vs. 50μg/g | 8833 | 0.0533 | ns |
| 7dpf | 50μM vs. 50μg/g | 4896 | <0.0001 | @@ |
| 14dpf | 50μM vs. 50μg/g | 5216 | 0.3173 | ns |
| 21dpf | 50μM vs. 50μg/g | 6096 | 0.0009 | @@ |
| 7dpf | 100μM vs. 50μg/g | 5490 | 0.0009 | @@ |
| 14dpf | 100μM vs. 50μg/g | 9046 | 0.8428 | ns |
| 21dpf | 100μM vs. 50μg/g | 8895 | 0.0023 | @ |

- **Fig. 3C**

| **MIA** | | | | **PIVE** | | | |
| --- | --- | --- | --- | --- | --- | --- | --- |
| **Group** | **Age** | ***n*** | **Media [IQ3, IQ1]** | **Group** | **Age** | ***n*** | **Media [IQ3, IQ1]** |
| Control | 1mpf | 72 | 9.58[10.72, 8.10] | E3 | 1mpf | 108 | 9.49[10.87, 7.97] |
|  | 2mpf | 80 | 9.50[10.71, 8.63] |  | 2mpf | 124 | 10.40[11.40, 9.16] |
|  | 3mpf | 104 | 11.17[12.70, 9.11] |  | 3mpf | 92 | 10.05[10.96, 8.66] |
| PBS | 1mpf | 96 | 9.37[10.29, 7.62] | 10μM | 1mpf | 140 | 10.01[11.49, 8.89] |
|  | 2mpf | 76 | 9.30[10.33, 8.05] |  | 2mpf | 96 | 9.04[10.16, 8.11] |
|  | 3mpf | 108 | 11.01[12.28, 9.74] |  | 3mpf | 92 | 10.29[11.55, 8.93] |
| 20μg/g | 1mpf | 56 | 11.93[13.88, 9.73] | 50μM | 1mpf | 76 | 10.87[12.04, 9.10] |
|  | 2mpf | 80 | 12.74[13.87, 11.92] |  | 2mpf | 108 | 10.40[12.01, 8.44] |
|  | 3mpf | 100 | 12.52[13.77, 11.41] |  | 3mpf | 84 | 11.06[12.35, 8.98] |
| 50μg/g | 1mpf | 60 | 9.73[11.56, 8.50] | 100μM | 1mpf | 216 | 10.47[13.03, 7.58] |
|  | 2mpf | 80 | 11.04[11.97, 9.54] |  | 2mpf | 220 | 9.46[11.16, 7.00] |
|  | 3mpf | 84 | 11.97[14.84, 9.96] |  | 3mpf | 116 | 10.48[12.10, 8.53] |

**Step 1. Normal distribution analysis**

**Normality**

| **Shapiro-Wilk test** | | | | |
| --- | --- | --- | --- | --- |
| **Fig** | **W** | **df** | **P** | **Sig.** |
| 3-B | 0.995 | 3566 | <0.0001 | ** |

****nonnormal distribution**

**Step 2. compare different ages within the same treatment**

**MIA-Control**

Kruskal-Wallis test

| **df** | **H** | **P** | **Sig.** |
| --- | --- | --- | --- |
| 2 | 28.23 | <0.0001 | ** |

Bonferroni's multiple comparisons test

| **Treatment** | **Comparison** | **Z** | **P** | **Sig.** |
| --- | --- | --- | --- | --- |
| Control | 2mpf vs. 1mpf | 0.09314 | >0.9999 | ns |
| Control | 3mpf vs. 1mpf | 4.507 | <0.0001 | ** |
| Control | 3mpf vs. 2mpf | 4.474 | <0.0001 | ** |

**MIA-PBS**

Kruskal-Wallis test

| **df** | **H** | **P** | **Sig.** |
| --- | --- | --- | --- |
| 2 | 80.23 | <0.0001 | ** |

Bonferroni's multiple comparisons test

| **Treatment** | **Comparison** | **Z** | **P** | **Sig.** |
| --- | --- | --- | --- | --- |
| PBS | 2mpf vs. 1mpf | 0.8008 | >0.9999 | ns |
| PBS | 3mpf vs. 1mpf | 8.196 | <0.0001 | ** |
| PBS | 3mpf vs. 2mpf | 6.857 | <0.0001 | ** |

**MIA-20μg/g**

Kruskal-Wallis test

| **df** | **H** | **P** | **Sig.** |
| --- | --- | --- | --- |
| 2 | 9.984 | 0.0068 | * |

Bonferroni's multiple comparisons test

| **Treatment** | **Comparison** | **Z** | **P** | **Sig.** |
| --- | --- | --- | --- | --- |
| 20μg/g | 2mpf vs. 1mpf | 3.146 | 0.0050 | * |
| 20μg/g | 3mpf vs. 1mpf | 2.163 | 0.0915 | ns |
| 20μg/g | 3mpf vs. 2mpf | 1.247 | 0.6370 | ns |

**MIA-50μg/g**

Kruskal-Wallis test

| **df** | **H** | **P** | **Sig.** |
| --- | --- | --- | --- |
| 2 | 27.94 | <0.0001 | * |

Bonferroni's multiple comparisons test

| **Treatment** | **Comparison** | **Z** | **P** | **Sig.** |
| --- | --- | --- | --- | --- |
| 50μg/g | 2mpf vs. 1mpf | 2.052 | 0.1205 | ns |
| 50μg/g | 3mpf vs. 1mpf | 5.162 | <0.0001 | ** |
| 50μg/g | 3mpf vs. 2mpf | 3.342 | 0.0025 | * |

**PIVE-E3**

Kruskal-Wallis test

| **df** | **H** | **P** | **Sig.** |
| --- | --- | --- | --- |
| 2 | 6.854 | 0.0325 | * |

Bonferroni's multiple comparisons test

| **Treatment** | **Comparison** | **Z** | **P** | **Sig.** |
| --- | --- | --- | --- | --- |
| E3 | 2mpf vs. 1mpf | 2.537 | 0.0335 | * |
| E3 | 3mpf vs. 1mpf | 1.818 | 0.2072 | ns |
| E3 | 3mpf vs. 2mpf | 0.5527 | >0.9999 | ns |

**PIVE-10μM**

Kruskal-Wallis test

| **df** | **H** | **P** | **Sig.** |
| --- | --- | --- | --- |
| 2 | 30.52 | <0.0001 | ** |

Bonferroni's multiple comparisons test

| **Treatment** | **Comparison** | **Z** | **P** | **Sig.** |
| --- | --- | --- | --- | --- |
| 10μM | 2mpf vs. 1mpf | 4.604 | <0.0001 | ** |
| 10μM | 3mpf vs. 1mpf | 0.9468 | >0.9999 | ns |
| 10μM | 3mpf vs. 2mpf | 5.052 | <0.0001 | ** |

**PIVE-50μM**

Kruskal-Wallis test

| **df** | **H** | **P** | **Sig.** |
| --- | --- | --- | --- |
| 2 | 5.685 | 0.0583 | ns |

**PIVE-100μM**

Kruskal-Wallis test

| **df** | **H** | **P** | **Sig.** |
| --- | --- | --- | --- |
| 2 | 15.78 | 0.0004 | ** |

Bonferroni's multiple comparisons test

| **Treatment** | **Comparison** | **Z** | **P** | **Sig.** |
| --- | --- | --- | --- | --- |
| 100μM | 2mpf vs. 1mpf | 3.206 | 0.0040 | * |
| 100μM | 3mpf vs. 1mpf | 0.6527 | >0.9999 | ns |
| 100μM | 3mpf vs. 2mpf | 3.537 | 0.0012 | * |

**Step 3. compare different treatments within the same age**

**MIA-1mpf**

Kruskal-Wallis test

| **df** | **H** | **P** | **Sig.** |
| --- | --- | --- | --- |
| 3 | 58.95 | <0.0001 | ** |

Bonferroni's multiple comparisons test

| **Age** | **Comparison** | **Z** | **P** | **Sig.** |
| --- | --- | --- | --- | --- |
| 1mpf | PBS vs. Control | 1.569 | 0.6999 | ns |
| 1mpf | MIA-20μg/g vs. Control | 5.756 | <0.0001 | ## |
| 1mpf | MIA-50μg/g vs. Control | 1.604 | 0.6526 | ns |
| 1mpf | MIA-20μg/g vs. PBS | 7.461 | <0.0001 | $$ |
| 1mpf  1mpf | MIA-50μg/g vs. PBS  MIA-50μg/g vs. MIA-20μg/g | 3.147  3.965 | 0.0099  0.0004 | $  %% |

**MIA-2mpf**

Kruskal-Wallis test

| **df** | **H** | **P** | **Sig.** |
| --- | --- | --- | --- |
| 3 | 171.5 | <0.0001 | ** |

Bonferroni's multiple comparisons test

| **Age** | **Comparison** | **Z** | **P** | **Sig.** |
| --- | --- | --- | --- | --- |
| 2mpf | PBS vs. Control | 0.8431 | >0.9999 | ns |
| 2mpf | MIA-20μg/g vs. Control | 10.93 | <0.0001 | **##** |
| 2mpf | MIA-50μg/g vs. Control | 4.059 | 0.0003 | **##** |
| 2mpf | MIA-20μg/g vs. PBS | 11.63 | <0.0001 | $$ |
| 2mpf  2mpf | MIA-50μg/g vs. PBS  MIA-50μg/g vs. MIA-20μg/g | 4.850  6.869 | <0.0001  <0.0001 | $$  %% |

**MIA-3mpf**

Kruskal-Wallis test

| **df** | **H** | **P** | **Sig.** |
| --- | --- | --- | --- |
| 3 | 47.90 | <0.0001 | ** |

Bonferroni's multiple comparisons test

| **Age** | **Comparison** | **Z** | **P** | **Sig.** |
| --- | --- | --- | --- | --- |
| 3mpf | PBS vs. Control | 0.001579 | >0.9999 | ns |
| 3mpf | MIA-20μg/g vs. Control | 5.722 | <0.0001 | **##** |
| 3mpf | MIA-50μg/g vs. Control | 3.478 | 0.0030 | **#** |
| 3mpf | MIA-20μg/g vs. PBS | 5.776 | <0.0001 | $$ |
| 3mpf  3mpf | MIA-50μg/g vs. PBS  MIA-50μg/g vs. MIA-20μg/g | 3.508  1.968 | 0.0027  0.2947 | $  ns |

**PIVE-1mpf**

Kruskal-Wallis test

| **df** | **H** | **P** | **Sig.** |
| --- | --- | --- | --- |
| 3 | 17.56 | 0.0005 | ** |

Bonferroni's multiple comparisons test

| **Age** | **Comparison** | **Z** | **P** | **Sig.** |
| --- | --- | --- | --- | --- |
| 1mpf | 10μM vs. E3 | 2.209 | 0.1631 | ns |
| 1mpf | 50μM vs. E3 | 4.064 | 0.0003 | **††** |
| 1mpf  1mpf  1mpf  1mpf | 100 μM vs. E3  50μM vs. 10μM  100 μM vs. 10μM  100 μM vs. 50μM | 2.913  2.285  0.6597  1.815 | 0.0215  0.1340  >0.9999  0.4172 | **†**  ns  ns  ns |

**PIVE-2mpf**

Kruskal-Wallis test

| **df** | **H** | **P** | **Sig.** |
| --- | --- | --- | --- |
| 3 | 26.61 | <0.0001 | ** |

Bonferroni's multiple comparisons test

| **Age** | **Comparison** | **Z** | **P** | **Sig.** |
| --- | --- | --- | --- | --- |
| 2mpf | 10μM vs. E3 | 3.757 | 0.0010 | **†** |
| 2mpf | 50μM vs. E3 | 0.3577 | >0.9999 | ns |
| 2mpf  2mpf  2mpf  2mpf | 100μM vs. E3  50μM vs. 10μM  100 μM vs. 10μM  100 μM vs. 50μM | 3.284  3.977  0.9658  3.535 | 0.0061  0.0004  >0.9999  0.0025 | **†**  **§§**  ns  £ |

**PIVE-3mpf**

Kruskal-Wallis test

| **df** | **H** | **P** | **Sig.** |
| --- | --- | --- | --- |
| 3 | 9.701 | 0.0231 | * |

Bonferroni's multiple comparisons test

| **Age** | **Comparison** | **Z** | **P** | **Sig.** |
| --- | --- | --- | --- | --- |
| 3mpf | 10μM vs. E3 | 1.255 | >0.9999 | ns |
| 3mpf | 50μM vs. E3 | 3.088 | 0.0121 | **†** |
| 3mpf  3mpf  3mpf  3mpf | 100μM vs. E3  50μM vs. 10μM  100 μM vs. 10μM  100 μM vs. 50μM | 1.724  1.862  0.3984  1.573 | 0.5080  0.3757  >0.9999  0.6943 | ns  ns  ns  ns |

**Step 4. MIA vs. PIVE in same age**

**MIA-20μg/g vs. PIVE-10/50/100μM**

Multiple Mann-Whitney tests with Bonferroni's correction

| **Age** | **Comparison** | **U** | **P** | **Sig.** |
| --- | --- | --- | --- | --- |
| 1mpf | 10μM vs. 20μg/g | 5474 | <0.0001 | %% |
| 2mpf | 10μM vs. 20μg/g | 1048 | <0.0001 | %% |
| 3mpf | 10μM vs. 20μg/g | 4143 | <0.0001 | %% |
| 1mpf | 50μM vs. 20μg/g | 3627 | <0.0001 | %% |
| 2mpf | 50μM vs. 20μg/g | 3574 | <0.0001 | %% |
| 3mpf | 50μM vs. 20μg/g | 5224 | <0.0001 | %% |
| 1mpf | 100μM vs. 20μg/g | 8249 | 0.0013 | % |
| 2mpf | 100μM vs. 20μg/g | 4994 | <0.0001 | %% |
| 3mpf | 100μM vs. 20μg/g | 6329 | <0.0001 | %% |

**MIA-50μg/g vs. PIVE-10/50/100μM**

Multiple Mann-Whitney tests with Bonferroni's correction

| **Age** | **Comparison** | **U** | **P** | **Sig.** |
| --- | --- | --- | --- | --- |
| 1mpf | 10μM vs. 50μg/g | 9239 | 0.7602 | ns |
| 2mpf | 10μM vs. 50μg/g | 4749 | <0.0001 | @@ |
| 3mpf | 10μM vs. 50μg/g | 5423 | <0.0001 | @@ |
| 1mpf | 50μM vs. 50μg/g | 4237 | 0.0328 | @ |
| 2mpf | 50μM vs. 50μg/g | 8382 | 0.1447 | ns |
| 3mpf | 50μM vs. 50μg/g | 5809 | 0.0007 | @@ |
| 1mpf | 100μM vs. 50μg/g | 11338 | 0.8243 | ns |
| 2mpf | 100μM vs. 50μg/g | 10455 | 0.0000 | @@ |
| 3mpf | 100μM vs. 50μg/g | 7266 | 0.0000 | @@ |

- **Figure4**
- **Fig. 4C**

| **MIA-50μg/g** | | | | **PIVE-100μM** | | | **MIA+PIVE** | | |
| --- | --- | --- | --- | --- | --- | --- | --- | --- | --- |
| **Age** | | ***n*** | **M ± SEM** | **Age** | ***n*** | **M ± SEM** | **Age** | ***n*** | **M ± SEM** |
| 7dpf | 148 | | 15.27±1.80 | 7dpf | 171 | 14.13±1.57 | 7dpf | 172 | 12.52±1.50 |
| 14dpf | 124 | | 20.59±2.25 | 14dpf | 119 | 17.30±1.52 | 14dpf | 109 | 16.11±1.58 |
| 21dpf | 120 | | 18.47±2.21 | 21dpf | 83 | 18.83±2.86 | 21dpf | 134 | 11.94±1.46 |

**Step 1. Normal distribution analysis**

**Normality**

| **Shapiro-Wilk test** | | | | |
| --- | --- | --- | --- | --- |
| **Fig** | **W** | **df** | **P** | **Sig.** |
| 4-C | 0.7683 | 1180 | <0.0001 | ** |

****: nonormal distribution**

**Step 2. compare different ages within the same treatment**

**MIA-50μg/g**

Kruskal-Wallis test

| **df** | **H** | **P** | **Sig.** |
| --- | --- | --- | --- |
| 2 | 4.3910 | 0.1113 | ns |

**PIVE-100μM**

Kruskal-Wallis test

| **df** | **H** | **P** | **Sig.** |
| --- | --- | --- | --- |
| 2 | 9.696 | 0.0078 | * |

Bonferroni's multiple comparisons test

| **Treatment** | **Comparison** | **Z** | **P** | **Sig.** |
| --- | --- | --- | --- | --- |
| 100μM | 14dpf vs. 7dpf | 3.09 | 0.0060 | * |
| 100μM | 21dpf vs. 7dpf | 0.79 | >0.9999 | ns |
| 100μM | 21dpf vs. 14dpf | 1.84 | 0.1976 | ns |

**MIA+PIVE**

Kruskal-Wallis test

| **df** | **H** | **P** | **Sig.** |
| --- | --- | --- | --- |
| 2 | 11.2900 | 0.0035 | * |

Bonferroni's multiple comparisons test

| **Treatment** | **Comparison** | **Z** | **P** | **Sig.** |
| --- | --- | --- | --- | --- |
| MIA+PIVE | 14dpf vs. 7dpf | 2.9740 | 0.0088 | * |
| MIA+PIVE | 21dpf vs. 7dpf | 0.2007 | >0.9999 | ns |
| MIA+PIVE | 21dpf vs. 14dpf | 3.002 | 0.0081 | * |

**Step 3. compare different treatment within the same age**

**7dpf**

Kruskal-Wallis test

| **df** | **H** | **P** | **Sig.** |
| --- | --- | --- | --- |
| 2 | 0.0764 | 0.9625 | ns |

**14dpf**

Kruskal-Wallis test

| **df** | **H** | **P** | **Sig.** |
| --- | --- | --- | --- |
| 2 | 0.4154 | 0.8125 | ns |

**21dpf**

Kruskal-Wallis test

| **df** | **H** | **P** | **Sig.** |
| --- | --- | --- | --- |
| 2 | 2.019 | 0.3645 | ns |

- **Fig4 Db**

| **MIA-50μg/g** | | | | **PIVE-100 μM** | | | **MIA+PIVE** | | |
| --- | --- | --- | --- | --- | --- | --- | --- | --- | --- |
| **Age** | ***n*** | | **M ± SEM** | **Age** | ***n*** | **M ± SEM** | **Age** | ***n*** | **M ± SEM** |
| 1mpf | | 45 | 37.79±1.78 | 1mpf | 36 | 37.67±3.20 | 1mpf | 22 | 29.47±1.91 |
| 2mpf | | 36 | 51.86±2.64 | 2mpf | 25 | 37.32±2.60 | 2mpf | 22 | 53.49±3.64 |

**Step 1. Normal distribution analysis**

**Normality**

| **Shapiro–Wilk test** | | | | |
| --- | --- | --- | --- | --- |
| Fig | **W** | **df** | **P** | **Sig.** |
| 4-Db | 0.990 | 180 | 0.263 | ns |

**ns: normal distribution**

**Step 2. Two-way ANOVA**

| **Factor** | **df** | **F** | **P** | **Sig.** |
| --- | --- | --- | --- | --- |
| Interaction | 2 | 8.415 | 0.0005 | ** |
| Treatment | 2 | 4.4042 | 0.0204 | * |
| Age | 1 | 29.42 | <0.0001 | ** |

**compare different ages within in the same treatment**

**Šídák's multiple comparisons test**

| **Treatment** | **Comparsion** | **df** | **t** | **P** | **Sig.** |
| --- | --- | --- | --- | --- | --- |
| MIA-50μg/g | 2mpf vs. 1mpf | 71 | 4.310 | 0.0001 | ** |
| PIVE-100μM | 2mpf vs. 1mpf | 71 | 0.083 | 0.9997 | ns |
| MIA+PIVE | 2mpf vs. 1mpf | 71 | 5.016 | <0.0001 | ** |

**compare different treatments within the same age**

**Tukey's** multiple comparisons test

| **Age** | **Comparison** | **df** | **q** | **P** | **Sig.** |
| --- | --- | --- | --- | --- | --- |
| 1mpf | MIA+PIVE vs. MIA-50μg/g | 174 | 2.791 | 0.1219 | ns |
|  | MIA+PIVE vs. PIVE-100μM | 174 | 2.639 | 0.1516 | ns |
|  | PIVE-100μM vs. MIA-50μg/g | 174 | 0.050 | 0.9993 | ns |
| 2mpf | MIA+PIVE vs. MIA-50μg/g | 174 | 0.552 | 0.9193 | ns |
|  | MIA+PIVE vs. PIVE-100μM | 174 | 5.206 | 0.0009 | && |
|  | PIVE-100μM vs. MIA-50μg/g | 174 | 5.271 | 0.0008 | @@ |

- **Fig. 4Ea**

| **MIA-50μg/g** | | | | **PIVE-100 μM** | | | **MIA+PIVE** | | |
| --- | --- | --- | --- | --- | --- | --- | --- | --- | --- |
| **Age** | ***n*** | **Media [IQ3,**  **IQ1]** | **Age** | ***n*** | **Media [IQ3, IQ1]** | **Age** | ***n*** | **Media [IQ3, IQ1]** |  |
| 7dpf | | 80 | 4.17[4.69, 3.75] | 7dpf | 92 | 3.70[4.28, 3.09] | 7dpf | 76 | 3.43[3.82, 2.96] |
| 14dpf | | 60 | 4.12[4.54, 3.60] | 14dpf | 136 | 4.14[4.47, 3.67] | 14dpf | 184 | 3.79[4.14, 3.44] |
| 21dpf | | 60 | 4.13[4.62, 3.63] | 21dpf | 168 | 3.87[4.35, 3.31] | 21dpf | 184 | 3.94[4.34, 3.56] |

**Step 1. Normal distribution analysis**

**Normality**

| **Shapiro-Wilk test** | | | | |
| --- | --- | --- | --- | --- |
| **Fig** | **W** | **df** | **P** | **Sig.** |
| 4-Ea | 0.994 | 1022 | 0.000 | ****** |

****nonnormal distribution**

**Step 2. compare different ages within the same treatment**

**MIA-50μg/g**

Kruskal-Wallis test

| **df** | **H** | **P** | **Sig.** |
| --- | --- | --- | --- |
| 2 | 0.5090 | 0.7753 | ns |

**PIVE-100μM**

Kruskal-Wallis test

| **df** | **H** | **P** | **Sig.** |
| --- | --- | --- | --- |
| 2 | 22.4600 | <0.0001 | ** |

Bonferroni's multiple comparisons test

| **Treatment** | **Comparison** | **Z** | **P** | **Sig.** |
| --- | --- | --- | --- | --- |
| 100μM | 14dpf vs. 7dpf | 4.51 | <0.0001 | ** |
| 100μM | 21dpf vs. 7dpf | 1.66 | 0.2913 | **ns** |
| 100μM | 21dpf vs. 14dpf | 3.41 | 0.0019 | ***** |

**MIA+PIVE**

Kruskal-Wallis test

| **df** | **H** | **P** | **Sig.** |
| --- | --- | --- | --- |
| 2 | 46.2000 | <0.0001 | ** |

Bonferroni's multiple comparisons test

| **Treatment** | **Comparison** | **Z** | **P** | **Sig.** |
| --- | --- | --- | --- | --- |
| MIA+PIVE | 14dpf vs. 7dpf | 4.32 | <0.0001 | ** |
| MIA+PIVE | 21dpf vs. 7dpf | 6.78 | <0.0001 | **ns** |
| MIA+PIVE | 21dpf vs. 14dpf | 3.13 | 0.0052 | ***** |

**Step 3. compare different treatments within the same age**

**7dpf**

Kruskal-Wallis test

| **df** | **H** | **P** | **Sig.** |
| --- | --- | --- | --- |
| 2 | 50.5700 | <0.0001 | ** |

Bonferroni's multiple comparisons test

| **Age** | **Comparison** | **Z** | **P** | **Sig.** |
| --- | --- | --- | --- | --- |
| 7dpf | PIVE-100μM vs. MIA-50μg/g | 4.73 | <0.0001 | @@ |
| 7dpf | MIA+PIVE vs. MIA-50μg/g | 6.98 | <0.0001 | @@ |
| 7dpf | MIA+PIVE vs. PIVE-100μM | 2.55 | 0.0326 | & |

**14dpf**

Kruskal-Wallis test

| **df** | **H** | **P** | **Sig.** |
| --- | --- | --- | --- |
| 2 | 37.4800 | <0.0001 | ** |

Bonferroni's multiple comparisons test

| **Age** | **Comparison** | **Z** | **P** | **Sig.** |
| --- | --- | --- | --- | --- |
| 14dpf | PIVE-100μM vs. MIA-50μg/g | 0.06 | >0.9999 | ns |
| 14dpf | MIA+PIVE vs. MIA-50μg/g | 4.32 | <0.0001 | @@ |
| 14dpf | MIA+PIVE vs. PIVE-100μM | 5.55 | <0.0001 | && |

**21dpf**

Kruskal-Wallis test

| **df** | **H** | **P** | **Sig.** |
| --- | --- | --- | --- |
| 2 | 10.0200 | 0.0067 | * |

Bonferroni's multiple comparisons test

| **Age** | **Comparison** | **Z** | **P** | **Sig.** |
| --- | --- | --- | --- | --- |
| 21dpf | PIVE-100μM vs. MIA-50μg/g | 3.16 | 0.0047 | @ |
| 21dpf | MIA+PIVE vs. MIA-50μg/g | 2.28 | 0.0674 | ns |
| 21dpf | MIA+PIVE vs. PIVE-100μM | 1.23 | 0.6598 | ns |

- **Fig. 4Fa**

| **MIA-50μg/g** | | | **PIVE-100 μM** | | | | **MIA+PIVE** | | |  |
| --- | --- | --- | --- | --- | --- | --- | --- | --- | --- | --- |
| **Age** | ***n*** | **Media [IQ3,**  **IQ1]** | **Age** | | ***n*** | **Media [IQ3,**  **IQ1]** | **Age** | ***n*** | **Media [IQ3, IQ1]** | |
| 1mpf | 60 | 9.73[11.56, 8.50] | 1mpf | 216 | | 10.47[13.03, 7.58] | 1mpf | 100 | 12.28[15.14, 9.98] |  |
| 2mpf | 80 | 11.04[11.97, 9.54] | 2mpf | | 220 | 9.46[11.16, 7.00] | 2mpf | 100 | 11.67[13.41, 9.64] |  |

**Step 1. Normal distribution analysis**

**Normality**

| **Shapiro-Wilk test** | | | | |
| --- | --- | --- | --- | --- |
| **Fig** | **W** | **df** | **P** | **Sig.** |
| 4Fa | 0.988 | 1022 | 0.000 | ** |

****nonnormal distribution**

**Step 2. compare different ages within the same treatment**

**2mpf vs. 1mpf**

Multiple Mann-Whitney tests with Bonferroni's correction

| **Treatment** | **Comparison** | **Z** | **P** | **Sig.** |
| --- | --- | --- | --- | --- |
| MIA-50μg/g | 2mpf vs. 1mpf | 4407.00 | 0.0225 | * |
| PIVE-100μM | 3mpf vs. 1mpf | 27921.00 | 0.0114 | * |
| MIA+PIVE | 3mpf vs. 2mpf | 9380.00 | 0.0385 | * |

**Step 3. compare different treatments within the same age**

**1mpf**

Kruskal-Wallis test

| **df** | **H** | **P** | **Sig.** |
| --- | --- | --- | --- |
| 2 | 34.3800 | <0.0001 | ** |

Bonferroni's multiple comparisons test

| **Age** | **Comparison** | **Z** | **P** | **Sig.** |
| --- | --- | --- | --- | --- |
| 1mpf | PIVE-100μM vs. MIA-50μg/g | 0.48 | >0.9999 | ns |
| 1mpf | MIA+PIVE vs. MIA-50μg/g | 4.61 | <0.0001 | @@ |
| 1mpf | MIA+PIVE vs. PIVE-100μM | 5.40 | <0.0001 | && |

**2mpf**

Kruskal-Wallis test

| **df** | **H** | **P** | **Sig.** |
| --- | --- | --- | --- |
| 2 | 53.0200 | <0.0001 | ** |

Bonferroni's multiple comparisons test

| **Age** | **Comparison** | **Z** | **P** | **Sig.** |
| --- | --- | --- | --- | --- |
| 2mpf | PIVE-100μM vs. MIA-50μg/g | 4.57 | <0.0001 | @@ |
| 2mpf | MIA+PIVE vs. MIA-50μg/g | 1.66 | 0.2929 | ns |
| 2mpf | MIA+PIVE vs. PIVE-100μM | 6.89 | <0.0001 | && |

- **Figure 5**

| - **Fig. 5C** | | | | | | |  |
| --- | --- | --- | --- | --- | --- | --- | --- |
| **MIA** | | | | **PIVE** | | | |
|  | **Age** | ***n*** | **M ± SEM** |  | **Age** | ***n*** | **M ± SEM** |
| Control | 1mpf | 35 | 10.11±0.72 | E3 | 1mpf | 52 | 14.16±0.97 |
|  | 2mpf | 28 | 9.109±0.86 |  | 2mpf | 38 | 12.80±1.37 |
|  | 3mpf | 21 | 8.566±1.14 |  | 3mpf | 25 | 11.28±1.76 |
| PBS | 1mpf | 42 | 11.05±0.88 | 10μM | 1mpf | 35 | 14.77±1.34 |
|  | 2mpf | 41 | 7.259±0.60 |  | 2mpf | 25 | 12.19±1.51 |
|  | 3mpf | 27 | 8.145±0.98 |  | 3mpf | 28 | 9.630±1.32 |
| 20μg/g | 1mpf | 56 | 9.204±0.72 | 50μM | 1mpf | 48 | 13.26±1.08 |
|  | 2mpf | 31 | 9.280±0.99 |  | 2mpf | 38 | 9.123±0.83 |
|  | 3mpf | 37 | 12.27±1.51 |  | 3mpf | 29 | 10.60±1.69 |
| 50μg/g | 1mpf | 61 | 15.17±0.74 | 100μM | 1mpf | 61 | 9.274±0.74 |
|  | 2mpf | 35 | 7.844±0.80 |  | 2mpf | 22 | 10.29±2.00 |
|  | 3mpf | 33 | 8.477±0.85 |  | 3mpf | 28 | 10.36±1.47 |

**Step 1. Normal distribution analysis**

**Normality**

| **Shapiro–Wilk test** | | | | |
| --- | --- | --- | --- | --- |
| **Fig** | **W** | **df** | **P** | **Sig.** |
| 5-C | 0.921 | 813 | 0.000 | ** |

****nonnormal distribution**

**Step 2. compare different ages within the same treatment**

**MIA-Control**

Kruskal-Wallis test

| **df** | **H** | **P** | **Sig.** |
| --- | --- | --- | --- |
| 2 | 0.7705 | 0.6803 | ns |

**MIA-PBS**

Kruskal-Wallis test

| **df** | **H** | **P** | **Sig.** |
| --- | --- | --- | --- |
| 2 | 8.752 | 0.0126 | * |

Bonferroni's multiple comparisons test

| **Treatment** | **Comparison** | **Z** | **P** | **Sig.** |
| --- | --- | --- | --- | --- |
| PBS | 2mpf vs. 1mpf | 2.36 | 0.055 | ns |
| PBS | 3mpf vs. 1mpf | 2.69 | 0.0214 | * |
| PBS | 3mpf vs. 2mpf | 0.57 | >0.9999 | ns |

**MIA-20μg/g**

Kruskal-Wallis test

| **df** | **H** | **P** | **Sig.** |
| --- | --- | --- | --- |
| 2 | 1.3110 | 0.5192 | ns |

**MIA-50μg/g**

Kruskal-Wallis test

| **df** | **H** | **P** | **Sig.** |
| --- | --- | --- | --- |
| 2 | 43.7900 | <0.0001 | ** |

Bonferroni's multiple comparisons test

| **Treatment** | **Comparison** | **Z** | **P** | **Sig.** |
| --- | --- | --- | --- | --- |
| 50μg/g | 2mpf vs. 1mpf | 5.81 | <0.0001 | ** |
| 50μg/g | 3mpf vs. 1mpf | 5.03 | <0.0001 | ****** |
| 50μg/g | 3mpf vs. 2mpf | 0.60 | >0.9999 | **ns** |

**PIVE-E3**

Kruskal-Wallis test

| **df** | **H** | **P** | **Sig.** |
| --- | --- | --- | --- |
| 2 | 0.1469 | 0.9292 | ns |

**PIVE-10μM**

Kruskal-Wallis test

| **df** | **H** | **P** | **Sig.** |
| --- | --- | --- | --- |
| 2 | 6.8000 | 0.0334 | * |

**Bonferroni's multiple comparisons test**

| **Treatment** | **Comparison** | **Z** | **P** | **Sig.** |
| --- | --- | --- | --- | --- |
| 10μM | 2mpf vs. 1mpf | 1.06 | 0.8686 | ns |
| 10μM | 3mpf vs. 1mpf | 2.61 | 0.0274 | ***** |
| 10μM | 3mpf vs. 2mpf | 1.39 | 0.4899 | ns |

**PIVE-50μM**

Kruskal-Wallis test

| **df** | **H** | **P** | **Sig.** |
| --- | --- | --- | --- |
| 2 | 8.1020 | 0.0174 | * |

Bonferroni's multiple comparisons test

| **Treatment** | **Comparison** | **Z** | **P** | **Sig.** |
| --- | --- | --- | --- | --- |
| 50μM | 2mpf vs. 1mpf | 2.56 | 0.0316 | ns |
| 50μM | 3mpf vs. 1mpf | 2.18 | 0.0869 | ns |
| 50μM | 3mpf vs. 2mpf | 0.17 | >0.9999 | ns |

**PIVE-100μM**

Kruskal-Wallis test

| **df** | **H** | **P** | **Sig.** |
| --- | --- | --- | --- |
| 2 | 0.0766 | 0.9624 | ns |

**Step 3. compare different treatments within the same age**

**MIA-1mpf**

Kruskal-Wallis test

| **df** | **H** | **P** | **Sig.** |
| --- | --- | --- | --- |
| 3 | 35.50 | <0.0001 | ** |

Bonferroni's multiple comparisons test

| **Age** | **Comparison** | **Z** | **P** | **Sig.** |
| --- | --- | --- | --- | --- |
| 1mpf | PBS vs. Control | 0.71 | >0.9999 | ns |
| 1mpf | 20μg/g vs. Control | 0.92 | >0.9999 | ns |
| 1mpf | 50μg/g vs. Control | 4.41 | <0.0001 | **##** |
| 1mpf | 20μg/g vs. PBS | 1.61 | 0.6425 | ns |
| 1mpf | 50μg/g vs. PBS | 3.48 | 0.0030 | **$** |
| 1mpf | 50μg/g vs. 20μg/g | 5.55 | <0.0001 | **%** |

**MIA-2mpf**

Kruskal-Wallis test

| **df** | **H** | **P** | **Sig.** |
| --- | --- | --- | --- |
| 3 | 1.725 | 0.6313 | ns |

**MIA-3mpf**

Kruskal-Wallis test

| **df** | **H** | **P** | **Sig.** |
| --- | --- | --- | --- |
| 3 | 1.959 | 0.5809 | ns |

**PIVE-1mpf**

Kruskal-Wallis test

| **df** | **H** | **P** | **Sig.** |
| --- | --- | --- | --- |
| 3 | 7.782 | 0.0507 | ns |

**PIVE-2mpf**

Kruskal-Wallis test

| **df** | **H** | **P** | **Sig.** |
| --- | --- | --- | --- |
| 3 | 4.526 | 0.2100 | ns |

**PIVE-3mpf**

Kruskal-Wallis test

| **df** | **H** | **P** | **Sig.** |
| --- | --- | --- | --- |
| 3 | 2.893 | 0.4084 | ns |

**Step 4. MIA vs. PIVE in same age**

**MIA-20 μg/g vs. PIVE-10/ 50/100 μM**

Multiple Mann-Whitney tests with Bonferroni's correction

| **Age** | **Comparison** | **U** | **P** | **Sig.** |
| --- | --- | --- | --- | --- |
| 1mpf | 10μM vs. 20μg/g | 571.00 | 0.0021 | % |
| 2mpf | 10μM vs. 20μg/g | 314.00 | 0.6928 | ns |
| 3mpf | 10μM vs. 20μg/g | 435.50 | 0.8339 | ns |
| 1mpf | 50μM vs. 20μg/g | 897.00 | 0.0099 | % |
| 2mpf | 50μM vs. 20μg/g | 577.50 | >0.9999 | ns |
| 3mpf | 50μM vs. 20μg/g | 474.50 | >0.9999 | ns |
| 1mpf | 100μM vs. 20μg/g | 1693.00 | >0.9999 | ns |
| 2mpf | 100μM vs. 20μg/g | 334.00 | >0.9999 | ns |
| 3mpf | 100μM vs. 20μg/g | 469.00 | >0.9999 | ns |

**MIA-50μg/g vs. PIVE-10/50/100μM**

Multiple Mann-Whitney tests with Bonferroni's correction

| **Age** | **Comparison** | **U** | **P** | **Sig.** |
| --- | --- | --- | --- | --- |
| 1mpf | 10μM vs. 50μg/g | 972.00 | >0.9999 | ns |
| 2mpf | 10μM vs. 50μg/g | 293.50 | 0.0911 | ns |
| 3mpf | 10μM vs. 50μg/g | 440.00 | >0.9999 | ns |
| 1mpf | 50μM vs. 50μg/g | 1142.00 | 0.1483 | ns |
| 2mpf | 50μM vs. 50μg/g | 552.50 | 0.6497 | ns |
| 3mpf | 50μM vs. 50μg/g | 437.00 | >0.9999 | ns |
| 1mpf | 100μM vs. 50μg/g | 835.00 | <0.000001 | @@ |
| 2mpf | 100μM vs. 50μg/g | 343.50 | >0.9999 | ns |
| 3mpf | 100μM vs. 50μg/g | 417.50 | >0.9999 | ns |

| - **Fig. 5E** | | | | | | |  |
| --- | --- | --- | --- | --- | --- | --- | --- |
| **MIA** | | | | **PIVE** | | | |
|  | **Age** | ***n*** | **M ± SEM** |  | **Age** | ***n*** | **M ± SEM** |
| Control | 1mpf | 35 | 26.10±3.85 | E3 | 1mpf | 47 | 37.06±3.67 |
|  | 2mpf | 37 | 36.34±4.57 |  | 2mpf | 33 | 27.60±3.74 |
|  | 3mpf | 33 | 38.56±4.35 |  | 3mpf | 24 | 27.22±3.74 |
| PBS | 1mpf | 39 | 32.83±2.75 | 10μM | 1mpf | 46 | 37.35±3.60 |
|  | 2mpf | 21 | 36.70±4.04 |  | 2mpf | 40 | 29.12±3.83 |
|  | 3mpf | 28 | 35.01±4.00 |  | 3mpf | 28 | 32.74±5.44 |
| 20μg/g | 1mpf | 37 | 51.32±3.56 | 50μM | 1mpf | 45 | 47.63±3.59 |
|  | 2mpf | 27 | 40.22±5.21 |  | 2mpf | 46 | 39.64±4.10 |
|  | 3mpf | 22 | 43.47±5.75 |  | 3mpf | 29 | 18.89±3.62 |
| 50μg/g | 1mpf | 32 | 54.69±4.42 | 100μM | 1mpf | 45 | 53.87±4.05 |
|  | 2mpf | 40 | 35.56±3.37 |  | 2mpf | 26 | 29.67±3.75 |
|  | 3mpf | 24 | 46.17±6.45 |  | 3mpf | 20 | 26.40±6.00 |

**Step 1. Normal distribution analysis**

**Normality**

| **Shapiro-Wilk test** | | | | |
| --- | --- | --- | --- | --- |
| **Fig** | **W** | **df** | **P** | **Sig.** |
| 5-E | 0.947 | 813 | 0.000 | ** |

****nonnormal distribution**

**Step 2. compare different ages within the same treatment**

**MIA-Control**

Kruskal-Wallis test

| **df** | **H** | **P** | **Sig.** |
| --- | --- | --- | --- |
| 2 | 2.29 | 0.3181 | ns |

**MIA-PBS**

Kruskal-Wallis test

| **df** | **H** | **P** | **Sig.** |
| --- | --- | --- | --- |
| 2 | 0.498 | 0.7795 | ns |

**MIA-20μg/g**

Kruskal-Wallis test

| **df** | **H** | **P** | **Sig.** |
| --- | --- | --- | --- |
| 2 | 3.63 | 0.1627 | ns |

**MIA-50μg/g**

Kruskal-Wallis test

| **df** | **H** | **P** | **Sig.** |
| --- | --- | --- | --- |
| 2 | 9.21 | 0.0100 | ns |

Bonferroni's multiple comparisons test

| **Treatment** | **Comparison** | **Z** | **P** | **Sig.** |
| --- | --- | --- | --- | --- |
| 50μg/g | 2mpf vs. 1mpf | 3.03 | 0.0073 | * |
| 50μg/g | 3mpf vs. 1mpf | 1.33 | 0.5498 | ns |
| 50μg/g | 3mpf vs. 2mpf | 1.39 | 0.4922 | ns |

**PIVE-E3**

Kruskal-Wallis test

| **df** | **H** | **P** | **Sig.** |
| --- | --- | --- | --- |
| 2 | 3.459 | 0.1774 | ns |

**PIVE-10μM**

Kruskal-Wallis test

| **df** | **H** | **P** | **Sig.** |
| --- | --- | --- | --- |
| 2 | 2.60400 | 0.2720 | ns |

**PIVE-50μM**

Kruskal-Wallis test

| **df** | **H** | **P** | **Sig.** |
| --- | --- | --- | --- |
| 2 | 22.5500 | <0.0001 | ** |

Bonferroni's multiple comparisons test

| **Treatment** | **Comparison** | **Z** | **P** | **Sig.** |
| --- | --- | --- | --- | --- |
| 50μM | 2mpf vs. 1mpf | 1.71 | 0.2601 | ns |
| 50μM | 3mpf vs. 1mpf | 4.73 | <0.0001 | ** |
| 50μM | 3mpf vs. 2mpf | 3.23 | 0.0037 | * |

**PIVE-100μM**

Kruskal-Wallis test

| **df** | **H** | **P** | **Sig.** |
| --- | --- | --- | --- |
| 2 | 20.1800 | <0.0001 | ** |

Bonferroni's multiple comparisons test

| **Treatment** | **Comparison** | **Z** | **P** | **Sig.** |
| --- | --- | --- | --- | --- |
| 100μM | 2mpf vs. 1mpf | 3.38 | 0.0022 | * |
| 100μM | 3mpf vs. 1mpf | 3.92 | 0.0003 | ** |
| 100μM | 3mpf vs. 2mpf | 0.75 | >0.9999 | ns |

**Step 3. compare different treatments within the same age**

**MIA-1mpf**

Kruskal-Wallis test

| **df** | **H** | **P** | **Sig.** |
| --- | --- | --- | --- |
| 3 | 31.88 | <0.0001 | ** |

Bonferroni's multiple comparisons test

| **Age** | **Comparison** | **Z** | **P** | **Sig.** |
| --- | --- | --- | --- | --- |
| 1mpf | PBS vs. Control | 0.81 | >0.9999 | ns |
| 1mpf | 20μg/g vs. Control | 4.17 | 0.0002 | **##** |
| 1mpf | 50μg/g vs. Control | 4.45 | <0.0001 | **##** |
| 1mpf | 20μg/g vs. PBS | 3.46 | 0.0032 | $ |
| 1mpf | 50μg/g vs. PBS | 3.77 | 0.001 | $ |
| 1mpf | 20μg/g vs. 50μg/g | 0.43 | >0.9999 | ns |

**MIA-2mpf**

Kruskal-Wallis test

| **df** | **H** | **P** | **Sig.** |
| --- | --- | --- | --- |
| 3 | 0.7159 | 0.8695 | ns |

**MIA-3mpf**

Kruskal-Wallis test

| **df** | **H** | **P** | **Sig.** |
| --- | --- | --- | --- |
| 3 | 2.325 | 0.5077 | ns |

**PIVE-1mpf**

Kruskal-Wallis test

| **df** | **H** | **P** | **Sig.** |
| --- | --- | --- | --- |
| 3 | 15.06 | 0.0018 | * |

Bonferroni's multiple comparisons test

| **Age** | **Comparison** | **Z** | **P** | **Sig.** |
| --- | --- | --- | --- | --- |
| 1mpf | 10μM vs. E3 | 0.09 | >0.9999 | ns |
| 1mpf | 50μΜ vs. E3 | 2.19 | 0.1725 | ns |
| 1mpf | 100μM vs. E3 | 3.21 | 0.008 | **†** |
| 1mpf | 50μM vs. 10μM | 2.09 | 0.2192 | ns |
| 1mpf | 100μM vs. 10μM | 3.11 | 0.0113 | **§** |
| 1mpf | 100μM vs. 50μM | 1.01 | >0.9999 | ns |

**PIVE-2mpf**

Kruskal-Wallis test

| **df** | **H** | **P** | **Sig.** |
| --- | --- | --- | --- |
| 3 | 5.039 | 0.1690 | ns |

**PIVE-3mpf**

Kruskal-Wallis test

| **df** | **H** | **P** | **Sig.** |
| --- | --- | --- | --- |
| 3 | 4.334 | 0.2276 | ns |

**Step 4. MIA vs. PIVE in same age**

**MIA-20 μg/g vs. PIVE-10/ 50/100 μM**

Multiple Mann-Whitney tests with Bonferroni's correction

| **Age** | **Comparison** | **U** | **P** | **Sig.** |
| --- | --- | --- | --- | --- |
| 1mpf | 10μM vs. 20μg/g | 555.00 | 0.0190 | % |
| 2mpf | 10μM vs. 20μg/g | 402.00 | 0.2362 | ns |
| 3mpf | 10μM vs. 20μg/g | 230.00 | 0.3886 | ns |
| 1mpf | 50μM vs. 20μg/g | 746.00 | >0.9999 | ns |
| 2mpf | 50μM vs. 20μg/g | 607.00 | >0.9999 | ns |
| 3mpf | 50μM vs. 20μg/g | 135.00 | 0.0009 | %% |
| 1mpf | 100μM vs. 20μg/g | 796.00 | >0.9999 | ns |
| 2mpf | 100μM vs. 20μg/g | 280.00 | 0.6348 | ns |
| 3mpf | 100μM vs. 20μg/g | 129.00 | 0.0646 | ns |

**MIA-50μg/g vs. PIVE-10/50/100μM**

Multiple Mann-Whitney tests with Bonferroni's correction

| **Age** | **Comparison** | **U** | **P** | **Sig.** |
| --- | --- | --- | --- | --- |
| 1mpf | 10μM vs. 50μg/g | 428.00 | 0.0046 | @ |
| 2mpf | 10μM vs. 50μg/g | 634.00 | 0.3348 | ns |
| 3mpf | 10μM vs. 50μg/g | 240.00 | 0.2364 | ns |
| 1mpf | 50μM vs. 50μg/g | 603.00 | 0.6900 | ns |
| 2mpf | 50μM vs. 50μg/g | 873.00 | >0.9999 | ns |
| 3mpf | 50μM vs. 50μg/g | 162.00 | 0.0020 | @ |
| 1mpf | 100μM vs. 50μg/g | 717.00 | >0.9999 | ns |
| 2mpf | 100μM vs. 50μg/g | 427.00 | 0.6796 | ns |
| 3mpf | 100μM vs. 50μg/g | 143.00 | 0.0655 | ns |

| - **Fig. 5G** | | | | | | |  |
| --- | --- | --- | --- | --- | --- | --- | --- |
| **MIA** | | | | **PIVE** | | | |
|  | **Age** | ***n*** | **M ± SEM** |  | **Age** | ***n*** | **M ± SEM** |
| Control | 1mpf | 44 | 17.58±2.22 | E3 | 1mpf | 47 | 22.28±2.80 |
|  | 2mpf | 32 | 19.33±2.65 |  | 2mpf | 41 | 21.60±2.21 |
|  | 3mpf | 30 | 21.70±3.21 |  | 3mpf | 23 | 22.32±3.15 |
| PBS | 1mpf | 38 | 16.06±2.02 | 10μM | 1mpf | 47 | 16.64±1.82 |
|  | 2mpf | 44 | 24.97±2.79 |  | 2mpf | 24 | 17.17±2.54 |
|  | 3mpf | 30 | 27.64±33.59 |  | 3mpf | 26 | 26.82±3.43 |
| 20μg/g | 1mpf | 51 | 14.56±1.52 | 50μM | 1mpf | 44 | 19.76±2.51 |
|  | 2mpf | 39 | 25.24±3.42 |  | 2mpf | 36 | 26.61±3.23 |
|  | 3mpf | 25 | 22.19±2.35 |  | 3mpf | 28 | 24.09±4.23 |
| 50μg/g | 1mpf | 47 | 9.992±1.11 | 100μM | 1mpf | 23 | 9.799±1.21 |
|  | 2mpf | 50 | 24.82±2.88 |  | 2mpf | 28 | 24.63±2.97 |
|  | 3mpf | 31 | 22.59±2.78 |  | 3mpf | 30 | 30.77±4.35 |

**Step 1. Normal distribution analysis**

**Normality**

| **Shapiro-Wilk test** | | | | |
| --- | --- | --- | --- | --- |
| **Fig** | **W** | **df** | **P** | **Sig.** |
| 5-G | 0.872 | 813 | 0.000 | ****** |

****nonnormal distribution**

**Step 2. compare different ages within the same treatment**

**MIA-Control**

Kruskal-Wallis test

| **df** | **H** | **P** | **Sig.** |
| --- | --- | --- | --- |
| 2 | 0.7705 | 0.6803 | ns |

**MIA-PBS**

Kruskal-Wallis test

| **df** | **H** | **P** | **Sig.** |
| --- | --- | --- | --- |
| 2 | 8.7520 | 0.0126 | ***** |

Bonferroni's multiple comparisons test

| **Treatment** | **Comparison** | **Z** | **P** | **Sig.** |
| --- | --- | --- | --- | --- |
| PBS | 2mpf vs. 1mpf | 2.36 | 0.055 | ns |
| PBS | 3mpf vs. 1mpf | 2.69 | 0.0214 | * |
| PBS | 3mpf vs. 2mpf | 0.57 | >0.9999 | ns |

**MIA-20μg/g**

Kruskal-Wallis test

| **df** | **H** | **P** | **Sig.** |
| --- | --- | --- | --- |
| 2 | 10.3200 | 0.0058 | ***** |

Bonferroni's multiple comparisons test

| **Treatment** | **Comparison** | **Z** | **P** | **Sig.** |
| --- | --- | --- | --- | --- |
| 20μg/g | 2mpf vs. 1mpf | 2.53 | 0.0343 | * |
| 20μg/g | 3mpf vs. 1mpf | 2.76 | 0.0171 | * |
| 20μg/g | 3mpf vs. 2mpf | 0.56 | >0.9999 | ns |

**MIA-50μg/g**

Kruskal-Wallis test

| **df** | **H** | **P** | **Sig.** |
| --- | --- | --- | --- |
| 2 | 24.7600 | <0.0001 | ****** |

Bonferroni's multiple comparisons test

| **Treatment** | **Comparison** | **Z** | **P** | **Sig.** |
| --- | --- | --- | --- | --- |
| 50μg/g | 2mpf vs. 1mpf | 4.45 | <0.0001 | ** |
| 50μg/g | 3mpf vs. 1mpf | 4.01 | 0.0002 | ** |
| 50μg/g | 3mpf vs. 2mpf | 0.10 | >0.9999 | ns |

**PIVE-E3**

Kruskal-Wallis test

| **df** | **H** | **P** | **Sig.** |
| --- | --- | --- | --- |
| 2 | 0.3395 | 0.8439 | ns |

**PIVE-10μM**

Kruskal-Wallis test

| **df** | **H** | **P** | **Sig.** |
| --- | --- | --- | --- |
| 2 | 7.7300 | 0.0209 | * |

Bonferroni's multiple comparisons test

| **Treatment** | **Comparison** | **Z** | **P** | **Sig.** |
| --- | --- | --- | --- | --- |
| 10μM | 2mpf vs. 1mpf | 0.28 | >0.9999 | ns |
| 10μM | 3mpf vs. 1mpf | 2.70 | 0.0209 | * |
| 10μM | 3mpf vs. 2mpf | 2.07 | 0.1156 | ns |

**PIVE-50μM**

Kruskal-Wallis test

| **df** | **H** | **P** | **Sig.** |
| --- | --- | --- | --- |
| 2 | 3.157 | 0.2062 | ns |

**PIVE-100μM**

Kruskal-Wallis test

| **df** | **H** | **P** | **Sig.** |
| --- | --- | --- | --- |
| 2 | 18.9100 | <0.0001 | ** |

Bonferroni's multiple comparisons test

| **Treatment** | **Comparison** | **Z** | **P** | **Sig.** |
| --- | --- | --- | --- | --- |
| 100μM | 2mpf vs. 1mpf | 3.48 | 0.0015 | * |
| 100μM | 3mpf vs. 1mpf | 4.07 | 0.0001 | ** |
| 100μM | 3mpf vs. 2mpf | 0.55 | >0.9999 | ns |

**Step 3. compare different treatments within the same age**

**MIA-1mpf**

Kruskal-Wallis test

| **df** | **H** | **P** | **Sig.** |
| --- | --- | --- | --- |
| 3 | 12.9600 | 0.0047 | * |

Bonferroni's multiple comparisons test

| **Age** | **Comparison** | **Z** | **P** | **Sig.** |
| --- | --- | --- | --- | --- |
| 1mpf | PBS vs. Control | 0.11 | >0.9999 | ns |
| 1mpf | 20μg/g vs. Control | 0.97 | >0.9999 | ns |
| 1mpf | 50μg/g vs. Control | 3.17 | 0.0092 | **#** |
| 1mpf | 20μg/g vs. PBS | 0.83 | >0.9999 | ns |
| 1mpf | 50μg/g vs. PBS | 2.98 | 0.0173 | $ |
| 1mpf | 50μg/g vs. 20μg/g | 2.33 | 0.1191 | ns |

**MIA-2mpf**

Kruskal-Wallis test

| **df** | **H** | **P** | **Sig.** |
| --- | --- | --- | --- |
| 3 | 1.7250 | 0.6313 | ns |

**MIA-3mpf**

Kruskal-Wallis test

| **df** | **H** | **P** | **Sig.** |
| --- | --- | --- | --- |
| 3 | 1.9590 | 0.5809 | ns |

**PIVE-1mpf**

Kruskal-Wallis test

| **df** | **H** | **P** | **Sig.** |
| --- | --- | --- | --- |
| 3 | 7.782 | 0.0507 | ns |

**PIVE-2mpf**

Kruskal-Wallis test

| **df** | **H** | **P** | **Sig.** |
| --- | --- | --- | --- |
| 3 | 4.526 | 0.2100 | ns |

**PIVE-3mpf**

Kruskal-Wallis test

| **df** | **H** | **P** | **Sig.** |
| --- | --- | --- | --- |
| 3 | 2.893 | 0.4084 | ns |

**Step 4. MIA vs. PIVE in same age**

**MIA-20 μg/g vs. PIVE-10/ 50/100 μM**

Multiple Mann-Whitney tests with Bonferroni's correction

| **Age** | **Comparison** | **U** | **P** | **Sig.** |
| --- | --- | --- | --- | --- |
| 1mpf | 10μM vs. 20μg/g | 1186.00 | >0.9999 | ns |
| 2mpf | 10μM vs. 20μg/g | 387.00 | 0.7701 | ns |
| 3mpf | 10μM vs. 20μg/g | 266.50 | >0.9999 | ns |
| 1mpf | 50μM vs. 20μg/g | 1056.00 | 0.7643 | ns |
| 2mpf | 50μM vs. 20μg/g | 652.50 | >0.9999 | ns |
| 3mpf | 50μM vs. 20μg/g | 297.00 | >0.9999 | ns |
| 1mpf | 100μM vs. 20μg/g | 553.00 | 0.7321 | ns |
| 2mpf | 100μM vs. 20μg/g | 515.00 | >0.9999 | ns |
| 3mpf | 100μM vs. 20μg/g | 306.00 | >0.9999 | ns |

**MIA-50μg/g vs. PIVE-10/50/100μM**

Multiple Mann-Whitney tests with Bonferroni's correction

| **Age** | **Comparison** | **U** | **P** | **Sig.** |
| --- | --- | --- | --- | --- |
| 1mpf | 10μM vs. 50μg/g | 778.00 | 0.0176 | @ |
| 2mpf | 10μM vs. 50μg/g | 479.00 | 0.4930 | ns |
| 3mpf | 10μM vs. 50μg/g | 350.00 | >0.9999 | ns |
| 1mpf | 50μM vs. 50μg/g | 682.00 | 0.0059 | @ |
| 2mpf | 50μM vs. 50μg/g | 838.00 | >0.9999 | ns |
| 3mpf | 50μM vs. 50μg/g | 403.00 | >0.9999 | ns |
| 1mpf | 100μM vs. 0μg/g | 525.00 | >0.9999 | ns |
| 2mpf | 100μM vs. 50μg/g | 664.50 | >0.9999 | ns |
| 3mpf | 100μM vs. 50μg/g | 383.00 | 0.7248 | ns |

- **Figure 6**
- **Fig. 6J**

| **MIA** | | | | **PIVE** | | | |
| --- | --- | --- | --- | --- | --- | --- | --- |
|  | **Gene** | ***n*** | **M ± SEM** |  | **Gene** | ***n*** | **M ± SEM** |
| PBS | *nme2b.2* | 5 | 1.00±0.045 | E3 | nme2b.2 | 5 | 1.00±0.19 |
|  | *pvalb1* | 5 | 1.00±0.11 |  | pvalb1 | 5 | 1.00±0.22 |
|  | *pvalb2* | 5 | 1.00±0.17 |  | pvalb2 | 5 | 1.00±0.22 |
|  | *fkbp5* | 5 | 1.00±0.14 |  | fkbp5 | 5 | 1.00±0.18 |
|  | *hbae1.2* | 5 | 1.00±0.21 |  | hbae1.2 | 5 | 1.00±0.09 |
|  | *hbbe1.1* | 5 | 1.00±0.22 |  | hbbe1.1 | 5 | 1.00±0.16 |
|  | *fabp2* | 5 | 1.00±0.10 |  | fabp2 | 5 | 1.00±0.11 |
| 50 μg/g | *nme2b.2* | 5 | 1.56±0.12 | 100μM | nme2b.2 | 5 | 1.84±0.07 |
|  | *pvalb1* | 5 | 17.24±0.47 |  | pvalb1 | 5 | 3.85±0.31 |
|  | *pvalb2* | 5 | 16.63±2.709 |  | pvalb2 | 5 | 3.85±0.45 |
|  | *fkbp5* | 5 | 0.17±0.03 |  | fkbp5 | 5 | 0.43±0.06 |
|  | *hbae1.2* | 5 | 0.63±0.04 |  | hbae1.2 | 5 | 3.43±0.23 |
|  | *hbbe1.1* | 5 | 0.41±0.08 |  | hbbe1.1 | 5 | 2.44±0.23 |
|  | *fabp2* | 5 | 13.78±1.46 |  | fabp2 | 5 | 0.19±0.02 |

**Step 1. Normal distribution analysis**

**Normality**

| **Shapiro-Wilk test** | | | | | | | | | |
| --- | --- | --- | --- | --- | --- | --- | --- | --- | --- |
| **MIA** | | | | | **PIVE** | | | | |
| **Gene** | **W** | **df** | **P** | **Sig.** | **Gene** | **W** | **df** | **P** | **Sig.** |
| *nme2b.2* | 0.920 | 10 | 0.3541 | ns | *nme2b.2* | 0.934 | 10 | 0.4868 | ns |
| *pvalb1* | 0.717 | 10 | 0.0014 | ** | *pvalb1* | 0.871 | 10 | 0.1026 | ns |
| *pvalb2* | 0.763 | 10 | 0.0051 | ** | *pvalb2* | 0.888 | 10 | 0.1590 | ns |
| *fkbp5* | 0.888 | 10 | 0.1621 | ns | *fkbp5* | 0.887 | 10 | 0.1568 | ns |
| *hbae1.2* | 0.830 | 10 | 0.0335 | * | *hbae1.2* | 0.807 | 10 | 0.0175 | * |
| *hbbe1.1* | 0.837 | 10 | 0.0871 | ns | *hbbe1.1* | 0.893 | 10 | 0.1852 | ns |
| *fabp2* | 0.793 | 10 | 0.0120 | * | *fabp2* | 0.822 | 10 | 0.0269 | * |

***/**nonnormal distribution**

**ns: normal distribution**

**Step 2. compare within PBS and 50μg/g**

| MIA | | | | | |
| --- | --- | --- | --- | --- | --- |
| **Gene** | **Comparison** | **Statistical analysis** | **t or U** | **P** | **Sig.** |
| *nme2b.2* | 50μg/g vs. PBS | Unpaired t test | 4.33 | 0.0025 | * |
| *pvalb1* | 50μg/g vs. PBS | Mann-Whitney test | 25.00 | 0.0079 | * |
| *pvalb2* | 50μg/g vs. PBS | Mann-Whitney test | 25.00 | 0.0079 | * |
| *fkbp5* | 50μg/g vs. PBS | Unpaired t test | 1.78 | 0.0004 | ** |
| *hbae1.2* | 50μg/g vs. PBS | Mann-Whitney test | 7.00 | 0.3095 | ns |
| *hbbe1.1* | 50μg/g vs. PBS | Unpaired t test | 2.50 | 0.0368 | * |
| *fabp2* | 50μg/g vs. PBS | Mann-Whitney test | 25.00 | 0.0079 | * |

**Unpaired t test: Independent sample T test (two-tail)**

**Step 3. compare within E3 and 100μM**

| PIVE | | | | | |
| --- | --- | --- | --- | --- | --- |
| **Gene** | **Comparison** | **Statistical analysis** | **t or U** | **P** | **Sig.** |
| *nme2b.2* | 100μM vs. E3 | Unpaired t test | 4.18 | 0.0031 | * |
| *pvalb1* | 100μM vs. E3 | Unpaired t test | 7.63 | <0.0001 | ** |
| *pvalb2* | 100μM vs. E3 | Unpaired t test | 5.62 | 0.0005 | ** |
| *fkbp5* | 100μM vs. E3 | Unpaired t test | 3.04 | 0.016 | * |
| *hbae1.2* | 100μM vs. E3 | Mann-Whitney test | 25.00 | 0.0079 | * |
| *hbbe1.1* | 100μM vs. E3 | Unpaired t test | 5.13 | 0.0009 | ** |
| *fabp2* | 100μM vs. E3 | Mann-Whitney test | 25.00 | 0.0079 | * |

**Unpaired t test: Independent sample T test (two-tail)**

- **Figure 7**
- **Fig. 7Ha-b**

| **Group** | **Age** | | ***n*** | **M ± SEM** |
| --- | --- | --- | --- | --- |
| MIA-50μg/g-*fabp2^-/-^* | | 21dpf | 4 | 0.36±0.17 |
| MIA-50μg/g-*fabp2^+/+^* | | 21dpf | 4 | 1.00±0.16 |
| PIVE-100μM-*fabp2^-/-^* | | 21dpf | 4 | 0.06±0.01 |
| PIVE-100μM-*fabp2^+/+^* | | 21dpf | 4 | 1.00±0.33 |

**Fig.7Ha**

**Step 1. Normal distribution analysis**

**Normality**

| **Shapiro-Wilk test** | | | | |
| --- | --- | --- | --- | --- |
| **Fig** | **W** | **df** | **P** | **Sig.** |
| 7-Ha | 0.899 | 8 | 0.285 | ns |

**normal distribution**

**Step 2. Independent sample T test (two-tail)**

| **Treatment** | **Comparison** | **t** | **P** | **Sig.** |
| --- | --- | --- | --- | --- |
| 50μg/g | *fabp2^-/-^* vs. *fabp2^+/+^* | 2.723 | 0.0025 | ***** |

**Fig.7Hb**

**Step 1. Normal distribution analysis**

**Normality**

| **Shapiro-Wilk test** | | | | |
| --- | --- | --- | --- | --- |
| **Fig** | **W** | **df** | **P** | **Sig.** |
| 7-Hb | 0.795 | 8 | 0.025 | ***** |

***nonnormal distribution**

**Step 2. Mann-Whitney test**

| **Treatment** | **Comparison** | **U** | **P** | **Sig.** |
| --- | --- | --- | --- | --- |
| PIVE-100μM | *fabp2^-/-^* vs. *fabp2^+/+^* | 0 | 0.0079 | ***** |

- **Fig. 7J**

| **Group** | **Age** | ***n*** | **M ± SEM** |
| --- | --- | --- | --- |
| MIA-50μg/g-*fabp2^-/-^* | 21dpf | 35 | 44.07±5.91 |
| MIA-50μg/g-*fabp2^+/+^* | 21dpf | 36 | 21.93±4.57 |
| PIVE-100μM-*fabp2^-/-^* | 21dpf | 48 | 26.02±4.19 |
| PIVE-100μM-*fabp2^+/+^* | 21dpf | 46 | 30.54±5.21 |
| Control WT-*fabp2^-/-^* | 21dpf | 34 | 21.16±4.92 |
| Control WT-*fabp2^+/+^* | 21dpf | 25 | 28.69±6.59 |

**Step 1. Normal distribution analysis**

**Normality**

| **Shapiro–Wilk test** | | | | |
| --- | --- | --- | --- | --- |
| **Fig** | **W** | **df** | **P** | **Sig.** |
| 7-J | 0.814 | 591 | 0.000 | ****** |

**nonnormal distribution**

**Step 2. compare different situation of KO within the same treatment**

**Control**

Kruskal-Wallis test

| **df** | **H** | **P** | **Sig.** |
| --- | --- | --- | --- |
| 2 | 1.785 | 0.4097 | ns |

**MIA-50**

Kruskal-Wallis test

| **df** | **H** | **P** | **Sig.** |
| --- | --- | --- | --- |
| 2 | 14.68 | 0.0006 | ** |

Bonferroni's multiple comparisons test

| **Treatment** | **Comparison** | **Z** | **P** | **Sig.** |
| --- | --- | --- | --- | --- |
| 50μg/g | *fabp2*^-/-^ vs. WT | 4.057 | 0.0006 | ** |
| 50μg/g | *fabp2*^+/+^ vs. WT | 0.6822 | 0.8709 | ns |
| 50μg/g | *fabp2*^+/+^ vs. *fabp2*^-/-^ | 2.963 | 0.0127 | * |

**PIVE-100**

Kruskal-Wallis test

| **df** | **H** | **P** | **Sig.** |
| --- | --- | --- | --- |
| 2 | 4.689 | 0.0959 | ns |

**Step 3. compare different treatment within the same KO**

**WT**

Kruskal-Wallis test

| **df** | **H** | **P** | **Sig.** |
| --- | --- | --- | --- |
| 2 | 16.14 | 0.0003 | ** |

Bonferroni's multiple comparisons test

| **Treatment** | **Comparison** | **Z** | **P** | **Sig.** |
| --- | --- | --- | --- | --- |
| WT | MIA-50μg/g vs. Control | 3.510 | 0.0013 | * |
| WT | PIVE-100μM vs. Control | 3.147 | 0.0049 | * |
| WT | PIVE-100μM vs. MIA-50μg/g | 0.01278 | >0.9999 | ns |

***fabp2*^-/-^**

Kruskal-Wallis test

| **df** | **H** | **P** | **Sig.** |
| --- | --- | --- | --- |
| 2 | 7.253 | 0.0266 | * |

Bonferroni's multiple comparisons test

| **Treatment** | **Comparison** | **Z** | **P** | **Sig.** |
| --- | --- | --- | --- | --- |
| *fabp2*^-/-^ | MIA-50μg/g vs. Control | 2.425 | 0.0306 | * |
| *fabp2*^-/-^ | PIVE-100μM vs. Control | 0.2951 | >0.9999 | ns |
| *fabp2*^-/-^ | PIVE-100μM vs. MIA-50μg/g | 2.297 | 0.0432 | # |

***fabp2*^+/+^**

Kruskal-Wallis test

| **df** | **H** | **P** | **Sig.** |
| --- | --- | --- | --- |
| 2 | 1.215 | 0.5447 | ns |
